# Longitudinal tracking and quantification of individual *Plasmodium falciparum* clones in complex infections

**DOI:** 10.1038/s41598-019-39656-7

**Published:** 2019-03-04

**Authors:** Anita Lerch, Cristian Koepfli, Natalie E. Hofmann, Johanna H. Kattenberg, Anna Rosanas-Urgell, Inoni Betuela, Ivo Mueller, Ingrid Felger

**Affiliations:** 1 Swiss Tropical and Public Health Institute, Basel, Switzerland; 2 University of Basel, Basel, Switzerland; 3 Walter and Eliza Hall Institute of Medical Research, Parkville, VIC Australia; 4 University of Melbourne, Parkville, VIC Australia; 5 Papua New Guinea Institute of Medical Research, Madang, Papua New Guinea; 6 Present Address: University of Notre Dame, Notre Dame, IN USA; 7 Present Address: Institute of Tropical Medicine, Antwerp, Belgium; 8 Present Address: Institut Pasteur, Paris, France

## Abstract

Longitudinal tracking of individual *Plasmodium falciparum* strains in multi-clonal infections is essential for investigating infection dynamics of malaria. The traditional genotyping techniques did not permit tracking changes in individual clone density during persistent natural infections. Amplicon deep sequencing (Amp-Seq) offers a tool to address this knowledge gap. The sensitivity of Amp-Seq for relative quantification of clones was investigated using three molecular markers, *ama1*-D2, *ama1*-D3, and *cpmp*. Amp-Seq and length-polymorphism based genotyping were compared for their performance in following minority clones in longitudinal samples from Papua New Guinea. Amp-Seq markers were superior to length-polymorphic marker *msp*2 in detecting minority clones (sensitivity Amp-Seq: 95%, *msp2:* 85%). Multiplicity of infection (MOI) by Amp-Seq was 2.32 versus 1.73 for *msp2*. The higher sensitivity had no effect on estimates of force of infection because missed minority clones were detected in preceding or succeeding bleeds. Individual clone densities were tracked longitudinally by Amp-Seq despite MOI > 1, thus providing an additional parameter for investigating malaria infection dynamics. Amp-Seq based genotyping of longitudinal samples improves detection of minority clones and estimates of MOI. Amp-Seq permits tracking of clone density over time to study clone competition or the dynamics of specific, i.e. resistance-associated genotypes.

## Introduction

Molecular-epidemiological parameters used to describe the infection dynamics of *Plasmodium falciparum* include the number of co-infecting parasite clones (multiplicity of infection, MOI), the rate at which different genotypes are acquired over time (molecular force of infection, _mol_FOI) and duration of infection^1^. These measures are based on monitoring the presence or absence of clones in cross-sectional or longitudinal samples collected in regular intervals. In earlier studies, individual parasite clones in multi-clonal field samples were distinguished and tracked over time by genotyping the length-polymorphic marker merozoite surface protein 2 (*msp2*) by capillary electrophoresis-based fragment sizing (CE)^2–4^. Yet, *msp2*-CE genotyping has limited sensitivity for minority clone detection^3,5,6^. Alternative typing methods instead could perform better in detecting minority clones, but might impact measures of MOI and _mol_FOI^7,8^. So far quantification of individual clones within multi-clonal infections was not feasible, as this would have required highly complex allele-specific quantitative PCR (qPCR).

SNP-based genotyping by deep amplicon sequencing (Amp-Seq) can detect low-abundant *P*. *falciparum* clones at ratios of 1:1000 in mixed infections^7–9^. Most importantly, genotyping by Amp-Seq also quantifies precisely the relative abundance of clones, as shown with artificial mixtures of clones^9–11^. From these ratios the absolute density of each clone (i.e. a certain haplotype) within a multi-clone infection can be deduced if the total parasitaemia of the sample was established by qPCR^11^. When analysing consecutive samples from a given study participant, presence and fluctuations in density of clones can be tracked. We explore how longitudinal information can be used to improve identification of minority clones with low densities around the detection limit.

A previous study has estimated clonal density with Amp-Seq in multi-clone infections to estimate clearance rates after antimalarial treatment^11^. We apply the same approach to track parasite clones longitudinally in untreated natural infections. In addition, we increase the resolution of genotyping by combining sequence information from several markers into multi-locus haplotypes.

## Methods

### Study design

A subset of 153 archived *P*. *falciparum* genomic DNA samples from 33 children (mean 4.3 samples [min: 2, max: 11]) aged 1–5 years were available from a cohort study described earlier^12^ with blood sampling over 40 weeks (first 12 weeks every fortnight, then monthly) in Papua New Guinea (PNG). The two conditions for selection of children were: ≥2/14 bleeds PCR positive, and MOI > 1 in at least one of the samples of each child. Ethical clearance was obtained from PNG Institute of Medical Research Institutional Review Board (IRB 07.20) and PNG Medical Advisory Committee (07.34). Informed written consent was obtained from all parents or guardians prior to recruitment of each child. All experiments were performed in accordance with relevant guidelines and regulations.

### Genotyping using length polymorphic marker *msp2*

Samples were genotyped using the classical *P*. *falciparum* marker *msp2* according to published protocols^13^. Fluorescently labelled nested PCR products were sized by CE on an automated sequencer and analysed using GeneMarker software. Fragments were accepted if the following cut-off criteria were met: peak height >500 intensity units and >10% of the height of the majority peak. Electropherograms were inspected visually to exclude obvious stutter peaks. All DNA samples were genotyped in 2 independent laboratories to assess reproducibility of clone detection and measures of MOI.

### Marker selection for Amplicon deep sequencing

Amp-Seq was performed on three amplicons located in two different *P*. *falciparum* marker genes, namely PF3D7_0104100, “conserved *Plasmodium* membrane protein” (*cpmp*), and PF3D7_1133400, “apical membrane antigen 1” (*ama1*) whose genetic diversity has been studied in great detail^7,14–16^. Previously published primers were used for marker *cpmp*^9^. For *ama1* two amplicons of 479 and 516 bp were selected that span regions of maximum diversity, i.e. subdomains 2 and 3 of the ectodomain^17^. Primer sequences and exact amplicon positions are listed in Supplementary Tables S1 and S2.

### Sequencing library preparation

Sequencing libraries were generated by three rounds of PCR, according to previously published protocols^9^. After primary PCR, a 5′ linker sequence was added during nested PCR. Nested PCR products were subject to another PCR round with primers binding to the linker sequences and carrying Illumina sequence adapters plus an eight nucleotide long sample-specific molecular index to permit pooling of amplicons for sequencing and later de-multiplexing. The final sequence library was purified with NucleoMag beads. Sequencing was performed on an Illumina MiSeq platform in paired-end mode (2 × 250 bp) using Illumina MiSeq reagent kit v2 (500-cycles) together with Enterobacteria phage PhiX control (Illumina, PhiXControl v3).

### Sequence read analysis and haplotype calling

Samples yielding a sequence coverage of <25 reads were excluded from the analysis. An overview of sequence read coverage for all Amp-Seq markers is given in Supplementary Table S3. Several pipelines to process Amp-Seq data have recently been published, including HaplotypR (https://github.com/lerch-a/HaplotypR.git) that was used for this study^9,18–21^. Haplotype calling is explained in full detail in an earlier publication^9^. In short: Low quality sequences were removed by trimming reads to a final size of 240 bp forward and 170 bp reverse for all amplicons. Index, linker and primer sequence (corresponding to ~50 bp) were trimmed off from forward and reverse reads. As the reference sequence, *P*. *falciparum* strain 3D7 was used (PlasmoDB release 34^22^). The term genotype refers to a single nucleotide polymorphism (SNP), whereas a haplotype was defined as a sequence variant of an entire amplicon. Calling a SNP required a >50% frequency of the total reads in at least two independent samples. Haplotypes containing insertions or deletions (indels) were filtered out, as well as haplotypes resulting from chimeric reads or singleton reads. The number of reads of a given haplotype over all remaining reads of the same marker within a sample is denoted by the term “within-host haplotype frequency”. Cut-off criteria for haplotype calling were as follows: a minimum of 3 reads coverage per haplotype, a within-host haplotype frequency ≥0.1% and an occurrence of this haplotype in ≥2 samples over the entire data set including technical replicates. The chosen cut-off criteria where studied in great detail and discussed in an earlier publication^9^.

### Multi-locus haplotype inference in longitudinal samples

Amp-Seq quantifies the frequency of each haplotype within a sample. This permits the inference of multi-locus haplotypes, an approach also used earlier by software DEploid^23^. In this study a semi-automated procedure was applied for multi-locus haplotype inference that utilized longitudinal sample information to solve complex mixtures. A multi-locus haplotype was deduced iteratively and separately for each sample. In the first round, the multi-locus haplotype of the dominant clone of a sample was inferred by selecting each marker’s dominant haplotype (>54% within-host haplotype frequency, i.e. 50% + 3.8% standard deviation in within-host haplotype frequency between replicates). After each round, the identified dominant haplotype was ignored and in the following round the dominant haplotype was identified among the remaining reads. If several haplotypes occurred in a sample at similar frequencies, it may be impossible to identify the dominant haplotype. Nevertheless, in many cases this could be resolved by analysing the change in within-host haplotype frequency between the observed and preceding or succeeding sample of the same host. An example of our approach to multi-locus haplotype inference is shown in detail in Supplementary Text.

The final step of multi-locus haplotype inference addressed the problem of clones from a multiple infection that share by chance the same allele of one of the markers. As a consequence, the within-host frequency of a shared haplotype amounts to the sum of two or more independent clones carrying the same allele. In such cases multi-locus haplotypes were inferred by assigning the shared alleles to those haplotypes that summed up to the same proportion in the other two markers. Samples for which the multi-locus haplotype could not be established by this approach were considered unresolvable (Supplementary Table S4).

### Reproducibility, sensitivity and false discovery rate

Samples were analysed in duplicate with Amp-Seq markers and *msp2*-CE. Performing duplicates permitted to identify and exclude false-positive haplotypes and thus prevented erroneous over-estimation of MOI. Each haplotype was classified into one of four groups (example see Supplementary Fig. S1): (1) True-positive (TP) haplotype, i.e. it passed the haplotype calling cut-off in both replicates or in one replicate plus in the preceding or succeeding bleed; (2) False-positive (FP) haplotype, i.e. it passed the haplotype calling cut-off in only one replicate and was not detected in any of the preceding or succeeding samples of that individual; (3) False-negative (FN_i_) haplotype, i.e. it was detected in one or both replicates but did not pass the cut-off criteria at that occasion, whereas it was detected in the preceding or succeeding bleed as TP (at least once) or FN haplotype; (4) Background noise (all other cases).

Additionally, false-negative (FN_ii_) haplotypes were imputed for samples in which no sequence read was detected. These false-negative haplotypes were imputed only when (a) the haplotype was detected in the preceding as well as the succeeding bleed as a true-positive. Presence in only one of preceding or succeeding sample was not considered sufficient evidence for assuming a case of missed detection. For the Amp-Seq markers but not *msp2*-CE, false-negative haplotypes were also imputed when (b) data for the other two markers was present and the corresponding multi-locus haplotype was established in the preceding or succeeding sample.

The sensitivity to detect parasite clones was estimated based on selected individuals who had not received antimalarial treatment during the timespan analysed and harboured at least one haplotype that was detected at 3 consecutive bleeds. Sensitivity was defined as the true positive rate of a genotyping method and was calculated as TP/(TP + FN). The risk to falsely assign a haplotype not present in the sample was measured as the “false discovery rate” (FDR), calculated as FP/(TP + FP). This rate represents the extent of false haplotype calls of a genotyping method.

The reproducibility of clone detection in technical replicates (comprising all experiential procedures from PCR to sequence run) was calculated as $\frac{2{n}_{2}}{{n}_{1}+2{n}_{2}}$, where *n*_*1*_ is the number of haplotypes detected in a single replicate and *n*_*2*_ the number of haplotypes detected in both replicates^24^. Only TP haplotypes were used to estimate reproducibility.

### Epidemiological parameters: clone density_,_ diversity, MOI and FOI

The density of a parasite clone was calculated by multiplying within-host haplotype frequency by parasitaemia (measured by qPCR). As late *P*. *falciparum* stages are absent from peripheral blood owing to sequestration, it was assumed that all detected clones were ring or early trophozoite stages, which each possess a single haploid genome. Thus, genome density correlates with clone density. Clone density is expressed as copies of target gene per microliter, quantified by qPCR targeting the 18S rRNA gene of *P*. *falciparum*^25^. The technical detection limit of qPCR was 0.4 copies/μl whole blood.

Based on true positive haplotypes, the expected heterozygosity (H_e_) and mean MOI were determined from baseline (or first bleed available) samples for each marker as described earlier^9^. H_e_ was also estimated for combined markers in samples that had a resolvable multi-locus haplotype and that were separated by a treatment plus ≥2 consecutive *P*. *falciparum* negative samples from the same child.

_mol_FOI was estimated on longitudinal sets of sample that had a complete set of replicates for all markers. Haplotypes were counted as new infection if a haplotype was (i) not present in the baseline sample but in a subsequent sample, (ii) not detected at ≥2 consecutive preceding bleeds or (iii) not detected after antimalarial treatment plus after at least one negative sample. Time at risk was calculated as the timespan between baseline and last sampling, minus 14 days for each antimalarial treatment (to account for the prophylactic effect of treatment).

An overview of sample selection criteria applied for different types of analyses is listed in Supplementary Table S5.

## Results

### Genetic diversity of markers

**Figure 1:**
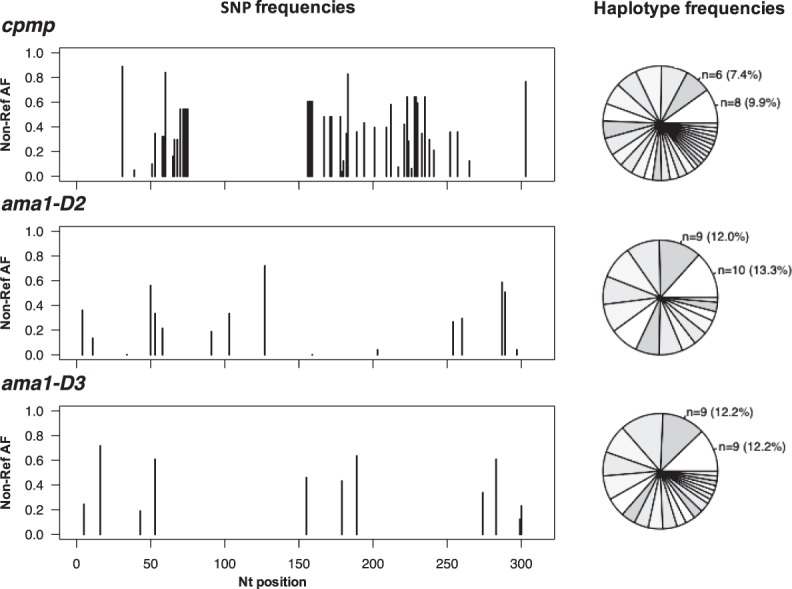
Frequency of individual SNPs and haplotypes of three markers in 33 baseline samples from PNG. Non-reference allelic frequency (Non-Ref AF) of each SNP (left) and frequency of haplotypes in these baseline samples (right). n, number of observations per haplotype shown for 2 most prevalent haplotypes. Total number of different haplotypes: 30 for *cpmp*, 15 for *ama1*-D2 and 22 for *ama1*-D3. (Frequency of haplotypes for markers *msp2*-CE given in Supplementary Fig. S2).

The discriminatory power of Amp-Seq markers *cpmp*, *ama1*-D2 and *ama1*-D3, as well as length-polymorphic marker *msp2*-CE was estimated in 33 baseline samples (Supplementary Table S5). The resolution was highest for amplicon marker *cpmp* (H_e_ = 0.961) that distinguished 30 haplotypes and yielded a mean MOI = 2.45 (Table 1, MOI distribution by marker in Supplementary Fig. S2). The second-best resolution was obtained by marker *msp2*-CE (H_e_ = 0.940) that distinguished 20 haplotypes and measured a mean MOI = 1.73. Haplotype and SNP frequencies of Amp-Seq markers are shown in Fig. 1 and Supplementary Fig. S2.

**Table 1:** Genotyping results of 4 molecular markers analysed in 33 baseline field samples. H_e_, expected heterozygosity. MOI, multiplicity of infection.

Marker	H_e_	Mean MOI	Number of clones^a^	Number of haplotypes	Number of SNPs^b^
*msp2* CE	0.940	1.73^c^	57	20	n/a
*cpmp*	0.961	2.45^c^	81	30	48
*ama1*-D2	0.928	2.27^c^	75	15	17
*ama1*-D3	0.939	2.24^c^	74	22	11

^a^Sum of all haplotypes in all samples.

^b^With respect to the reference sequence of *P*. *falciparum* strain 3D7.

^c^Pairwise comparison using two-sided paired t-test with adjusted p-value by Holm: p-value = 0.008 for *ama1*-D2 vs *msp2*-CE, p-value = 0.036 for *ama1*-D3 vs *msp2*-CE, and p-value = 0.005 for *cpmp* vs *msp2*-CE.

Discriminatory power can be increased by combining multiple markers. Inference of multi-locus haplotypes was not possible for all baseline samples. Instead, 47 independent samples were analysed that had fully established multi-locus haplotypes (Supplementary Table S5). These 47 samples comprised 67 fully established multi-locus haplotypes. Combining marker *cpmp* with either of the two *ama1* fragments yielded very high diversity (54 and 56 haplotypes, H_e_ = 0.992 and 0.994 for *cpmp*/*ama1*-D2 and *cpmp*/*ama1*-D3) (Table 2 and Supplementary Fig. S3). Combining all 3 markers did not increase discriminatory power any further.

**Table 2:** Genotyping results of 3 molecular markers analysed in 47 independent field samples with 67 different clones. H_e_, expected heterozygosity.

Marker	H_e_	Number of Haplotypes	Mean MOI
*cpmp*	0.948	25	1.43
*ama1-D2*	0.925	16	1.30
*ama1-D3*	0.936	21	1.30
*cpmp* + *ama1-D2*	0.992	54	1.43
*cpmp* + *ama1-D3*	0.994	56	1.43
*cpmp* + *ama1-D2* + *ama1-D3*	0.994	56	1.43

### Using longitudinal genotyping data to increase detectability of clones

Imperfect detectability of parasite clones has been described previously in longitudinal genotyping studies^1,26–28^. Data from replicates and longitudinal samples can be used to make assumptions on missed clones. This permits imputing of missed haplotypes and thus improves the tracking of clonal infections within an individual over time. Two types of false-negative haplotypes were distinguished: (FN_i_) haplotypes that were detected below the cut-off and (FN_ii_) haplotypes that were not detected but imputed (Supplementary Table S6). Supplementary Fig. S4 shows an example of these different types of missed haplotypes for all Amp-Seq markers.

The sensitivity to detect parasite clones was estimated for each genotyping marker by enumerating false-negative haplotypes. Sensitivity was higher for the Amp-Seq markers than for *msp2*-CE (in decreasing order 96.5%, 95.0%, 93.9% and 85.1% for *ama1*-D2, *cpmp*, *ama1*-D3 and *msp2*-CE) (Table 3). For ≥57% of the identified false-negative haplotypes, reads were detected but fell below cut-off criteria (category (i) above). If such haplotypes were counted as positives by relaxing the cut-off criteria, sensitivity would increase to 99.1%, 97.5% and 97.4% for Amp-Seq markers *ama1*-D2, *cpmp* and *ama1*-D3 (Table 3). Using the standard cut-off criteria, the false discovery rate of haplotypes for Amp-Seq markers was in the range of 0.9–4.2% (Table 3).

**Table 3:** Sensitivity and false discovery rate (FDR) of the genotyping method.

Marker	TP	FN			FP	Sensitivity	FDR	Detected Haplotypes^a^
	n	n_i_	n_iia_	n_iib_	n	TP/(TP + FN_i+iiab_)	FP/(TP + FP)	(TP + FN_i_)/(TP + FN_i+iiab_)
*msp2*-CE	86	10	5	n/a^b^	n/a^c^	0.851 ± 0.101^d^	n/a^c^	0.950 ± 0.061^d^
*cpmp*	115	4	2	1	5	0.943 ± 0.066	0.042 ± 0.057	0.975 ± 0.044
*ama1*-D2	109	3	0	1	1	0.965 ± 0.052	0.009 ± 0.027	0.991 ± 0.026
*ama1*-D3	108	4	2	1	3	0.939 ± 0.068	0.027 ± 0.046	0.974 ± 0.045

Sensitivity and FDR including 95% confidence interval was estimated based on persistent clones in 48 longitudinal samples from 12 individuals. Detectability of minority clone can be increased by including missed persistent haplotypes detected below the cut-off criteria. TP, true-positive haplotypes. FN_i_, false-negative haplotypes detected, but below cut-off criteria. FN_iiab_, false-negative haplotypes with no read detected.

^a^Detected true-positive and false-negative haplotypes.

^b^Not imputed for *msp2*-CE as multi-locus haplotypes cannot be established.

^c^Length-polymorphic data generated in different laboratories do not provide replicates suited for determination of false-positive haplotype calls and estimation of FDR.

^d^Without haplotypes, that were imputed based on multi-locus haplotypes at the beginning or end of an infection.

Reproducibility to detect parasite clones in technical replicates was greater for Amp-Seq markers than for marker *msp2*-CE (0.94, 0.93, 0.92 and 0.89 for *ama1*-D3, *ama1*-D2, *cpmp* and *msp2*-CE) (Supplementary Table S7). Reproducibility decreased either with decreasing clone density, decreasing within-host haplotype frequency, or decreasing sequence coverage (Supplementary Table S8 and Fig. S5)^9^. Differences in estimates of within-host haplotype frequency between replicates were very small: The median difference was 0.70%, 0.54% and 0.38% for *cpmp*, *ama1*-D3 and *ama1*-D2 (Supplementary Fig. S6).

### Determination of _mol_FOI by different molecular markers and methods

A higher sensitivity of the genotyping method does not necessary impact _mol_FOI, i.e. new clones/year, because a missed minority clone could be detected at one of the successive bleeds. We investigated the number of new infections acquired during 40 weeks follow-up in 27 children from whom a complete data set was available (on average 4.3 samples per child [min: 2, max: 7]) (Supplementary Figs S7–S39). Mean _mol_FOI was 2.7, 2.7, 2.3 and 2.2 new infections per year for markers *ama1*-D3, *cpmp*, *msp2*-CE and *ama1*-D2 (negative binomial regression p-value for comparison of *msp2*-CE to *ama1*-D3, *cpmp* and *ama1*-D2: 0.596, 0.649 and 0.877) (Supplementary Fig. S40). Thus, no substantial difference in mean _mol_FOI was found for the different molecular markers and different genotyping methods. Mean _mol_FOI of multi-locus haplotypes could not be calculated because multi-locus haplotype inference was not possible for all consecutive samples of each child (Supplementary Table S4).

### Quantitative dynamics of multiple infecting *P*. *falciparum* clones

**Figure 2:**
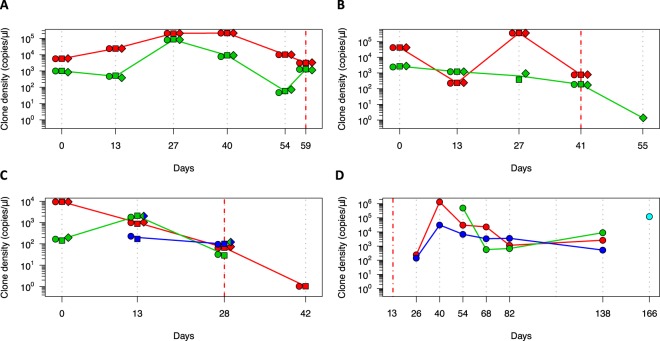
Dynamics of multi-clone infections in 4 children. Multi-marker haplotypes could be generated in panels A, B and C. Inference of multi-locus haplotypes was not possible for the child in panel D; here the dynamics of individual clones tracked by marker ama1-D2 are shown. Each colour represents a clone. Individual markers are represented by different shapes: cpmp (diamonds), ama1-D2 (circles) and ama1-D3 (squares). Solid line connecting multi-locus haplotypes represents their median frequency. Grey dotted vertical lines represent sampling dates. Red dashed lines represent day of artemisinin combination therapy. Red dash-dotted line represents end of radical cure (artesunate-primaquine) at baseline.

Densities of individual clones was calculated from the total parasitaemia by qPCR and the within-host haplotype frequency. Examples of individual clone density dynamics in children with multi-clone infections are shown for three Amp-Seq markers (Fig. 2). The density of some clones remained constant over time, whereas other clones showed fluctuations in density over 3 orders of magnitude (Fig. 2A,B). In some children the dominant clone remains dominant over the observation period (Fig. 2A), whereas in others switch-over between minority clone and dominant clone was observed (Fig. 2B). In highly complex field samples some clones might share the same haplotype of a given marker (Fig. 2C). Such clones can only be differentiated and quantified if multiple markers are typed and at least one of the markers is not shared between concurrent clones.

After artemisinin combination therapy, some of the parasite clones from multi-clone infections were cleared 14 days after antimalarial treatment, whereas others were still detectable (Fig. 2A–C). These persisting clones had decreased clone densities (<21 copies/μl) and likely represent remaining late gametocyte stages of cleared asexual infections^29^. Some new infections following antimalarial treatment (artesunate-primaquine) showed a rapid increase in clone density within the first 14 days after re-infection of a host, followed by a slow decrease in clone density until clearance (Fig. 2D), whereas in other infections clone density remained constant (Fig. 2C).

## Discussion

While MOI and _mol_FOI have been extensively described as epidemiological parameters, the ratio and density of individual clones within complex infections has not yet been investigated in detail. This gap in knowledge was due to shortfalls of traditional length-polymorphic markers, where the length of a fragment greatly influences the amplification efficiency in multi-clone infections with fragments competing in PCR and a strong bias favouring smaller fragments^5^. As a result, multi-locus haplotypes could not be inferred from traditional genotyping data in a reliable way. Such inference is required, for example, for phylogenetic or population genetic studies. In these studies, multiple-clone infections were usually excluded or only the predominant haplotype included^30,31^. With the possibility to establish multi-locus haplotypes from complex infections the discriminatory power will be greatly improved in future. This study explored the feasibility of multi-locus haplotype calling in complex infections and the usefulness of the Amp-Seq genotyping technique in longitudinal data.

Single Amp-Seq markers *cpmp*, *ama1*-D2, *ama1*-D3, and *msp2*-CE yielded similar resolution. Combining *cpmp* with either of the *ama1* fragments increased further discriminatory power. The excellent performance of Amp-Seq marker *cpmp* had been demonstrated earlier^9^. Such increased resolution is of great practical value for PCR-correction in clinical drug efficacy trials, where new infections need to be reliably distinguished from those present in an individual earlier^6,32,33^. Discriminatory power may be increased even further by replacing one of the two *ama1* fragments with another highly discriminatory marker that has no linkage to either Amp-Seq marker *cpmp* nor *ama1*.

Reproducibility of true-positive haplotype calls was measured based on two technical replicates. By definition, a true haplotype must occur in all replicates except for three cases: (1) imperfect detectability of low-density clones, where scarce template may, by chance, lead to occasional absence of the PCR template in one of the replicates, (2) template competition impeding minority clones, whereby templates of a minority clone, present at very low abundance, are outcompeted by dominant clones, and (3), insufficient sequence depth to detect the minority clone in one replicate. It is essential to differentiate between false-positive haplotype calls (caused by cross-contamination, or amplification and sequencing errors^9,11^) and imperfect detection. This was achieved by considering preceding or succeeding bleeds of an individual. This approach was applied for those cases only where a haplotype was missed in one of the replicates. In our data set, all missing haplotype calls of replicates could be assigned to one of the three causes: imperfect detection, template competition or insufficient sequence depth.

Genotyping longitudinal samples in duplicates enabled also an evidence-based approach to identify false-negative haplotypes. This permitted the estimation of each marker’s sensitivity to detect minority clones. The estimated sensitivities of minority clone detection should serve primarily for a comparison of different genotyping methods, as the sample’s true haplotype composition remain uncertain. Amp-Seq genotyping with markers *ama1*-D2, *ama1*-D3 and *cpmp* missed fewer clones compared to *msp2*-CE genotyping (Amp-Seq in average 5.4% versus 14.9% *msp2*-CE). This difference is likely due to less stringent cut-off criteria for Amp-Seq compared to *msp2-CE* genotyping. Minority clone detection by *msp2*-CE is limited by peak calling cut-off criteria, which are usually a fixed minimal signal intensity plus a minimum peak height of 10% (used in our study) or more of the dominant peak. Minority clones with an abundance of <10% of all amplified fragments will not pass these criteria. An increase of *msp2*-CE sensitivity would require a lower cut-off, which would lead to more false positive signals from either stutter peaks or background noise. In contrast, Amp-Seq allows the removal of PCR artefacts before haplotype calling and thus can support a much lower cut-off of <1%^9^.

In cohort studies where Amp-Seq genotyping is performed in successive follow up samples of the same patient, an even more relaxed definition of Amp-Seq cut-off criteria would be justifiable. In this scenario, the same evidence-based strategy of using successive samples can be used to recover minority haplotypes that were detected with read counts below the haplotype calling cut-offs. If recovery would be performed in this study, ≥57% of all false-negative haplotypes would be identified. Such recovery would increase detectability of parasite clones by Amp-Seq to >97%. In addition, multi-locus haplotypes could provide additional evidence for accurate recovery.

The higher sensitivity of Amp-Seq to detect minority clones compared to *msp2-*CE substantially increased MOI, but did not affect mean _mol_FOI. Any estimation of _mol_FOI needs to account for temporary absence of clones from the peripheral blood caused by sequestration^1,26–28^. A clone that is temporarily undetectable owing to density fluctuations is likely observed at either the preceding or succeeding bleed. Therefore, a clone is usually only counted as new infection if it was not detected in ≥2 consecutive blood samples. As a consequence, a clone missed at a single bleed will not necessarily lead to a decrease of _mol_FOI.

A clone that was intermittently missed at one bleed by *msp2-*CE was always detected by Amp-Seq. This observation supports the practice in earlier publications where intermittently missed clones were imputed^27,28^. Counting a recurrent haplotype as new infection after a single negative bleed would lead to an overestimation of _mol_FOI^1,3,26–28^. The statistical power of the current study was limited and a larger sample size is needed to fully explore the effect of the typing method used on estimates of MOI or _mol_FOI.

A major advantage of Amp-Seq over *msp2*-CE is that the density of an individual clone in multi-clone infections can be calculated. Quantifying the density of individual parasite clones over time permits the studying of dynamics, and thus fitness, of parasite clones exposed to within-host competition^34^. For example, the relative densities of new infections can be compared to clones already persisting in a host, and their densities in respect to extrinsic factors or clinical symptoms can be investigated.

For infections with high multiplicity (MOI ≥ 3), inference of multi-locus haplotypes remains challenging (example in Supplementary Fig. S41). Inference is straightforward if a haplotype occurs at a distinctive abundance in any of the longitudinal samples (Supplementary Table S4). In contrast, if haplotypes are equally abundant in one sample and also remain so over several time points, the multi-locus haplotype cannot be inferred. Inference also is impossible for complex patterns with shared haplotypes, i.e. if a haplotype has a high population frequency and therefore is present in 2 or more clones of a blood sample. Shared haplotypes may even lead to inference of wrong multi-locus haplotypes, e.g. when three clones were present at an equal within-host frequency, though only two haplotypes were measured at each locus. However, the risk of erroneous multi-locus haplotype inference decreases if more than 2 unlinked markers are used, as the likelihood of shared multi-locus haplotypes decreases with increasing number of loci. In the present study, multi-locus haplotypes up to MOI = 3 were inferred. For multiplicity >3 and for resolving complex patterns of shared haplotypes, additional longitudinal samples could be analysed simultaneously, for example by incorporating the within-host haplotype frequencies of all consecutive samples of an individual into DEploid software^23^.

## Conclusion

Amplicon sequencing improves clone detectability compared to *msp2*-CE owing to its greater sensitivity for detection of minority clones. Our results confirm earlier assumptions on clone persistence with intermittent missed observation. This validates the imputation of false negatives to correct for imperfect detection of clones, a strategy also used in previous studies on clone dynamics. Using multi-locus haplotypes for genotyping permitted to identify robustly individual clones and improved differentiation between new and recurring clones. Construction of multi-locus haplotypes are of great value to compensate the effects of highly abundant haplotypes in the population. The option to quantify individual clones enables new approaches to investigate effects of parasite fitness or superinfection in multi-clone infections.

## Supplementary information

Supplementary Material

## Data Availability

The datasets generated and analysed during the current study are available in NCBI Sequence Read Archive repository under accession number SRX2704363 (https://www.ncbi.nlm.nih.gov/sra/SRX2704363). The source code for software HaplotypR is available at https://github.com/lerch-a/HaplotypR.
