# Supplementary material for: Longitudinal tracking and quantification of individual Plasmodium falciparum clones in complex infections
Source: Sci Rep. 2019 Mar 4;9:3333. doi: 10.1038/s41598-019-39656-7 (PMC6399284; doi:10.1038/s41598-019-39656-7)
Supplement: Supplementary file 1 — Supplementary Material [file 41598_2019_39656_MOESM1_ESM.pdf]

Supplemental Material

Longitudinal tracking and quantification of individual *Plasmodium falciparum* clones in complex infections

Anita Lerch[1,2,3,#], Cristian Koepfli[3,4,#], Natalie E. Hofmann[1,2], Johanna H. Kattenberg[5,#], Anna Rosanas-Urgell[5,#], Inoni Betuela[5], Ivo Mueller[3,4,#], Ingrid Felger[1,2,*]

[1] Swiss Tropical and Public Health Institute, Basel, Switzerland
[2] University of Basel, Basel, Switzerland
[3] Walter and Eliza Hall Institute of Medical Research, Parkville, VIC, Australia
[4] University of Melbourne, Parkville, Australia
[5] Papua New Guinea Institute of Medical Research, Madang, Papua New Guinea

[#]current affiliations: AL, CK: University of Notre Dame, Notre Dame, USA. JHK, ARU: Institute of Tropical Medicine, Antwerp, Belgium. IM: Institut Pasteur, Paris, France.

* Corresponding author: Ingrid Felger, PhD

Table of Contents: page

Supplemental Figures **2**

Supplemental Tables **42**

Supplemental Text **46**

Supplemental Figures

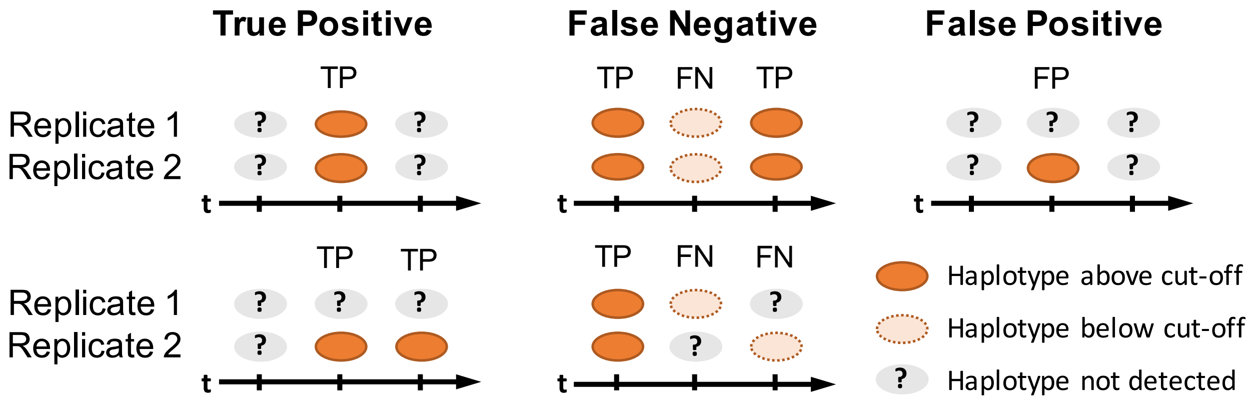

Supplementary Figure S1: Schematic of haplotype classification. Examples show the classification of haplotypes in true-positive (TP), false-negative (FN) and false-positive (FP), based on their detection either in duplicates or in the preceding or succeeding bleeds.

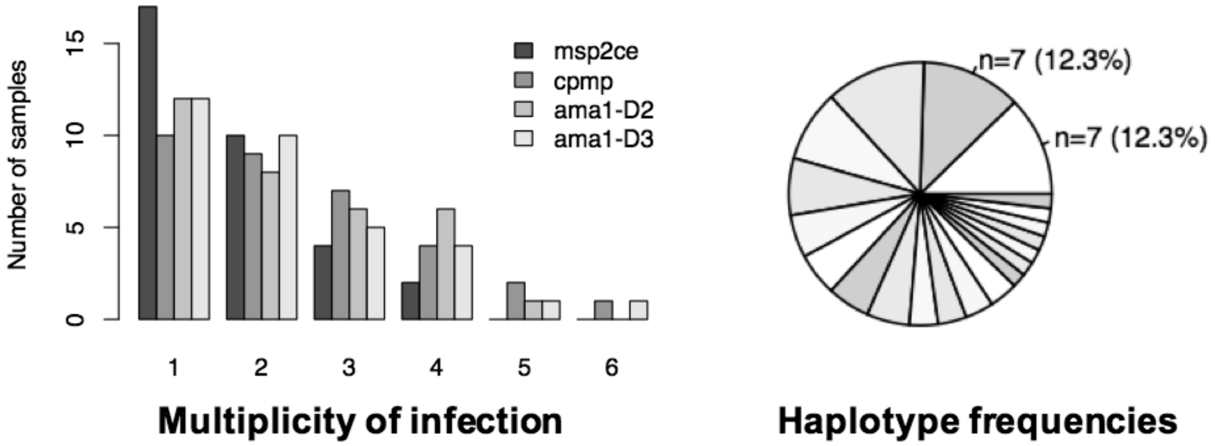

Supplementary Figure S2: Frequency distribution of multiplicity of infection by marker (left) and frequency of *msp2*-CE haplotypes (right) in 33 baseline samples. Marker *msp2*-CE identified 20 different haplotypes. (Frequency distribution of haplotypes of Amp-Seq markers given in Figure 1).

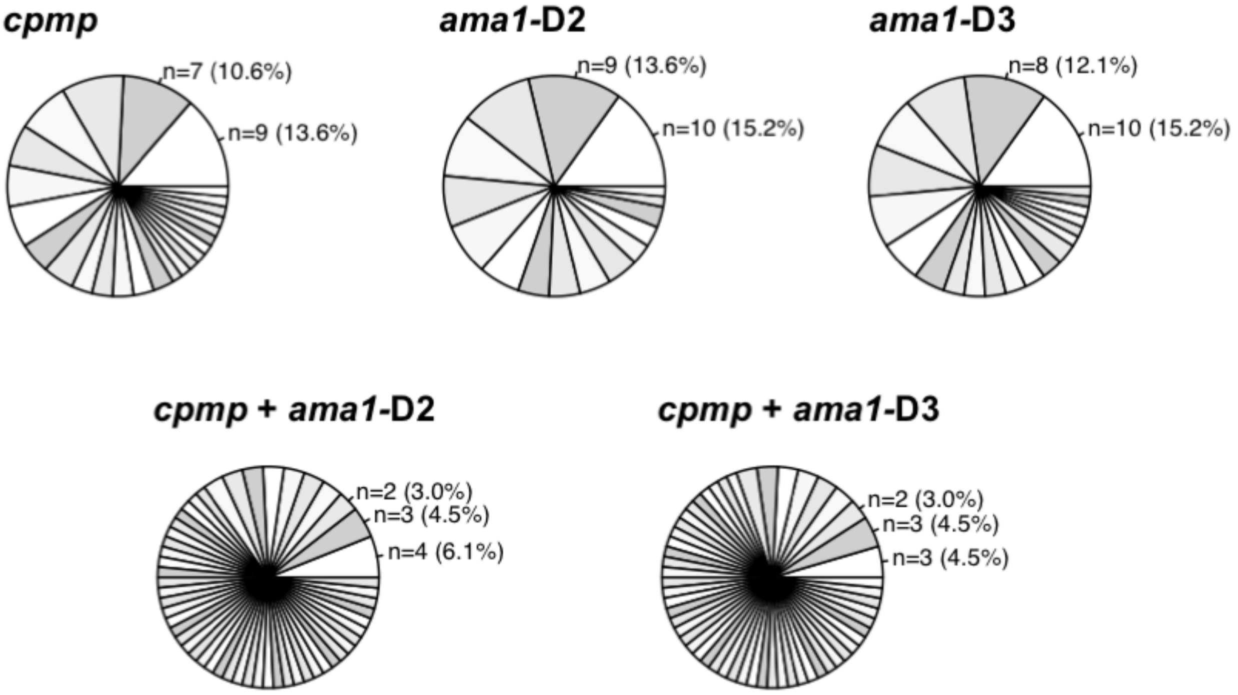

Supplementary Figure S3: Haplotype frequencies by marker in 47 independent samples comprising 67 clones. For marker *cpmp* 25 different haplotypes were identified, for *ama1*-D2 16 haplotypes and for *ama1*-D3 21 haplotypes. Frequencies of the most dominant haplotypes are indicated. Top panel: haplotypes based on single markers; bottom panel: two-marker haplotypes.

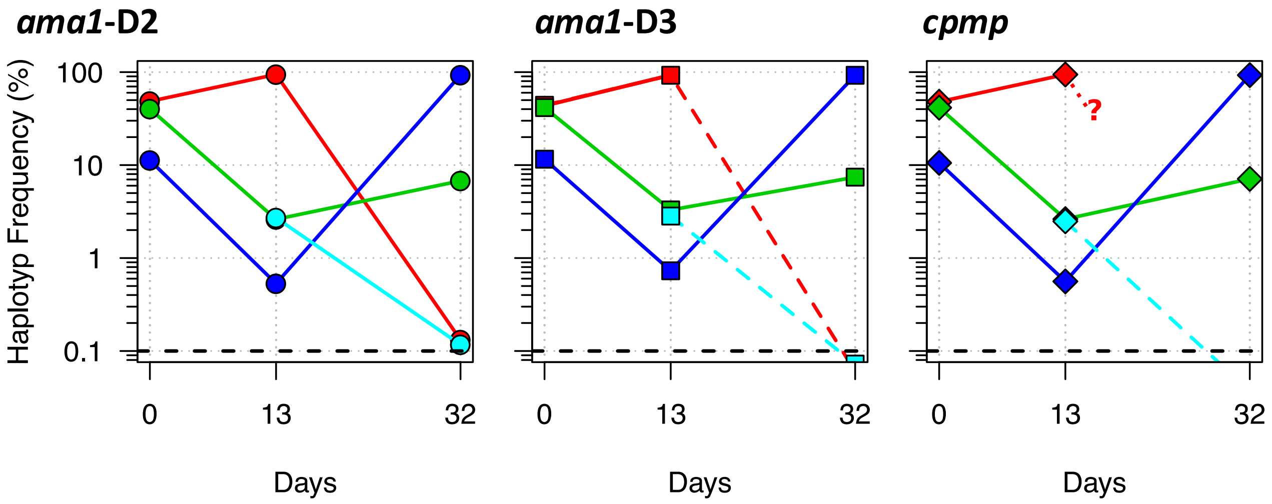

Days	Clone 1 ●	Clone 1 ■	Clone 1 ◆	Clone 2 ●	Clone 2 ■	Clone 2 ◆	Clone 3 ●	Clone 3 ■	Clone 3 ◆	Clone 4 ●	Clone 4 ■	Clone 4 ◆
0	48.5	44.0	48.0	40.2	41.5	41.4	11.3	11.6	10.6	-	-	-
13	94.4	92.6	94.3	2.61	3.29	2.62	0.53	0.73	0.56	2.71	2.83	2.49
32	0.13	0.06	n.d.	6.77	7.42	7.10	93.1	92.5	92.9	0.12	0.07	0.04

Supplementary Figure S4: Within-host haplotype frequencies of Amp-Seq markers in longitudinal samples from one child. Inserted table lists within-host multi-locus haplotype frequencies in percent. Multi-locus haplotypes have the same colour-code in figures and table. Solid line represents persisting haplotypes above cut-off criteria (true-positive haplotypes). Dashed line represents persisting haplotypes falling below cut-off criteria (false-negative haplotypes detected below cut-off criteria). Dotted line and question mark indicate a false-negative haplotype that was not detected (n.d.) but could be imputed based on the established multi-locus haplotypes from the preceding sample. Black dashed line represents cut-off criteria of the Amp-Seq genotyping method.

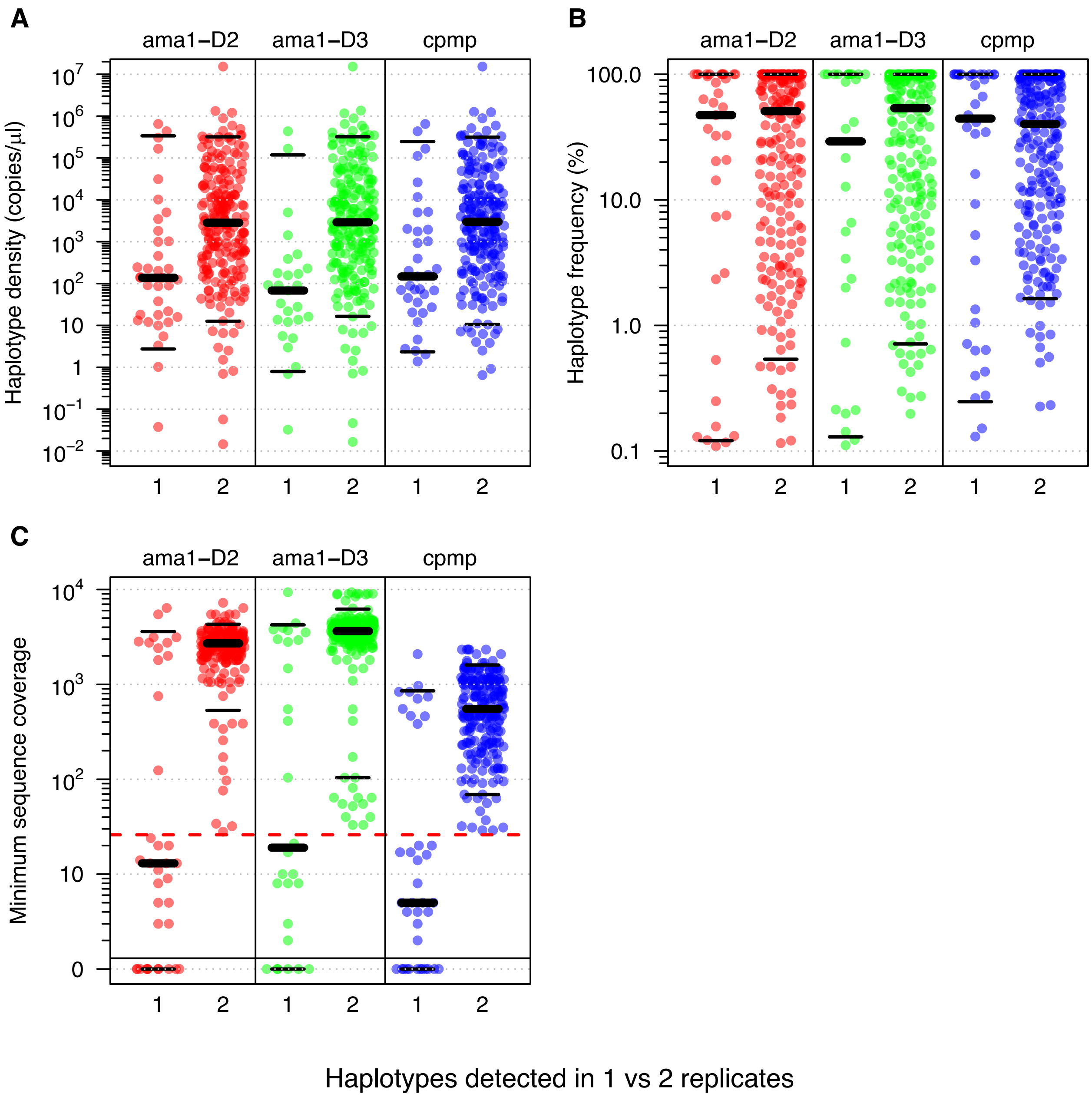

Supplementary Figure S5: Reproducibility of true-positive haplotypes detected in technical replicates. X-axis, haplotypes detected in 1 versus 2 replicates by 3 Amp-Seq markers. Dots represent individual haplotypes; colours represent individual markers. Black horizontal bars represent 5, 50 (bolded) and 95-percentiles. (**A**) Density of haplotypes detected in only one or both replicates. Y-axis, haplotype density by qPCR measured as 18S rRNA gene copies per µl whole blood. Wilcoxon rank sum test with continuity correction: W=2278 and p-value=4.4×10^{-5} for *ama1*-D2, W=1145 and p-value=1.6×10^{-7} for *ama1*-D3, W=2384 and p-value=1.8×10^{-4} for *cpmp*. (**B**) Within-host haplotype frequency detected in only one or both replicates. Y-axis, mean within-host haplotype frequency. Wilcoxon rank sum test with continuity correction: W=4212 and p-value=0.96 for *ama1*-D2, W=3038 and p-value=0.62 for *ama1*-D3, W=4431 and p-value=0.99 for *cpmp*. (**C**) Total sequence coverage of replicates detected in only one or both replicates. Y-axis, minimum sequence coverage across the two replicates. Zero represent samples without sequence reads. Red dashed line represents minimum sequence coverage criteria of 25 reads. Wilcoxon rank sum test with continuity correction: W=1370 and p-value=4.0×10^{-11} for *ama1*-D2, W=1163 and p-value=3.4×10^{-8} for *ama1*-D3, W=1331 and p-value=4.9×10^{-12} for *cpmp*.

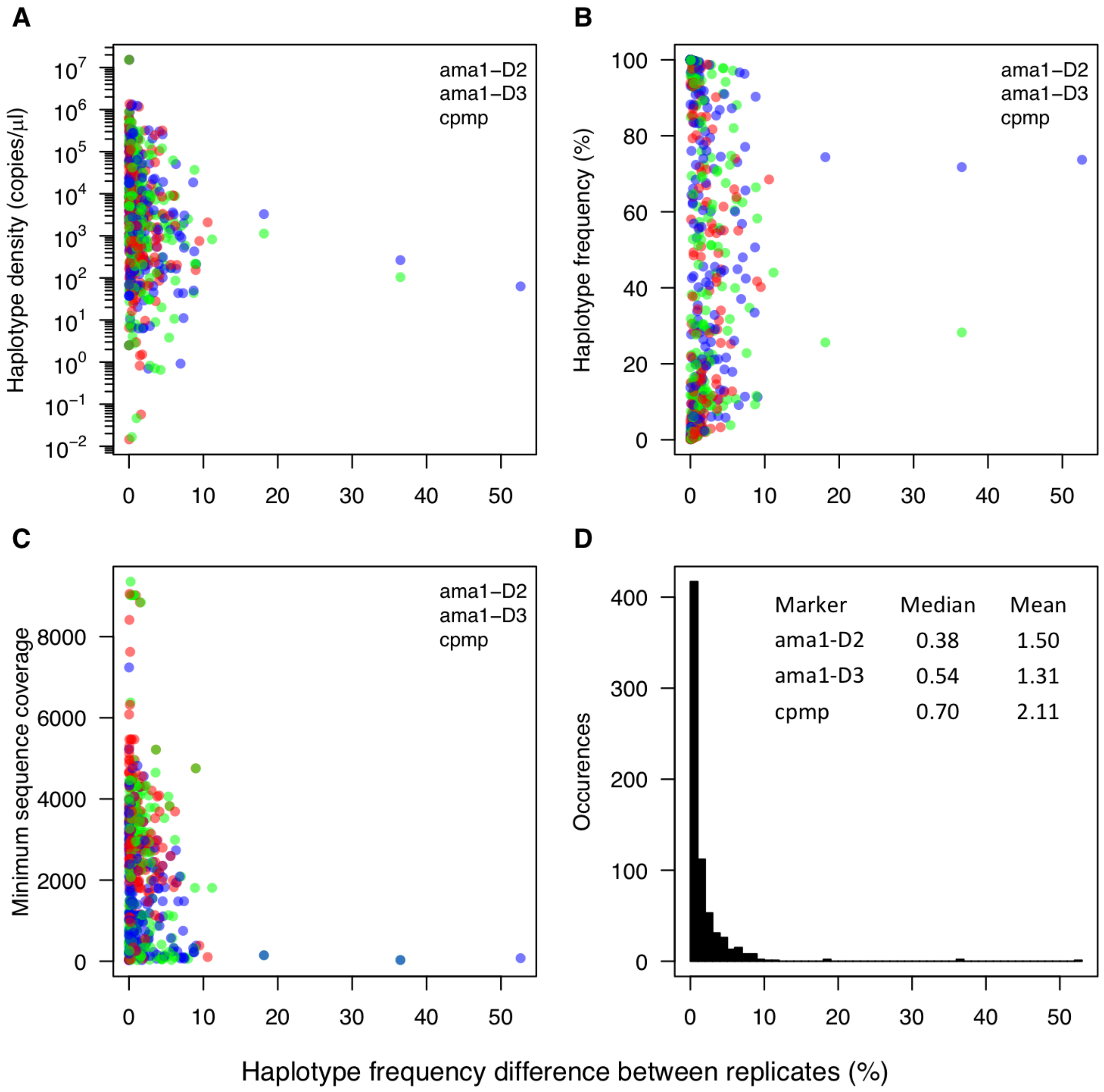

Supplementary Figure S6: Difference of within-host haplotype frequency between replicates versus (A) haplotype density (B) within-host haplotype frequency (C) sequence coverage. X-axis, difference in within-host haplotype frequencies between replicates. (A, B and C) Dots represent individual haplotypes; colours represent individual markers. (A) Y-axis, haplotype density by qPCR measured as 18S rRNA gene copies per µl whole blood. (B) Y-axis, within-host haplotype frequency (mean of both replicates). (C) Y-axis, minimum sequence coverage across the two replicates. **(D)** Distribution of the within-host haplotype frequency difference between replicates. Mean and median are indicated per individual marker.

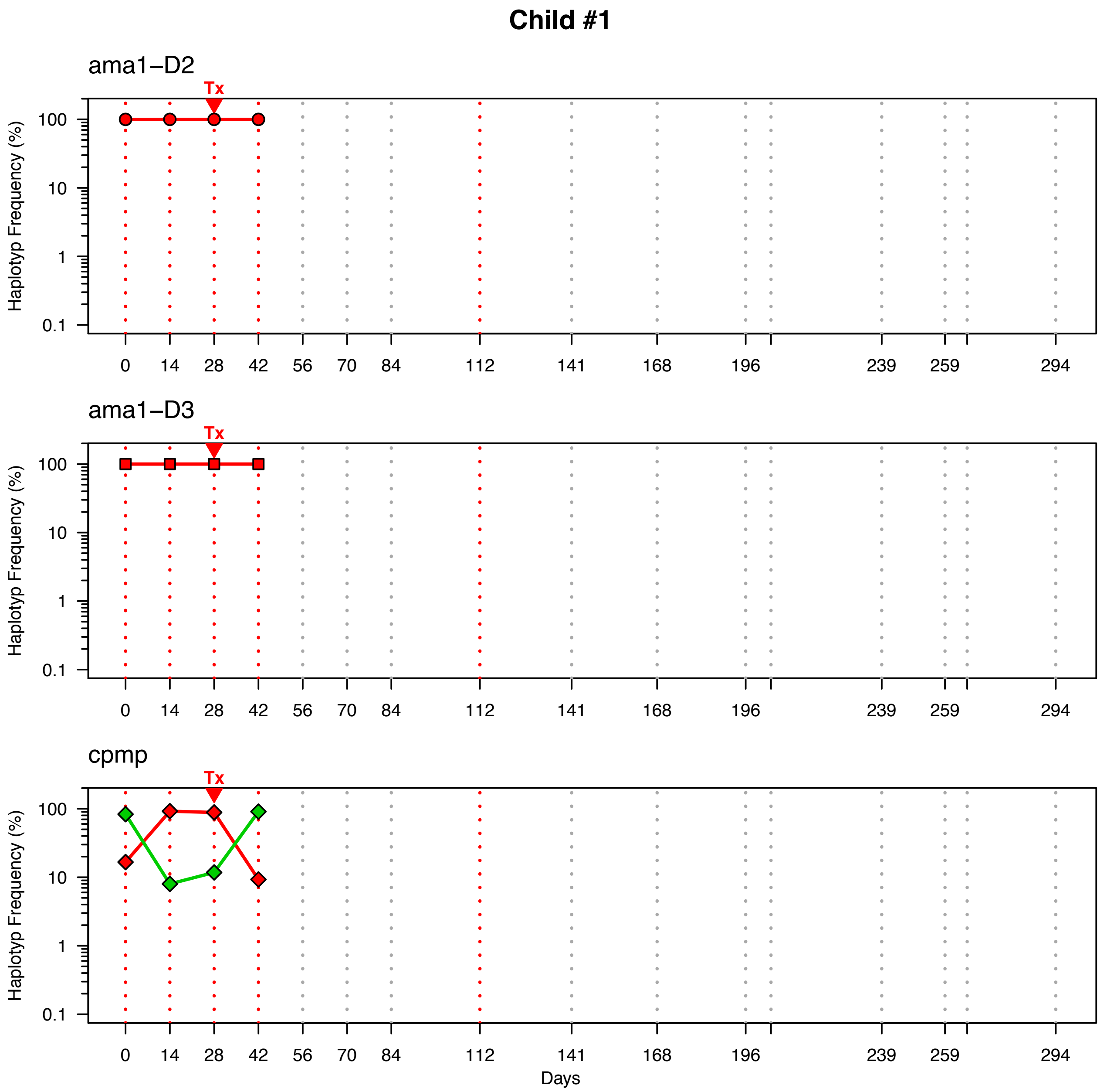

Supplementary Figure S7: Dynamics of within-host haplotype frequencies tracked by Amp-Seq markers in longitudinal samples from 33 children. For each child 3 markers were genotyped, which are distinguished by different shapes: *ama1*-D2 (circles), *ama1*-D3 (squares) and *cpmp* (diamonds). Different haplotypes of each marker are represented by the various colours of symbols. A solid line connecting haplotypes represents a persisting haplotype. Dotted lines connect haplotypes that were intermittently undetected, e.g. persisting haplotypes under the detection threshold or reinfection of the same haplotype. Vertical dotted lines represent sampling dates. The colour of these lines indicate whether the sample was *P. falciparum* positive (red) or negative (gray) by qPCR. Red triangle point down represent treatment. Y-axis, within-host haplotype frequency.

Child #2

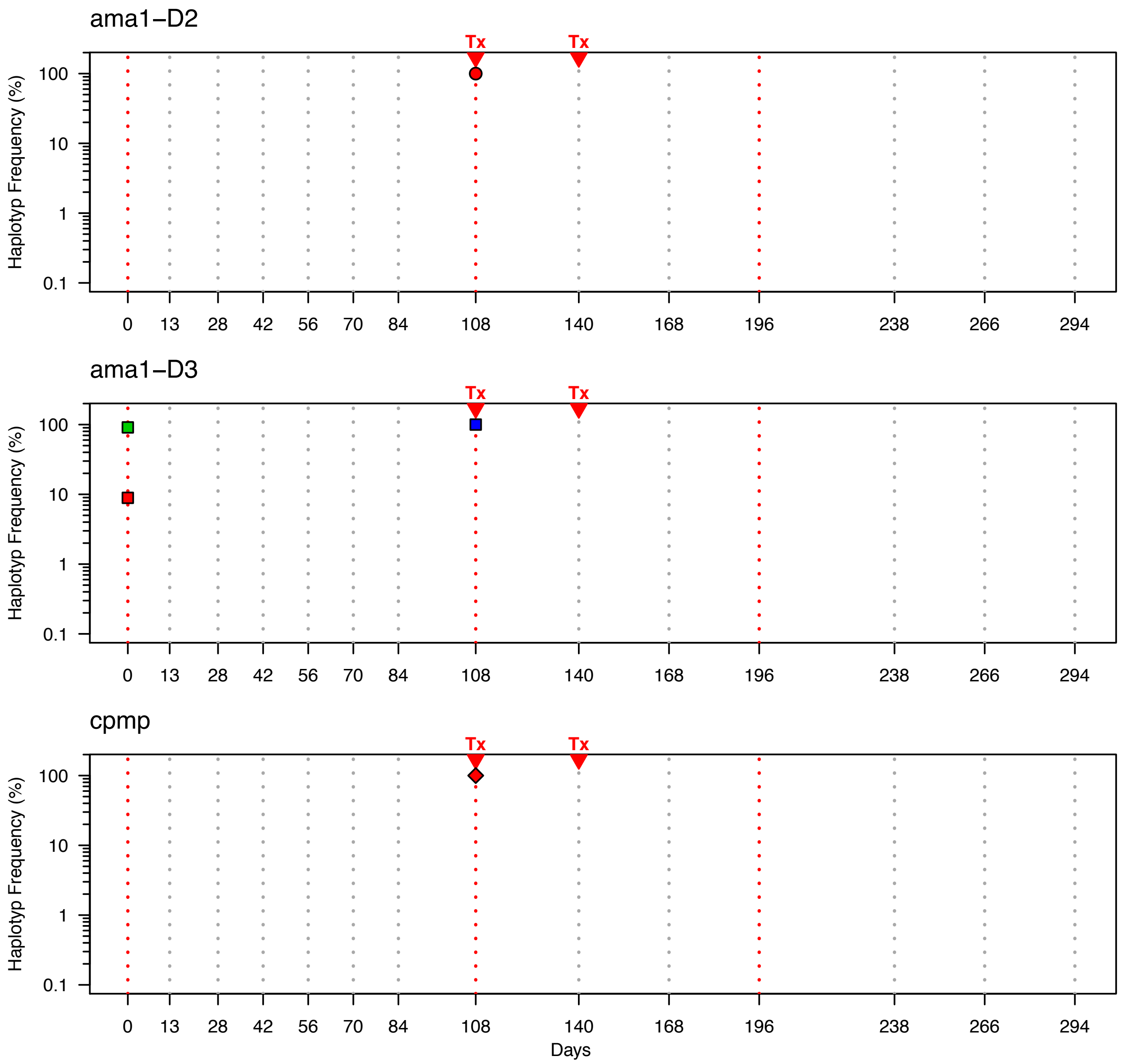

Supplementary Figure S8: Dynamics of within-host haplotype frequencies tracked by Amp-Seq markers in longitudinal samples from 33 children. For each child 3 markers were genotyped, which are distinguished by different shapes: *ama1*-D2 (circles), *ama1*-D3 (squares) and *cpmp* (diamonds). Different haplotypes of each marker are represented by the various colours of symbols. A solid line connecting haplotypes represents a persisting haplotype. Dotted lines connect haplotypes that were intermittently undetected, e.g. persisting haplotypes under the detection threshold or reinfection of the same haplotype. Vertical dotted lines represent sampling dates. The colour of these lines indicate whether the sample was *P. falciparum* positive (red) or negative (gray) by qPCR. Red triangle point down represent treatment. Y-axis, within-host haplotype frequency.

Child #3

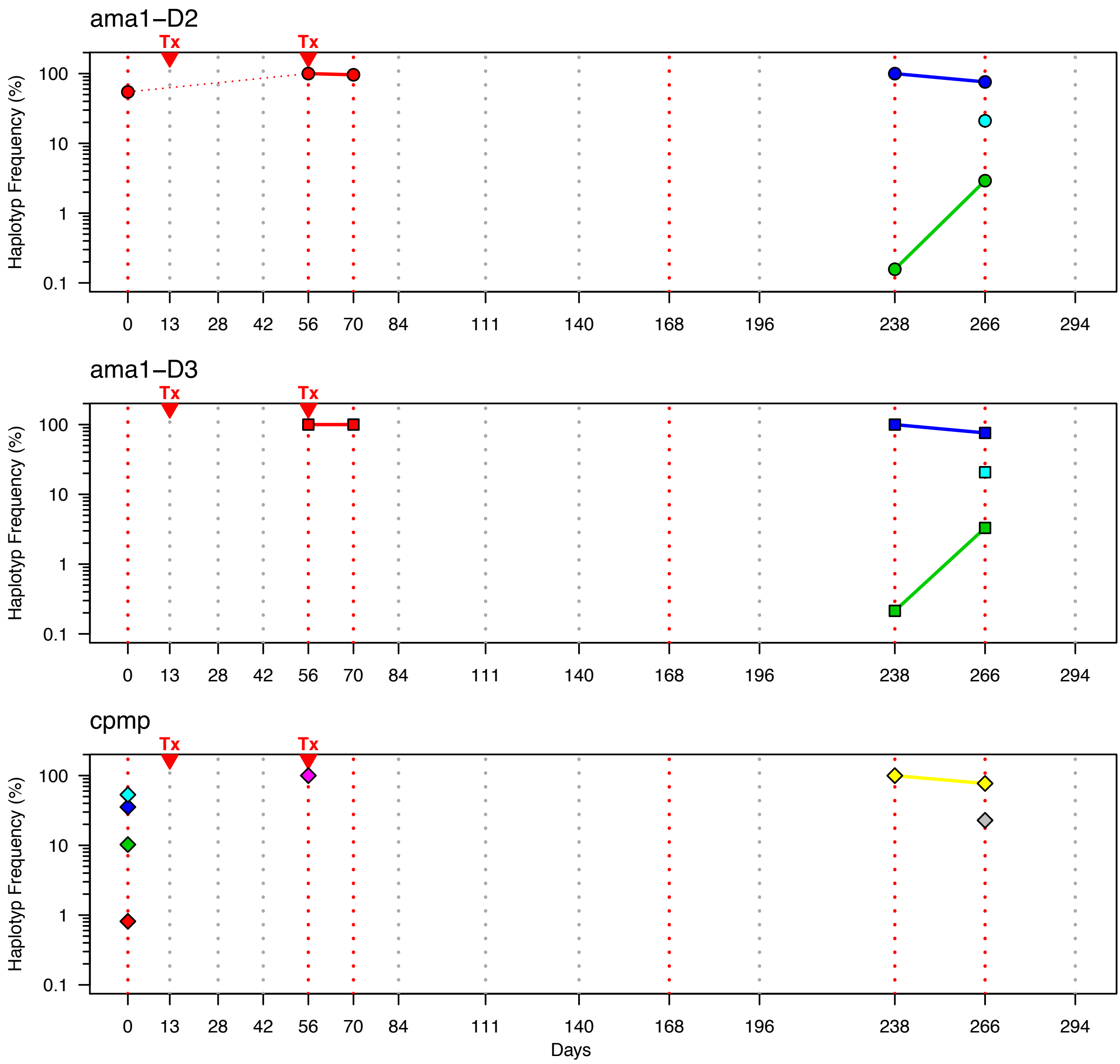

Supplementary Figure S9: Dynamics of within-host haplotype frequencies tracked by Amp-Seq markers in longitudinal samples from 33 children. For each child 3 markers were genotyped, which are distinguished by different shapes: *ama1*-D2 (circles), *ama1*-D3 (squares) and *cpmp* (diamonds). Different haplotypes of each marker are represented by the various colours of symbols. A solid line connecting haplotypes represents a persisting haplotype. Dotted lines connect haplotypes that were intermittently undetected, e.g. persisting haplotypes under the detection threshold or reinfection of the same haplotype. Vertical dotted lines represent sampling dates. The colour of these lines indicate whether the sample was *P. falciparum* positive (red) or negative (gray) by qPCR. Red triangle point down represent treatment. Y-axis, within-host haplotype frequency.

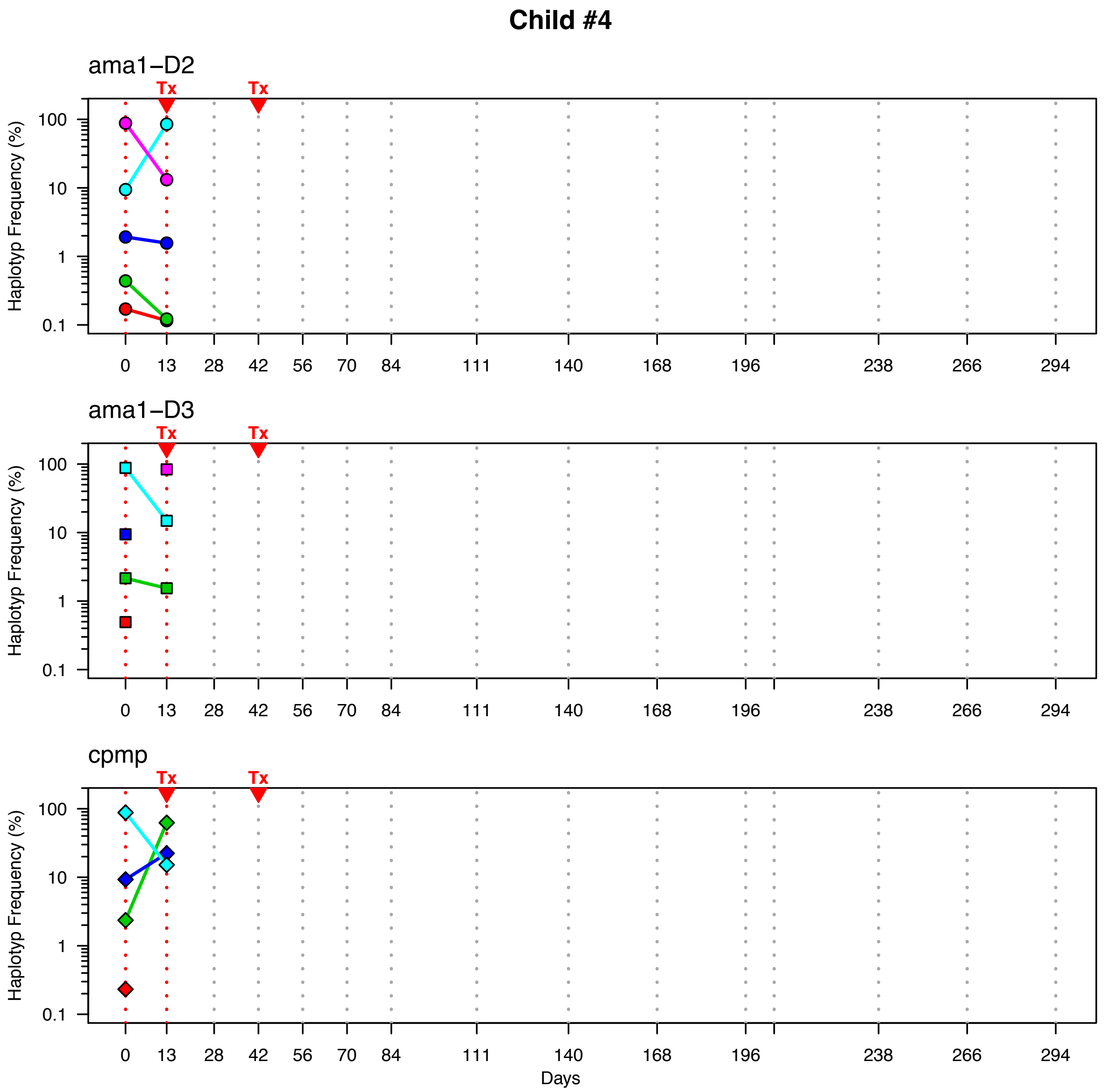

Supplementary Figure S10: Dynamics of within-host haplotype frequencies tracked by Amp-Seq markers in longitudinal samples from 33 children. For each child 3 markers were genotyped, which are distinguished by different shapes: *ama1*-D2 (circles), *ama1*-D3 (squares) and *cpmp* (diamonds). Different haplotypes of each marker are represented by the various colours of symbols. A solid line connecting haplotypes represents a persisting haplotype. Dotted lines connect haplotypes that were intermittently undetected, e.g. persisting haplotypes under the detection threshold or reinfection of the same haplotype. Vertical dotted lines represent sampling dates. The colour of these lines indicate whether the sample was *P. falciparum* positive (red) or negative (gray) by qPCR. Red triangle point down represent treatment. Y-axis, within-host haplotype frequency.

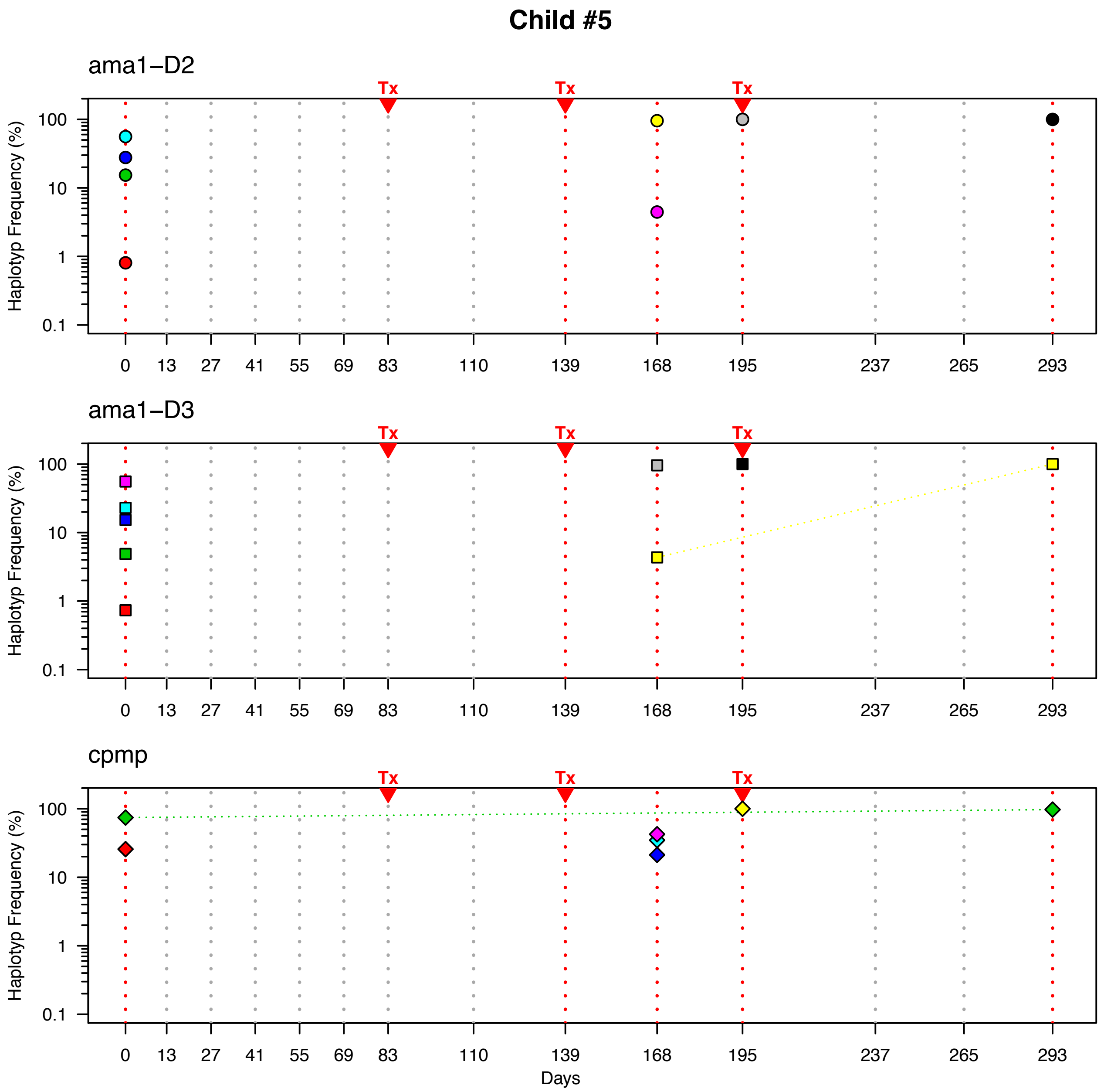

Supplementary Figure S11: Dynamics of within-host haplotype frequencies tracked by Amp-Seq markers in longitudinal samples from 33 children. For each child 3 markers were genotyped, which are distinguished by different shapes: *ama1*-D2 (circles), *ama1*-D3 (squares) and *cpmp* (diamonds). Different haplotypes of each marker are represented by the various colours of symbols. A solid line connecting haplotypes represents a persisting haplotype. Dotted lines connect haplotypes that were intermittently undetected, e.g. persisting haplotypes under the detection threshold or reinfection of the same haplotype. Vertical dotted lines represent sampling dates. The colour of these lines indicate whether the sample was *P. falciparum* positive (red) or negative (gray) by qPCR. Red triangle point down represent treatment. Y-axis, within-host haplotype frequency.

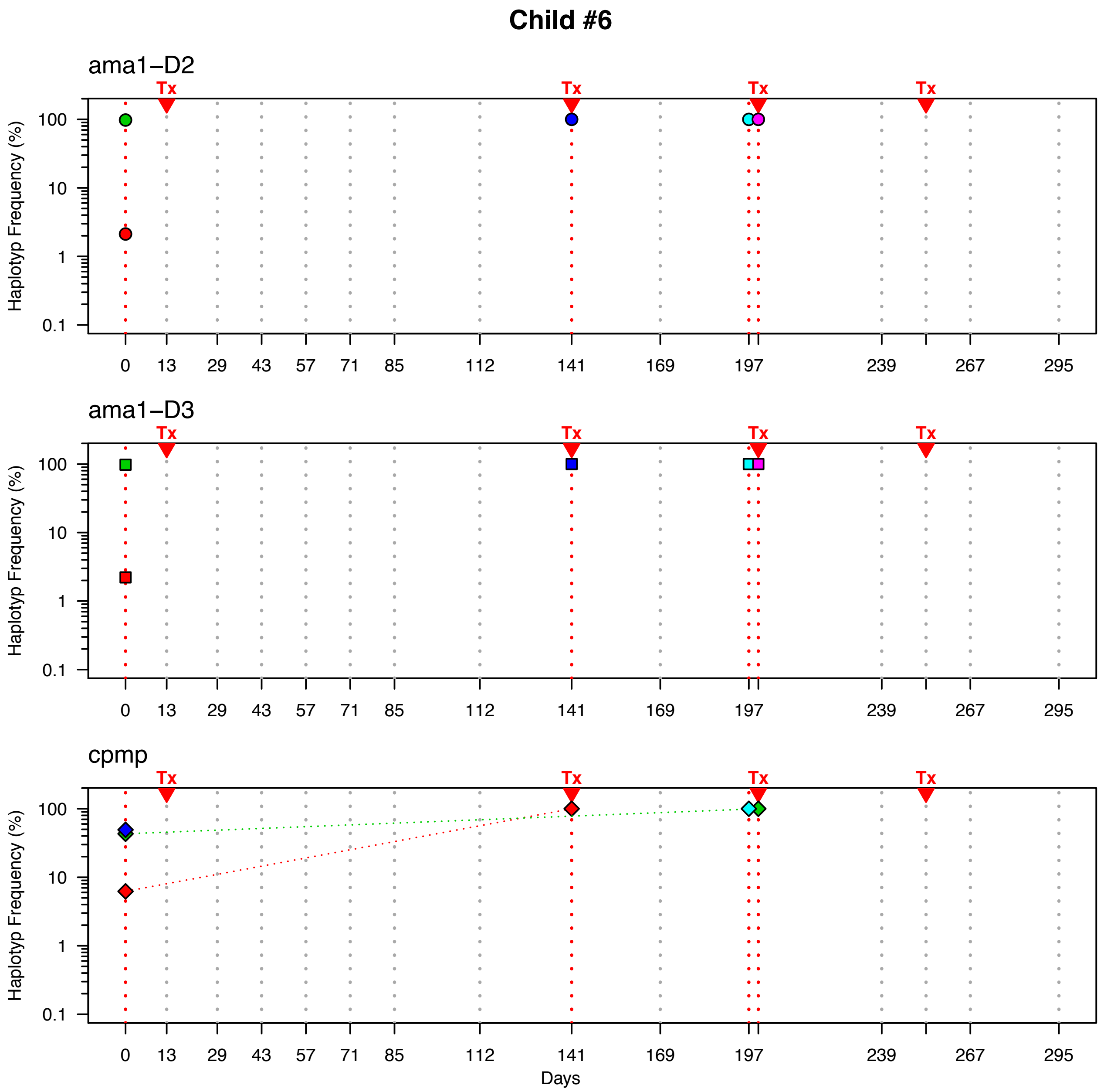

Supplementary Figure S12: Dynamics of within-host haplotype frequencies tracked by Amp-Seq markers in longitudinal samples from 33 children. For each child 3 markers were genotyped, which are distinguished by different shapes: *ama1*-D2 (circles), *ama1*-D3 (squares) and *cpmp* (diamonds). Different haplotypes of each marker are represented by the various colours of symbols. A solid line connecting haplotypes represents a persisting haplotype. Dotted lines connect haplotypes that were intermittently undetected, e.g. persisting haplotypes under the detection threshold or reinfection of the same haplotype. Vertical dotted lines represent sampling dates. The colour of these lines indicate whether the sample was *P. falciparum* positive (red) or negative (gray) by qPCR. Red triangle point down represent treatment. Y-axis, within-host haplotype frequency.

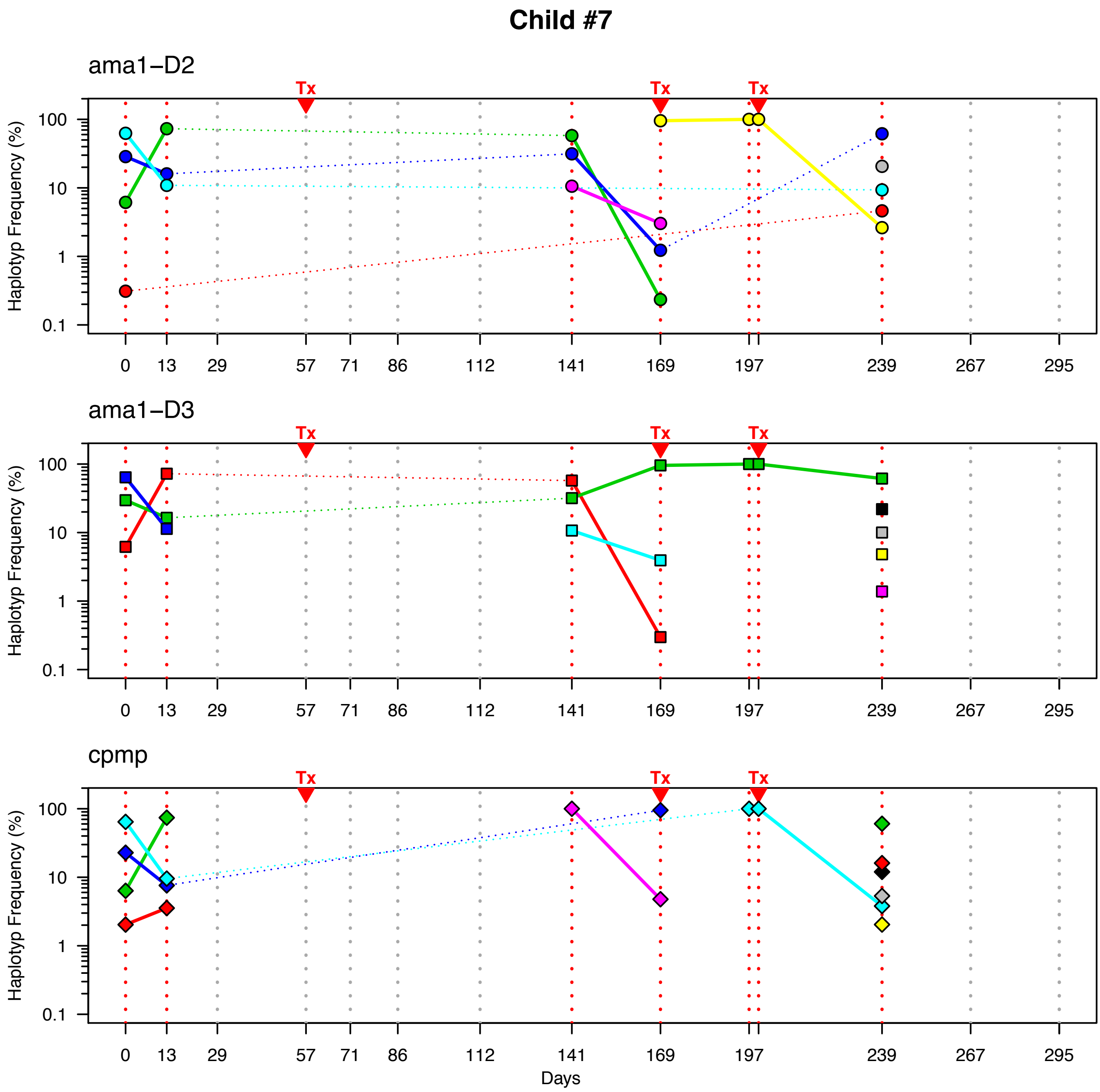

Supplementary Figure S13: Dynamics of within-host haplotype frequencies tracked by Amp-Seq markers in longitudinal samples from 33 children. For each child 3 markers were genotyped, which are distinguished by different shapes: *ama1*-D2 (circles), *ama1*-D3 (squares) and *cpmp* (diamonds). Different haplotypes of each marker are represented by the various colours of symbols. A solid line connecting haplotypes represents a persisting haplotype. Dotted lines connect haplotypes that were intermittently undetected, e.g. persisting haplotypes under the detection threshold or reinfection of the same haplotype. Vertical dotted lines represent sampling dates. The colour of these lines indicate whether the sample was *P. falciparum* positive (red) or negative (gray) by qPCR. Red triangle point down represent treatment. Y-axis, within-host haplotype frequency.

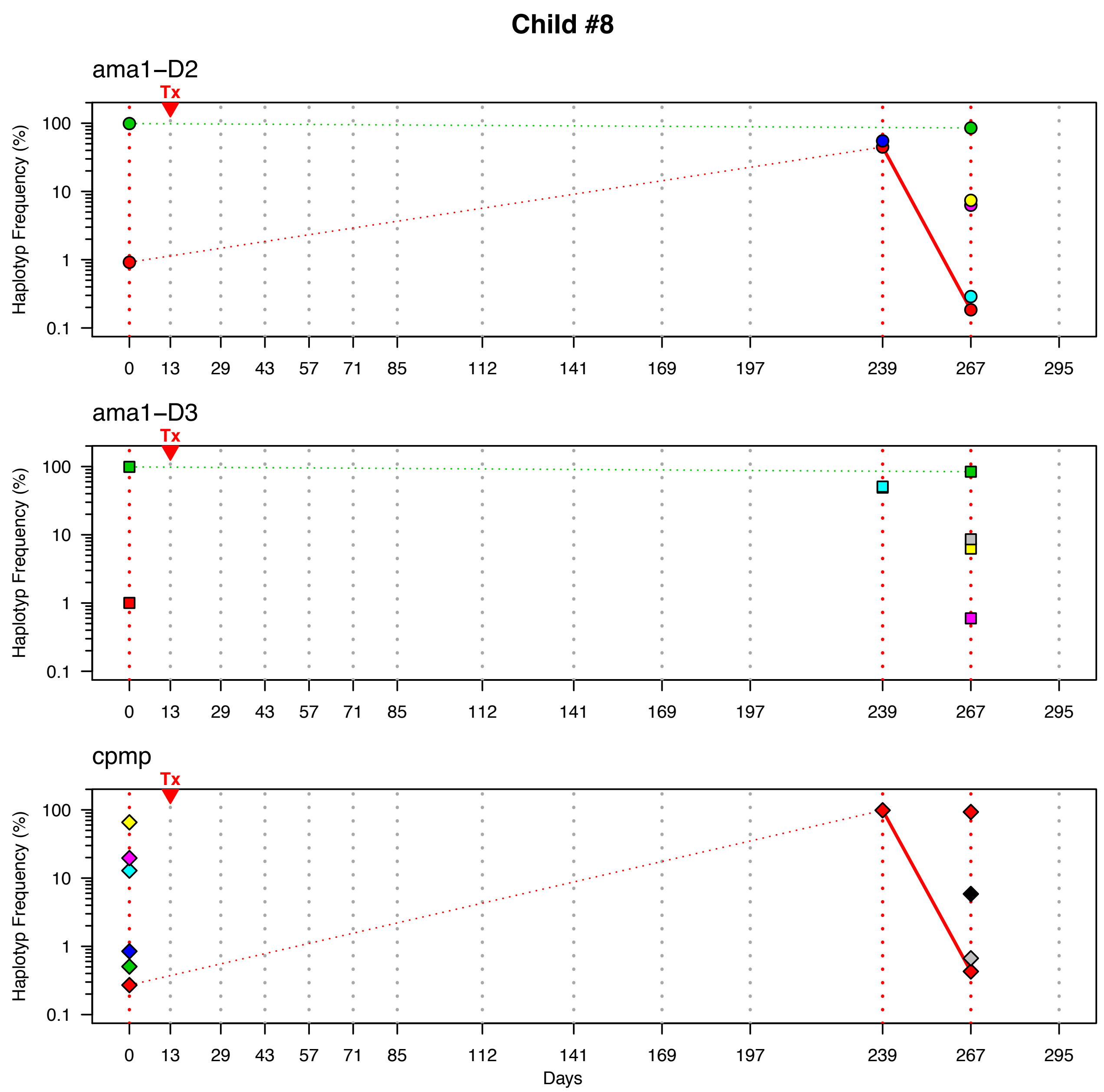

Supplementary Figure S14: Dynamics of within-host haplotype frequencies tracked by Amp-Seq markers in longitudinal samples from 33 children. For each child 3 markers were genotyped, which are distinguished by different shapes: *ama1*-D2 (circles), *ama1*-D3 (squares) and *cpmp* (diamonds). Different haplotypes of each marker are represented by the various colours of symbols. A solid line connecting haplotypes represents a persisting haplotype. Dotted lines connect haplotypes that were intermittently undetected, e.g. persisting haplotypes under the detection threshold or reinfection of the same haplotype. Vertical dotted lines represent sampling dates. The colour of these lines indicate whether the sample was *P. falciparum* positive (red) or negative (gray) by qPCR. Red triangle point down represent treatment. Y-axis, within-host haplotype frequency.

Child #9

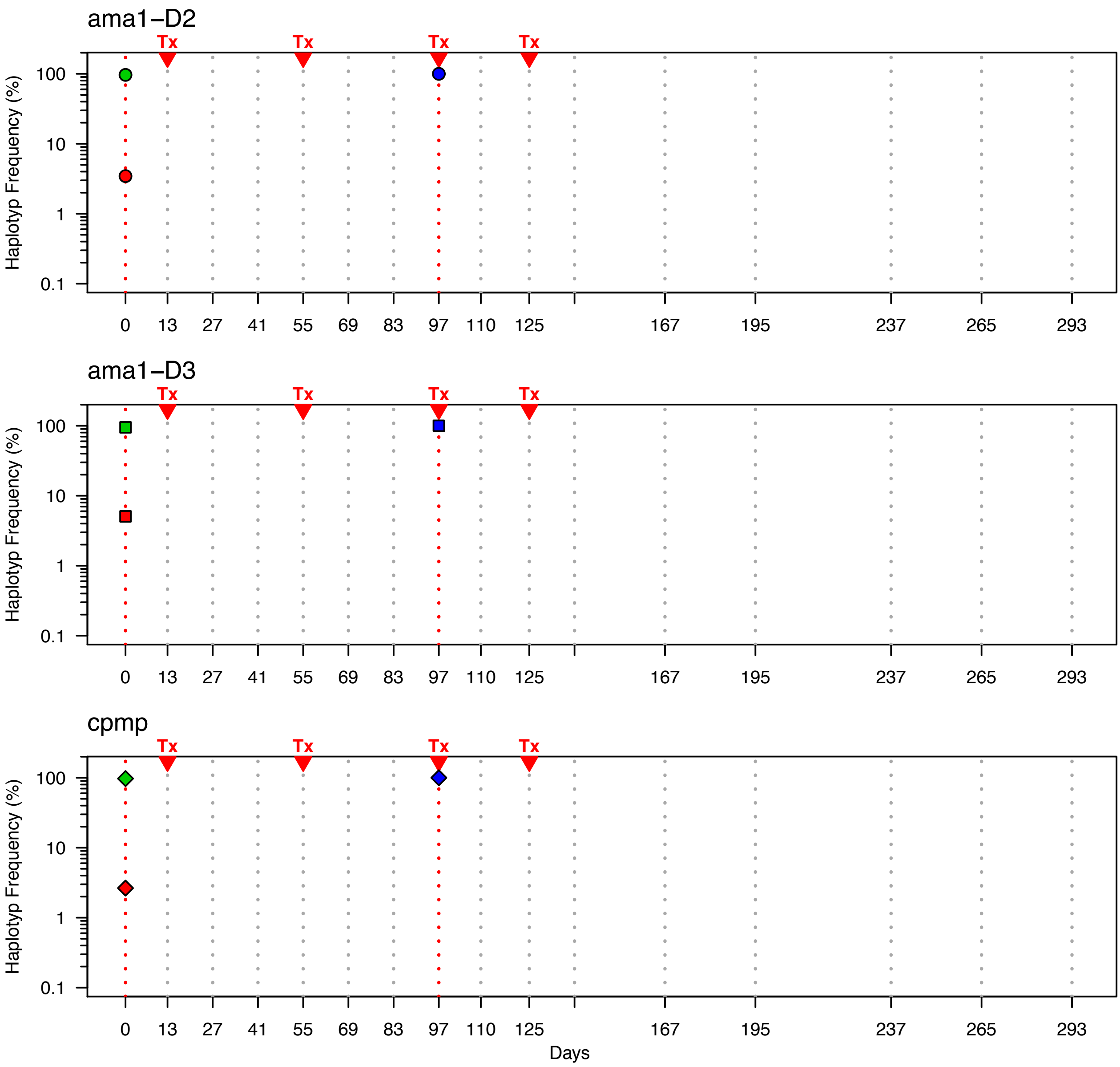

Supplementary Figure S15: Dynamics of within-host haplotype frequencies tracked by Amp-Seq markers in longitudinal samples from 33 children. For each child 3 markers were genotyped, which are distinguished by different shapes: *ama1*-D2 (circles), *ama1*-D3 (squares) and *cpmp* (diamonds). Different haplotypes of each marker are represented by the various colours of symbols. A solid line connecting haplotypes represents a persisting haplotype. Dotted lines connect haplotypes that were intermittently undetected, e.g. persisting haplotypes under the detection threshold or reinfection of the same haplotype. Vertical dotted lines represent sampling dates. The colour of these lines indicate whether the sample was *P. falciparum* positive (red) or negative (gray) by qPCR. Red triangle point down represent treatment. Y-axis, within-host haplotype frequency.

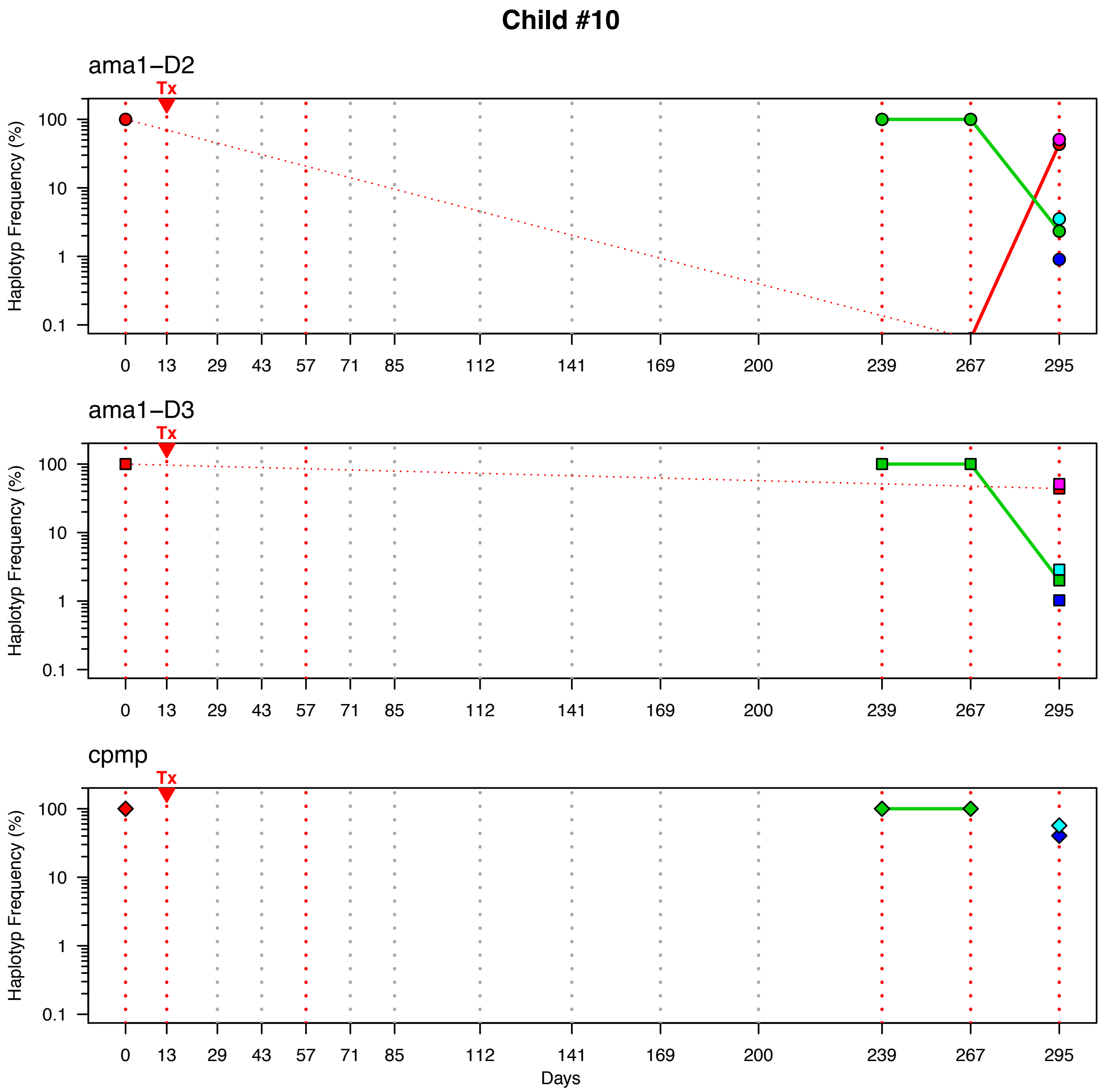

Supplementary Figure S16: Dynamics of within-host haplotype frequencies tracked by Amp-Seq markers in longitudinal samples from 33 children. For each child 3 markers were genotyped, which are distinguished by different shapes: *ama1*-D2 (circles), *ama1*-D3 (squares) and *cpmp* (diamonds). Different haplotypes of each marker are represented by the various colours of symbols. A solid line connecting haplotypes represents a persisting haplotype. Dotted lines connect haplotypes that were intermittently undetected, e.g. persisting haplotypes under the detection threshold or reinfection of the same haplotype. Vertical dotted lines represent sampling dates. The colour of these lines indicate whether the sample was *P. falciparum* positive (red) or negative (gray) by qPCR. Red triangle point down represent treatment. Y-axis, within-host haplotype frequency.

Child #11

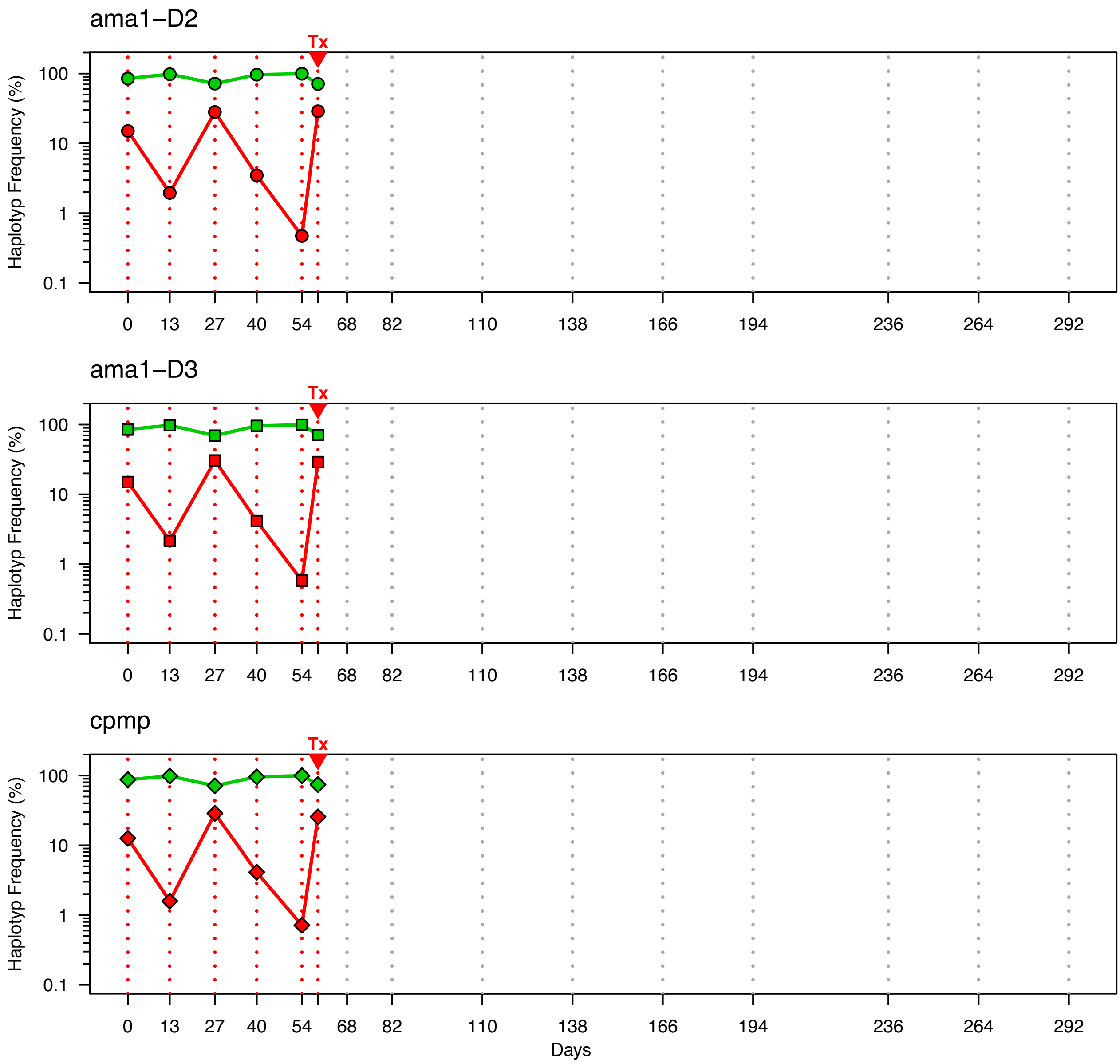

Supplementary Figure S17: Dynamics of within-host haplotype frequencies tracked by Amp-Seq markers in longitudinal samples from 33 children. For each child 3 markers were genotyped, which are distinguished by different shapes: *ama1*-D2 (circles), *ama1*-D3 (squares) and *cpmp* (diamonds). Different haplotypes of each marker are represented by the various colours of symbols. A solid line connecting haplotypes represents a persisting haplotype. Dotted lines connect haplotypes that were intermittently undetected, e.g. persisting haplotypes under the detection threshold or reinfection of the same haplotype. Vertical dotted lines represent sampling dates. The colour of these lines indicate whether the sample was *P. falciparum* positive (red) or negative (gray) by qPCR. Red triangle point down represent treatment. Y-axis, within-host haplotype frequency.

Child #12

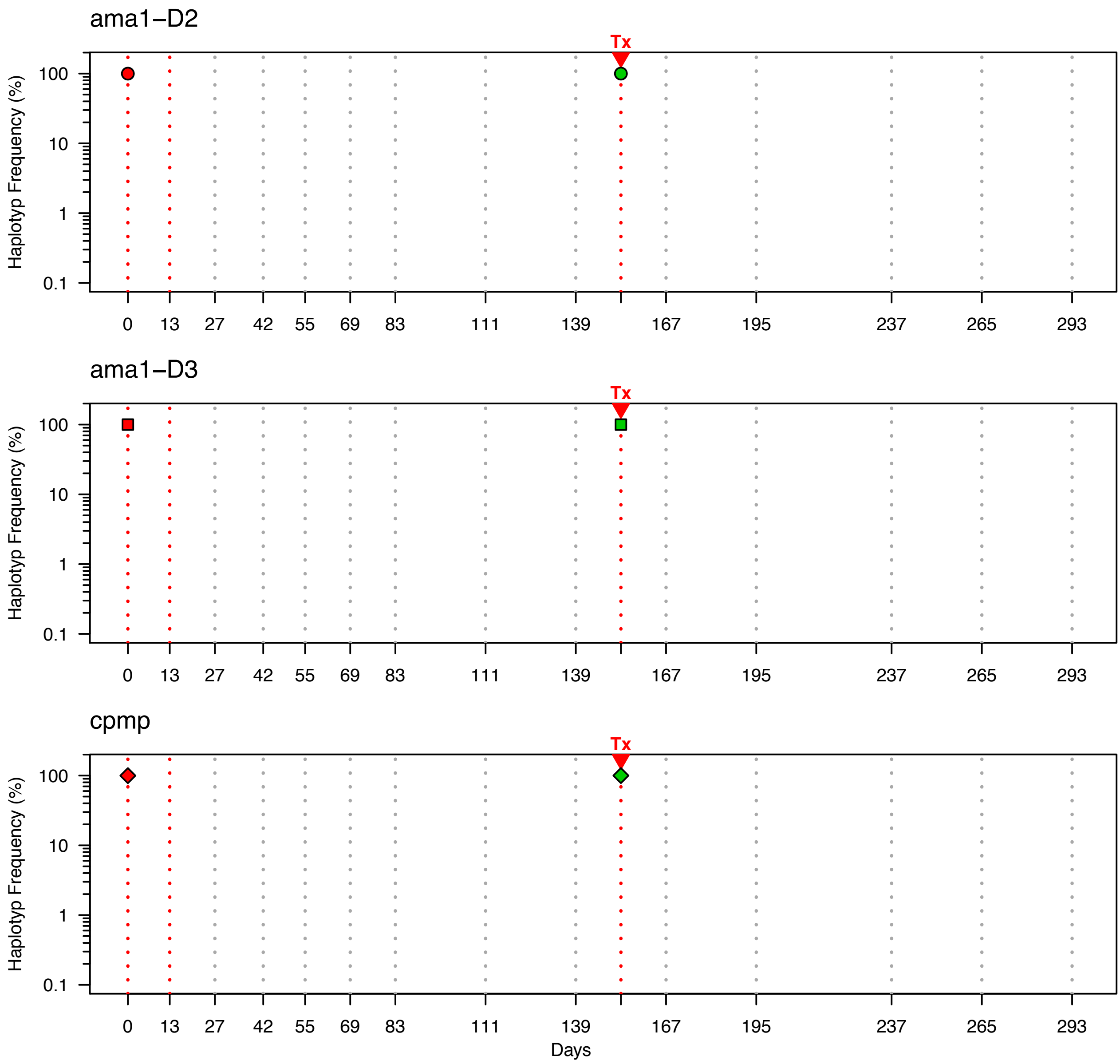

Supplementary Figure S18: Dynamics of within-host haplotype frequencies tracked by Amp-Seq markers in longitudinal samples from 33 children. For each child 3 markers were genotyped, which are distinguished by different shapes: *ama1*-D2 (circles), *ama1*-D3 (squares) and *cpmp* (diamonds). Different haplotypes of each marker are represented by the various colours of symbols. A solid line connecting haplotypes represents a persisting haplotype. Dotted lines connect haplotypes that were intermittently undetected, e.g. persisting haplotypes under the detection threshold or reinfection of the same haplotype. Vertical dotted lines represent sampling dates. The colour of these lines indicate whether the sample was *P. falciparum* positive (red) or negative (gray) by qPCR. Red triangle point down represent treatment. Y-axis, within-host haplotype frequency.

Child #13

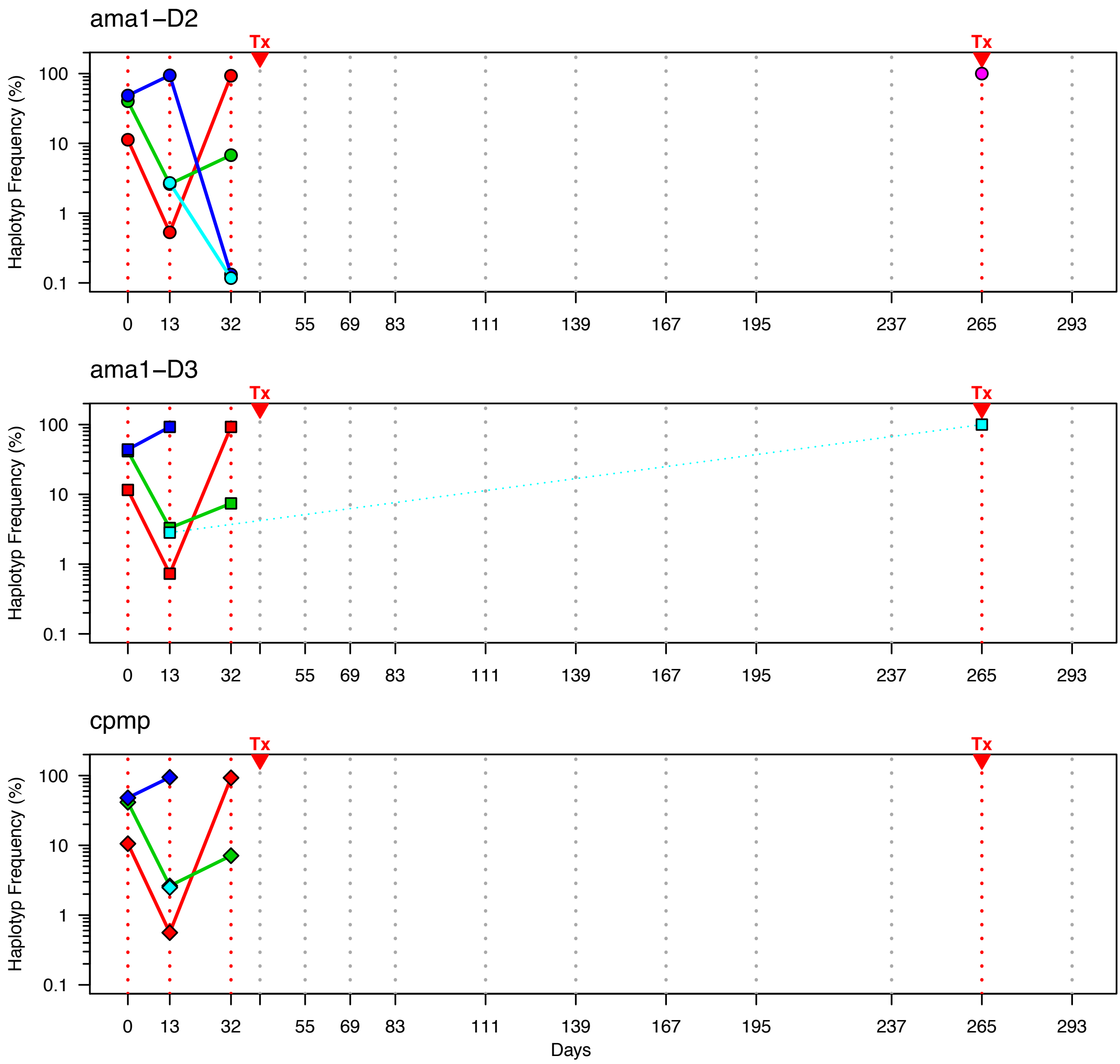

Supplementary Figure S19: Dynamics of within-host haplotype frequencies tracked by Amp-Seq markers in longitudinal samples from 33 children. For each child 3 markers were genotyped, which are distinguished by different shapes: *ama1*-D2 (circles), *ama1*-D3 (squares) and *cpmp* (diamonds). Different haplotypes of each marker are represented by the various colours of symbols. A solid line connecting haplotypes represents a persisting haplotype. Dotted lines connect haplotypes that were intermittently undetected, e.g. persisting haplotypes under the detection threshold or reinfection of the same haplotype. Vertical dotted lines represent sampling dates. The colour of these lines indicate whether the sample was *P. falciparum* positive (red) or negative (gray) by qPCR. Red triangle point down represent treatment. Y-axis, within-host haplotype frequency.

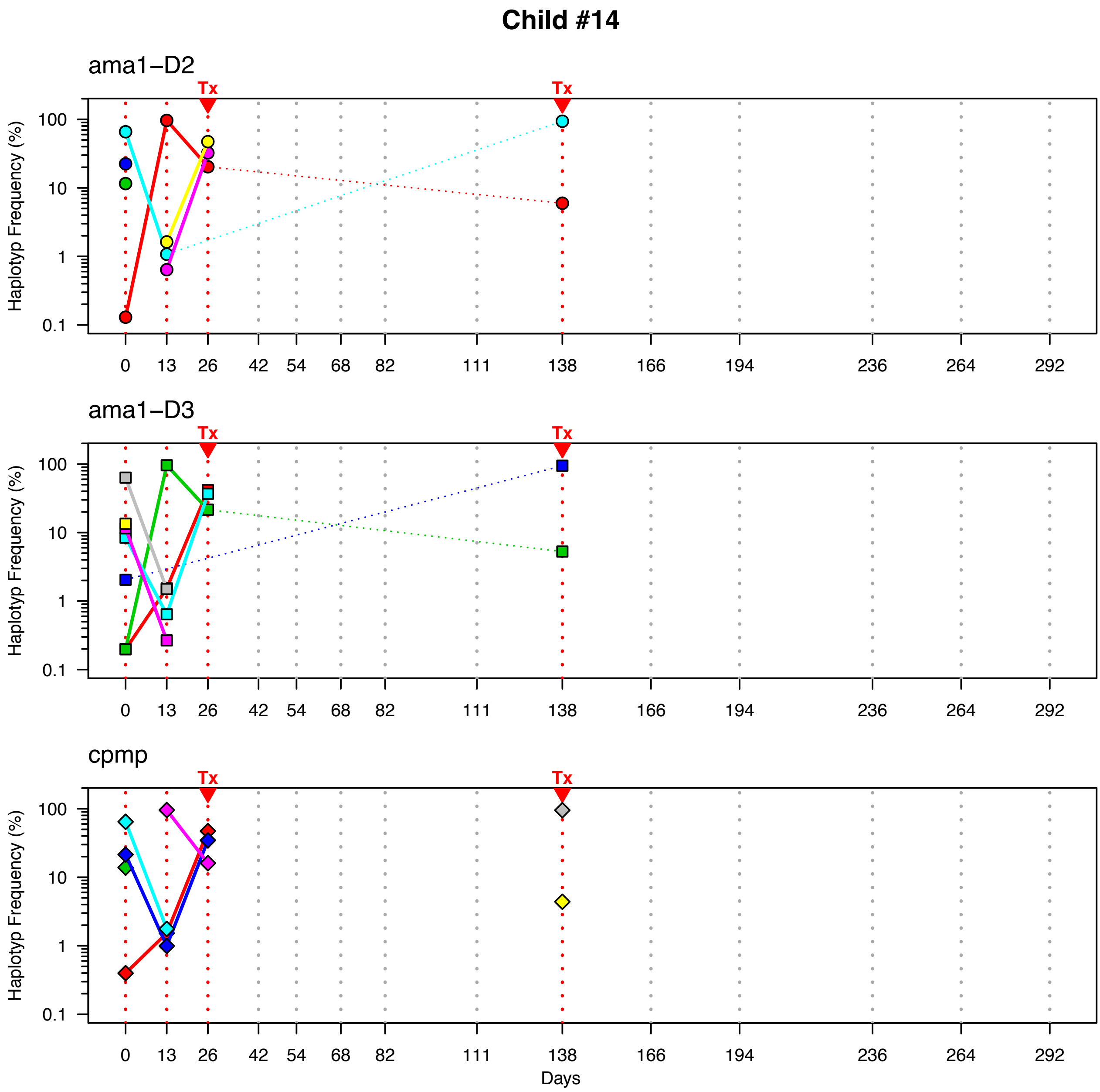

Supplementary Figure S20: Dynamics of within-host haplotype frequencies tracked by Amp-Seq markers in longitudinal samples from 33 children. For each child 3 markers were genotyped, which are distinguished by different shapes: *ama1*-D2 (circles), *ama1*-D3 (squares) and *cpmp* (diamonds). Different haplotypes of each marker are represented by the various colours of symbols. A solid line connecting haplotypes represents a persisting haplotype. Dotted lines connect haplotypes that were intermittently undetected, e.g. persisting haplotypes under the detection threshold or reinfection of the same haplotype. Vertical dotted lines represent sampling dates. The colour of these lines indicate whether the sample was *P. falciparum* positive (red) or negative (gray) by qPCR. Red triangle point down represent treatment. Y-axis, within-host haplotype frequency.

Child #15

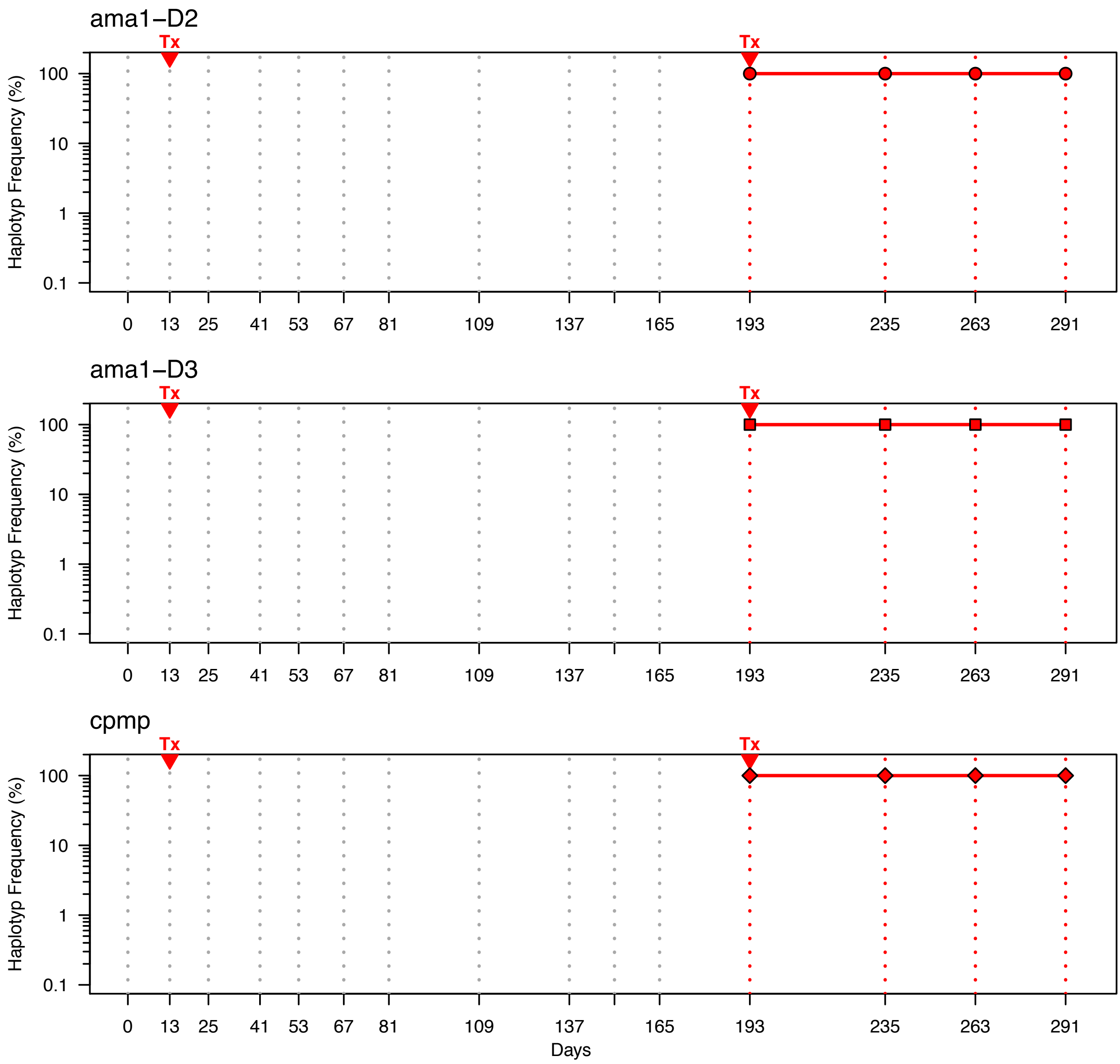

Supplementary Figure S21: Dynamics of within-host haplotype frequencies tracked by Amp-Seq markers in longitudinal samples from 33 children. For each child 3 markers were genotyped, which are distinguished by different shapes: *ama1*-D2 (circles), *ama1*-D3 (squares) and *cpmp* (diamonds). Different haplotypes of each marker are represented by the various colours of symbols. A solid line connecting haplotypes represents a persisting haplotype. Dotted lines connect haplotypes that were intermittently undetected, e.g. persisting haplotypes under the detection threshold or reinfection of the same haplotype. Vertical dotted lines represent sampling dates. The colour of these lines indicate whether the sample was *P. falciparum* positive (red) or negative (gray) by qPCR. Red triangle point down represent treatment. Y-axis, within-host haplotype frequency.

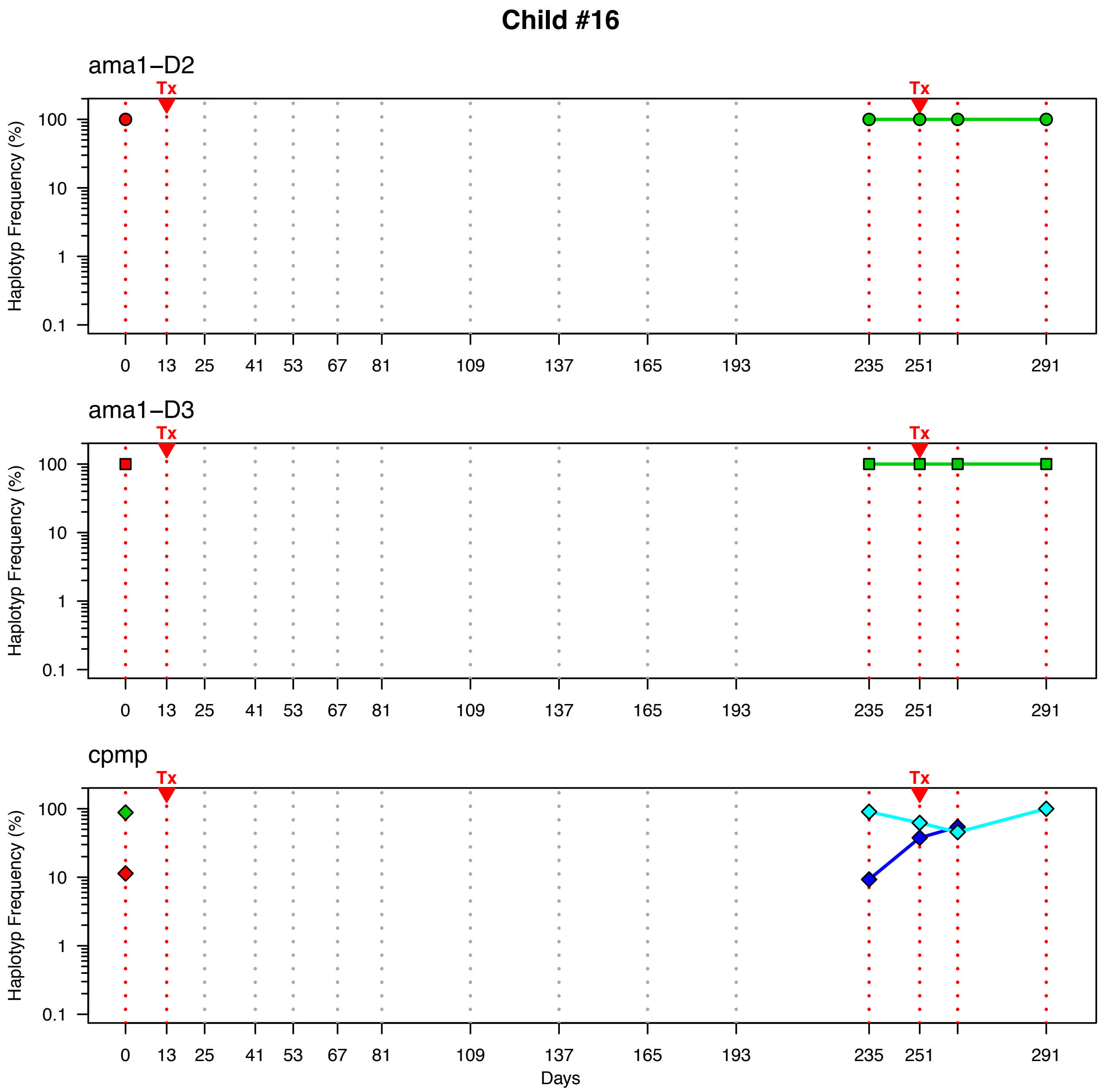

Supplementary Figure S22: Dynamics of within-host haplotype frequencies tracked by Amp-Seq markers in longitudinal samples from 33 children. For each child 3 markers were genotyped, which are distinguished by different shapes: *ama1*-D2 (circles), *ama1*-D3 (squares) and *cpmp* (diamonds). Different haplotypes of each marker are represented by the various colours of symbols. A solid line connecting haplotypes represents a persisting haplotype. Dotted lines connect haplotypes that were intermittently undetected, e.g. persisting haplotypes under the detection threshold or reinfection of the same haplotype. Vertical dotted lines represent sampling dates. The colour of these lines indicate whether the sample was *P. falciparum* positive (red) or negative (gray) by qPCR. Red triangle point down represent treatment. Y-axis, within-host haplotype frequency.

Child #17

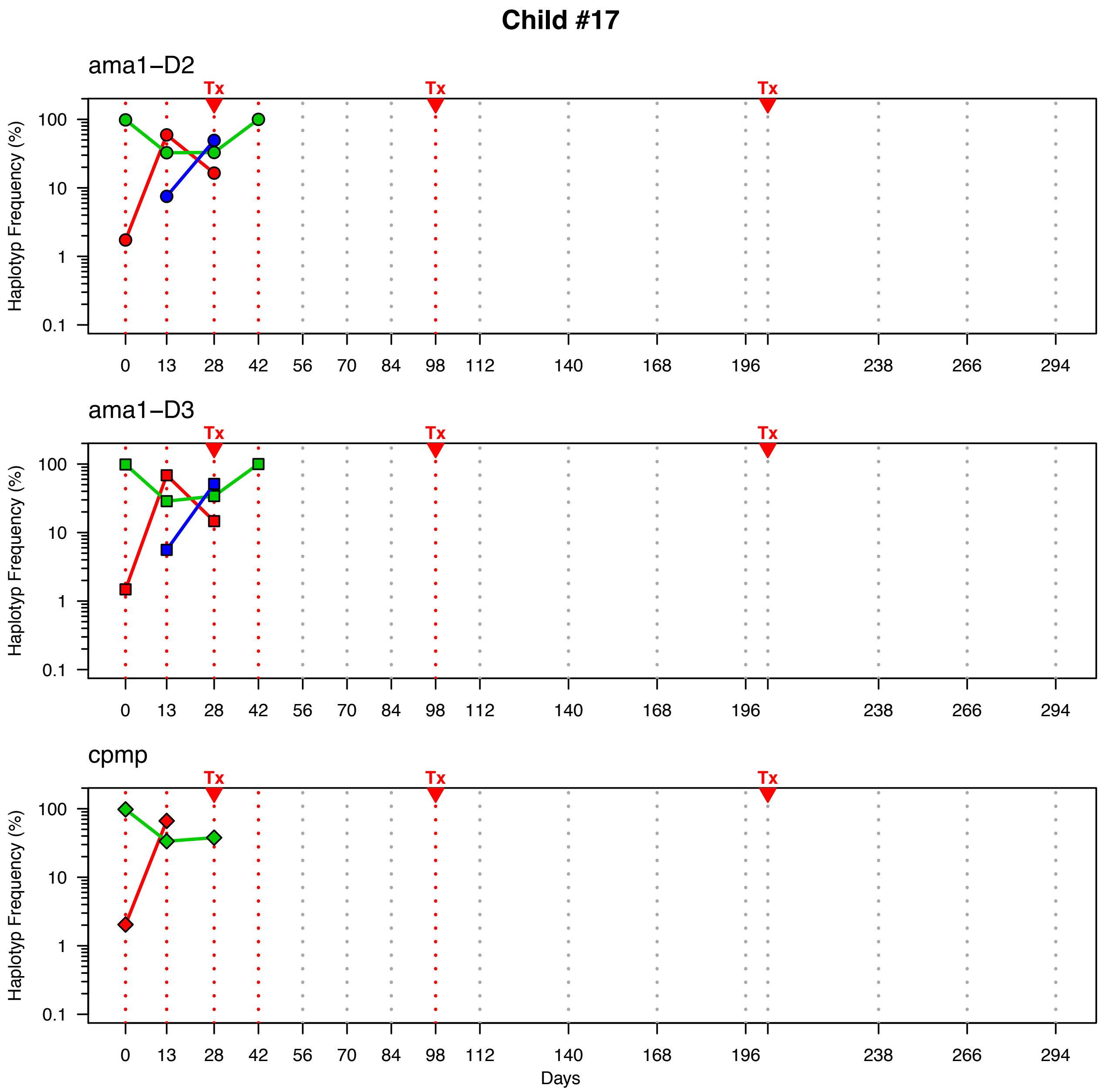

Supplementary Figure S23: Dynamics of within-host haplotype frequencies tracked by Amp-Seq markers in longitudinal samples from 33 children. For each child 3 markers were genotyped, which are distinguished by different shapes: *ama1*-D2 (circles), *ama1*-D3 (squares) and *cpmp* (diamonds). Different haplotypes of each marker are represented by the various colours of symbols. A solid line connecting haplotypes represents a persisting haplotype. Dotted lines connect haplotypes that were intermittently undetected, e.g. persisting haplotypes under the detection threshold or reinfection of the same haplotype. Vertical dotted lines represent sampling dates. The colour of these lines indicate whether the sample was *P. falciparum* positive (red) or negative (gray) by qPCR. Red triangle point down represent treatment. Y-axis, within-host haplotype frequency.

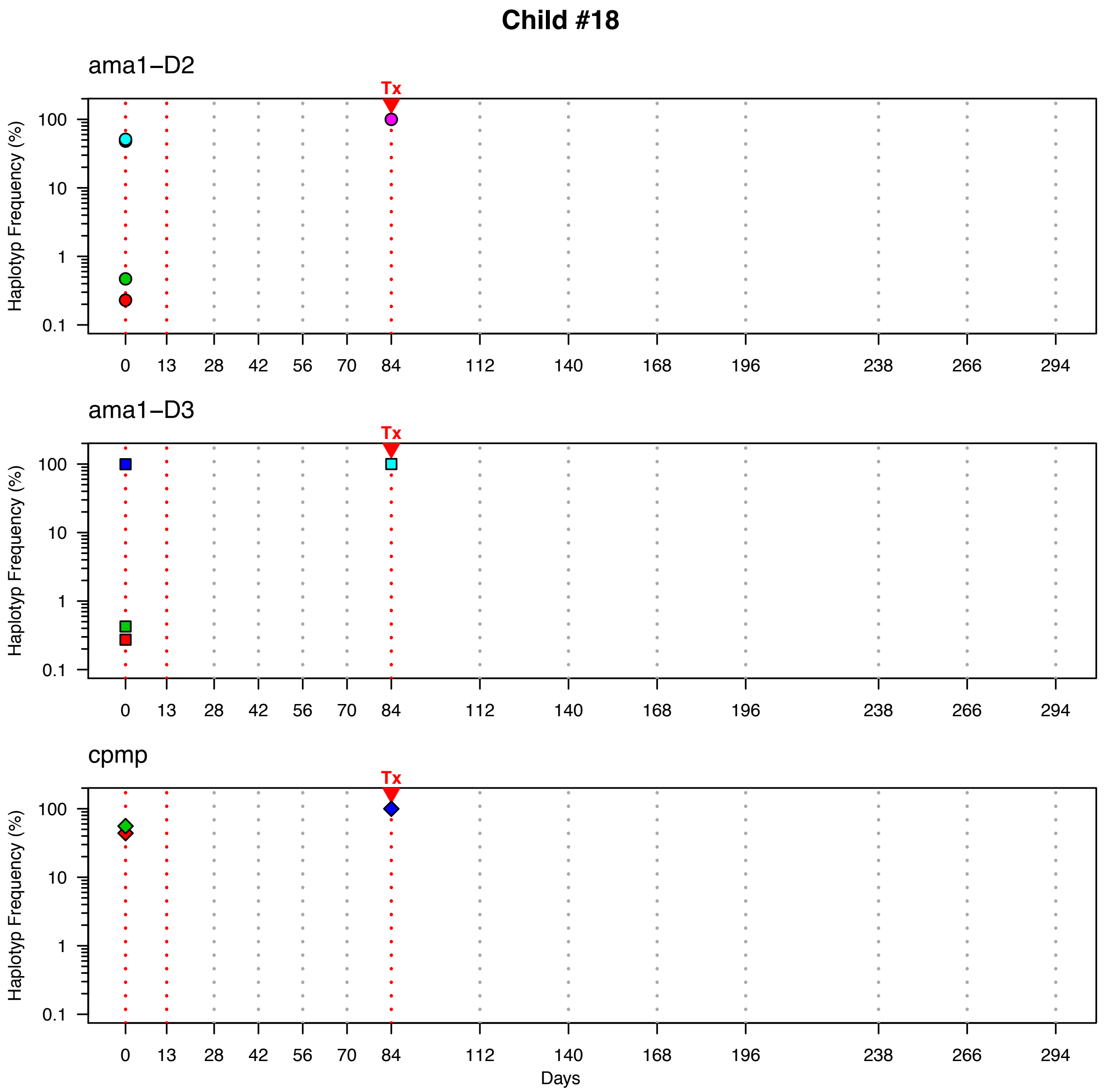

Supplementary Figure S24: Dynamics of within-host haplotype frequencies tracked by Amp-Seq markers in longitudinal samples from 33 children. For each child 3 markers were genotyped, which are distinguished by different shapes: *ama1*-D2 (circles), *ama1*-D3 (squares) and *cpmp* (diamonds). Different haplotypes of each marker are represented by the various colours of symbols. A solid line connecting haplotypes represents a persisting haplotype. Dotted lines connect haplotypes that were intermittently undetected, e.g. persisting haplotypes under the detection threshold or reinfection of the same haplotype. Vertical dotted lines represent sampling dates. The colour of these lines indicate whether the sample was *P. falciparum* positive (red) or negative (gray) by qPCR. Red triangle point down represent treatment. Y-axis, within-host haplotype frequency.

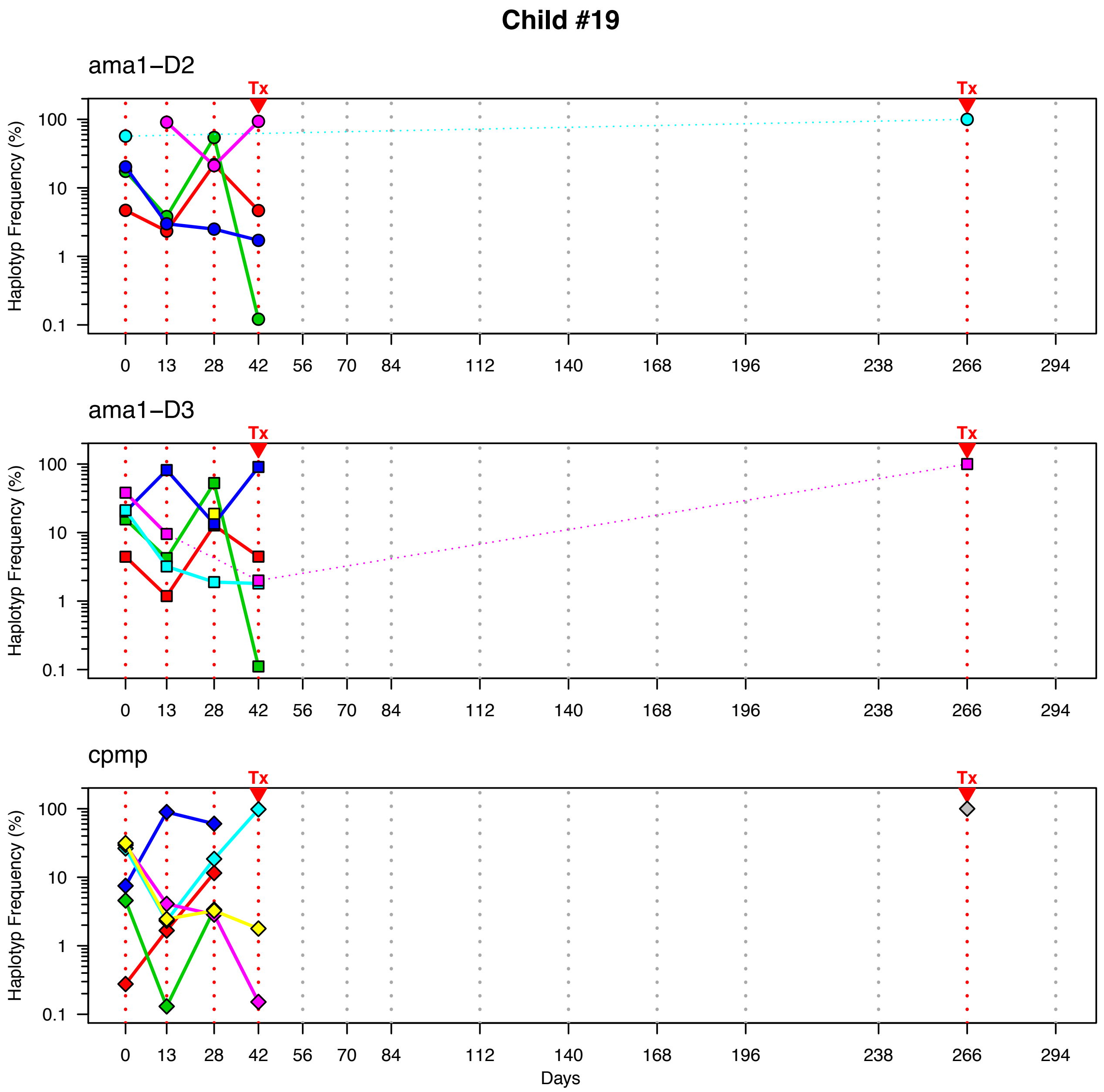

Supplementary Figure S25: Dynamics of within-host haplotype frequencies tracked by Amp-Seq markers in longitudinal samples from 33 children. For each child 3 markers were genotyped, which are distinguished by different shapes: *ama1*-D2 (circles), *ama1*-D3 (squares) and *cpmp* (diamonds). Different haplotypes of each marker are represented by the various colours of symbols. A solid line connecting haplotypes represents a persisting haplotype. Dotted lines connect haplotypes that were intermittently undetected, e.g. persisting haplotypes under the detection threshold or reinfection of the same haplotype. Vertical dotted lines represent sampling dates. The colour of these lines indicate whether the sample was *P. falciparum* positive (red) or negative (gray) by qPCR. Red triangle point down represent treatment. Y-axis, within-host haplotype frequency.

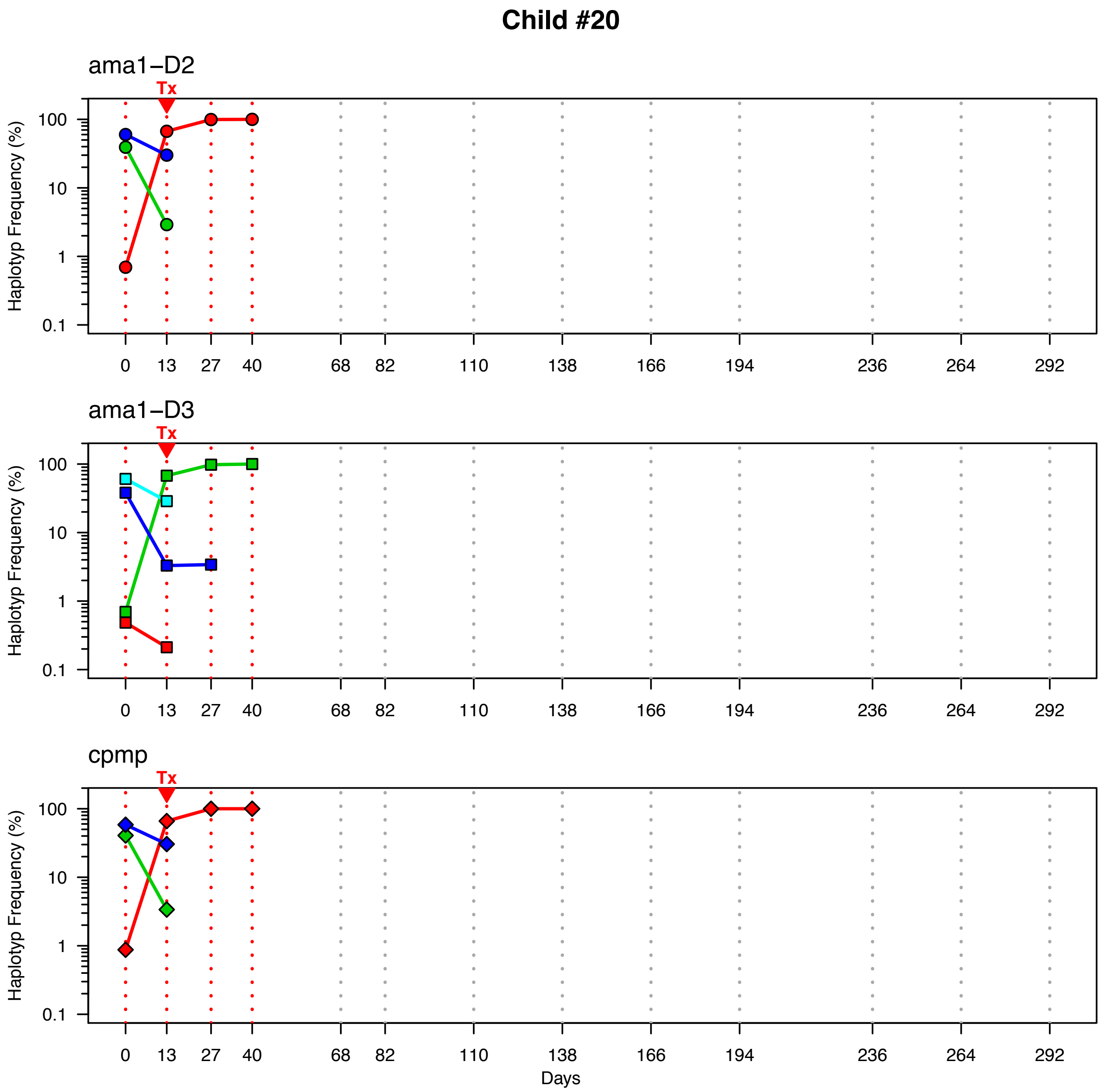

Supplementary Figure S26: Dynamics of within-host haplotype frequencies tracked by Amp-Seq markers in longitudinal samples from 33 children. For each child 3 markers were genotyped, which are distinguished by different shapes: *ama1*-D2 (circles), *ama1*-D3 (squares) and *cpmp* (diamonds). Different haplotypes of each marker are represented by the various colours of symbols. A solid line connecting haplotypes represents a persisting haplotype. Dotted lines connect haplotypes that were intermittently undetected, e.g. persisting haplotypes under the detection threshold or reinfection of the same haplotype. Vertical dotted lines represent sampling dates. The colour of these lines indicate whether the sample was *P. falciparum* positive (red) or negative (gray) by qPCR. Red triangle point down represent treatment. Y-axis, within-host haplotype frequency.

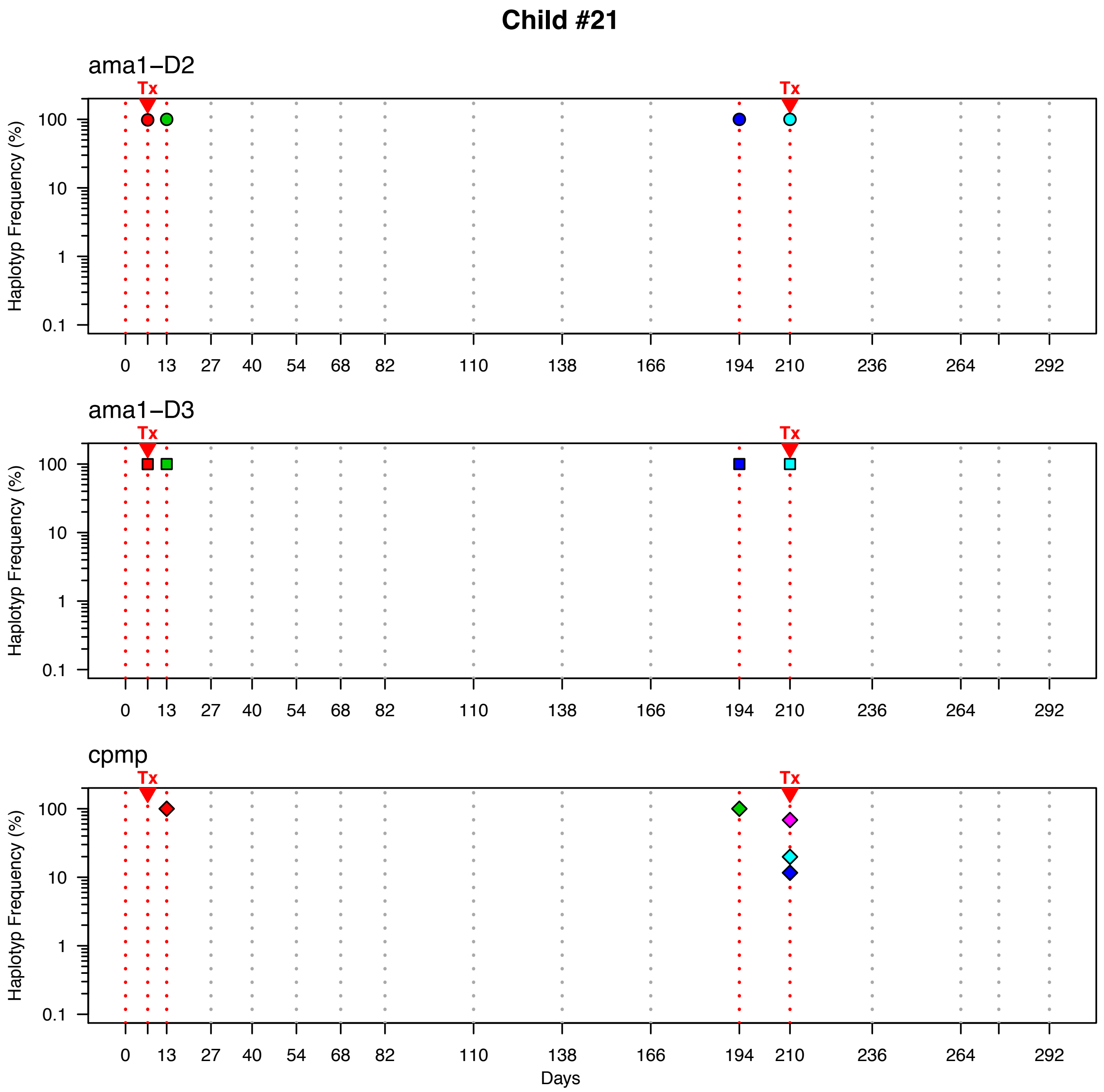

Supplementary Figure S27: Dynamics of within-host haplotype frequencies tracked by Amp-Seq markers in longitudinal samples from 33 children. For each child 3 markers were genotyped, which are distinguished by different shapes: *ama1*-D2 (circles), *ama1*-D3 (squares) and *cpmp* (diamonds). Different haplotypes of each marker are represented by the various colours of symbols. A solid line connecting haplotypes represents a persisting haplotype. Dotted lines connect haplotypes that were intermittently undetected, e.g. persisting haplotypes under the detection threshold or reinfection of the same haplotype. Vertical dotted lines represent sampling dates. The colour of these lines indicate whether the sample was *P. falciparum* positive (red) or negative (gray) by qPCR. Red triangle point down represent treatment. Y-axis, within-host haplotype frequency.

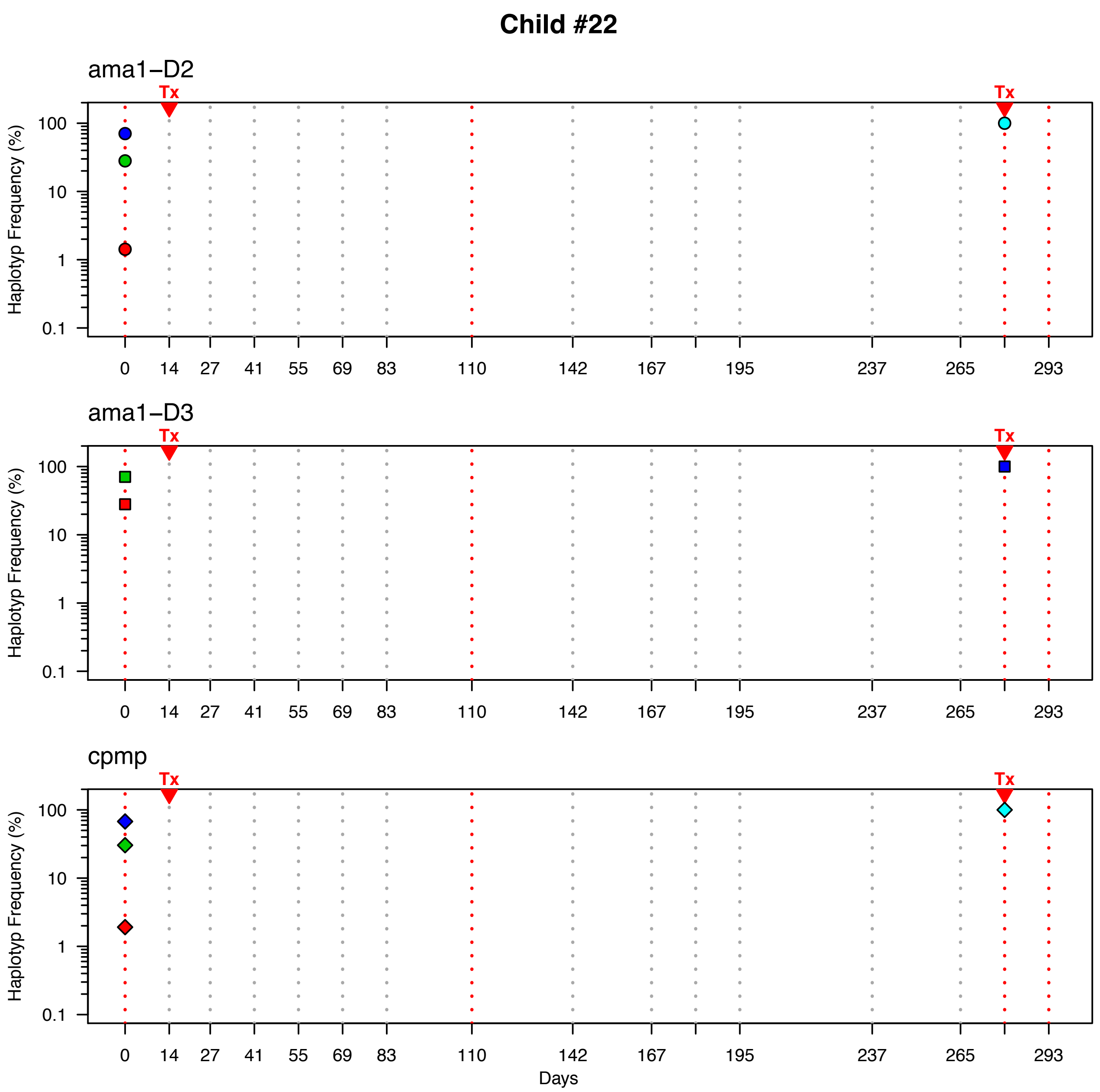

Supplementary Figure S28: Dynamics of within-host haplotype frequencies tracked by Amp-Seq markers in longitudinal samples from 33 children. For each child 3 markers were genotyped, which are distinguished by different shapes: *ama1*-D2 (circles), *ama1*-D3 (squares) and *cpmp* (diamonds). Different haplotypes of each marker are represented by the various colours of symbols. A solid line connecting haplotypes represents a persisting haplotype. Dotted lines connect haplotypes that were intermittently undetected, e.g. persisting haplotypes under the detection threshold or reinfection of the same haplotype. Vertical dotted lines represent sampling dates. The colour of these lines indicate whether the sample was *P. falciparum* positive (red) or negative (gray) by qPCR. Red triangle point down represent treatment. Y-axis, within-host haplotype frequency.

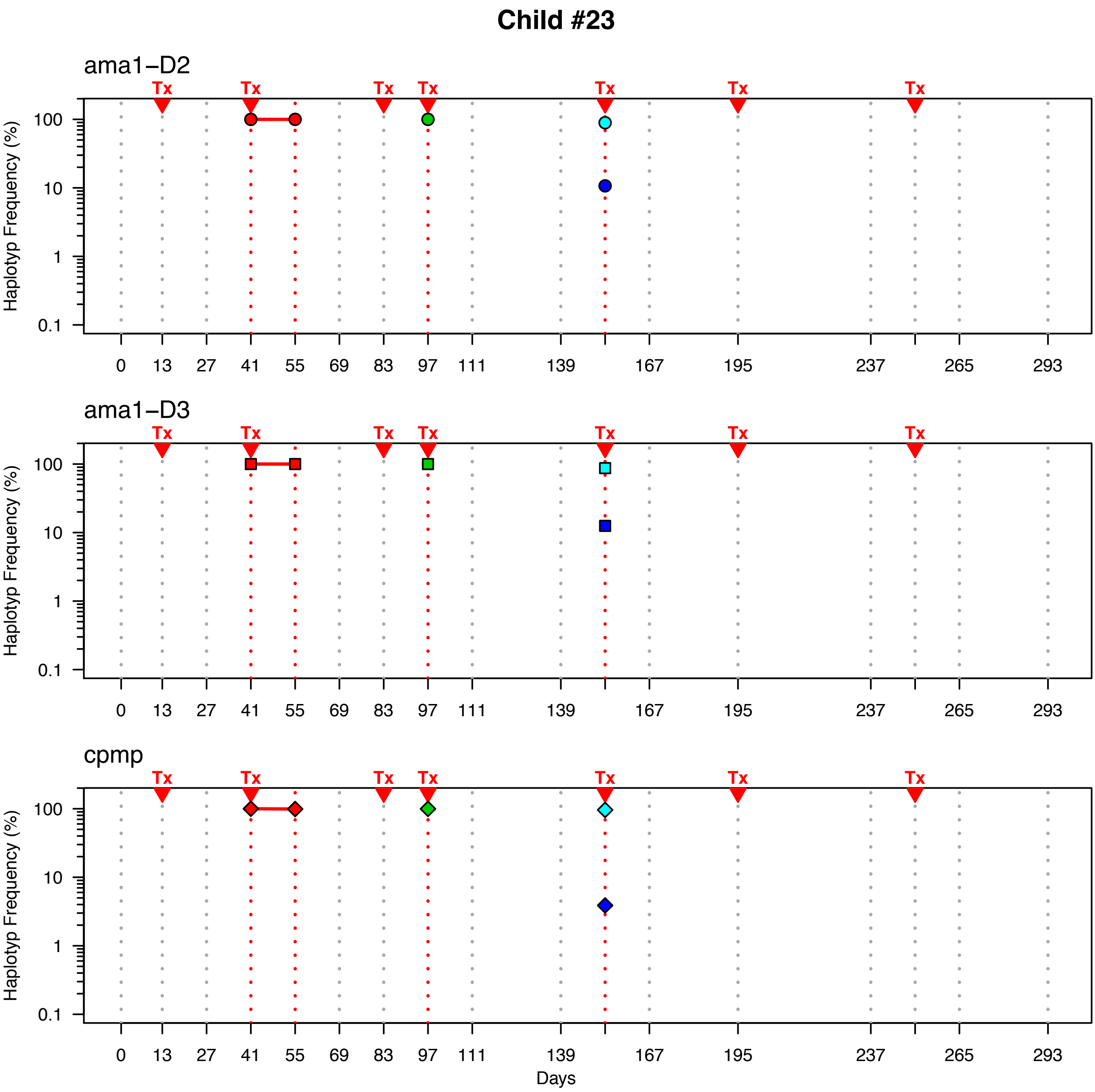

Supplementary Figure S29: Dynamics of within-host haplotype frequencies tracked by Amp-Seq markers in longitudinal samples from 33 children. For each child 3 markers were genotyped, which are distinguished by different shapes: *ama1*-D2 (circles), *ama1*-D3 (squares) and *cpmp* (diamonds). Different haplotypes of each marker are represented by the various colours of symbols. A solid line connecting haplotypes represents a persisting haplotype. Dotted lines connect haplotypes that were intermittently undetected, e.g. persisting haplotypes under the detection threshold or reinfection of the same haplotype. Vertical dotted lines represent sampling dates. The colour of these lines indicate whether the sample was *P. falciparum* positive (red) or negative (gray) by qPCR. Red triangle point down represent treatment. Y-axis, within-host haplotype frequency.

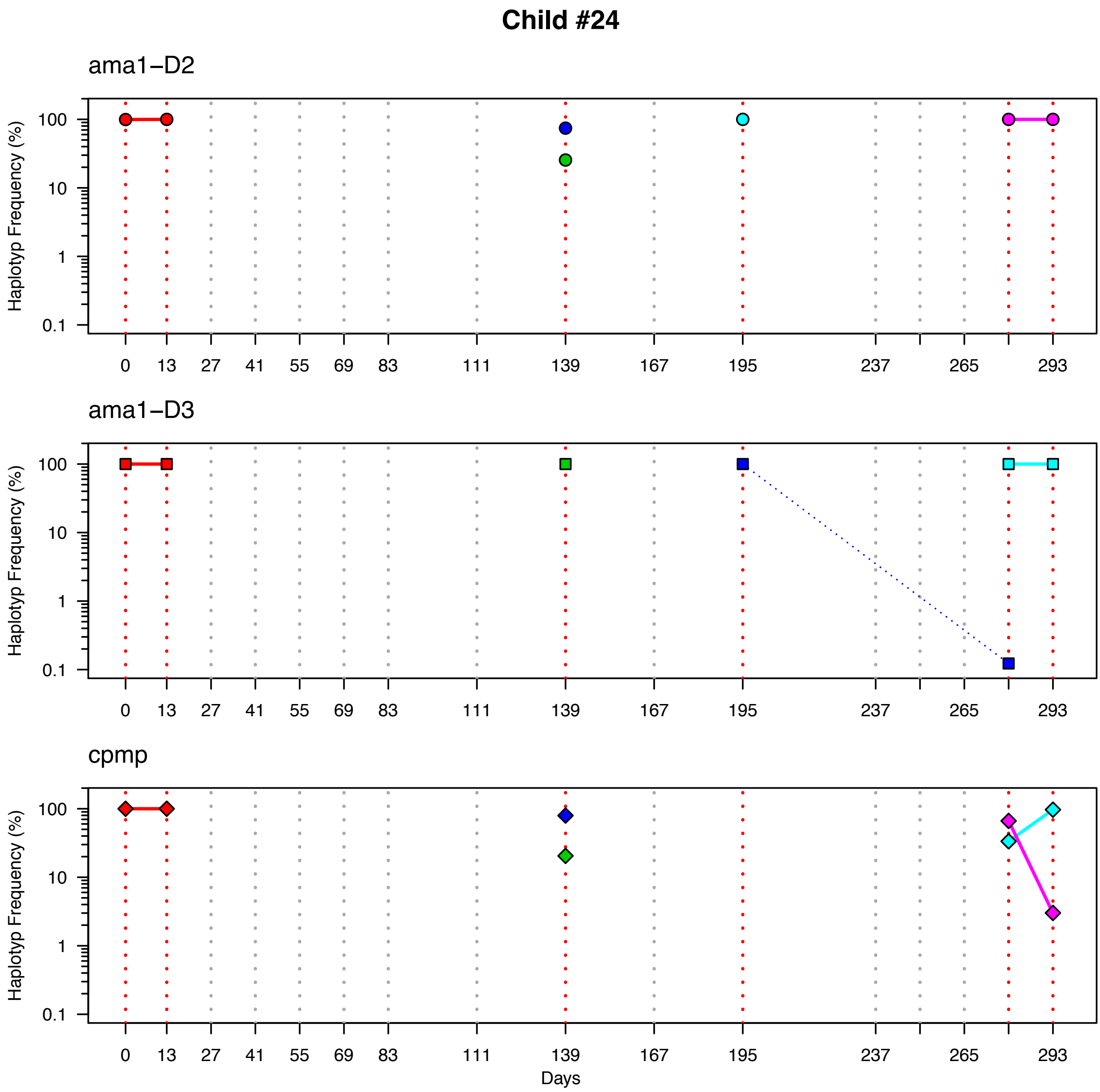

Supplementary Figure S30: Dynamics of within-host haplotype frequencies tracked by Amp-Seq markers in longitudinal samples from 33 children. For each child 3 markers were genotyped, which are distinguished by different shapes: *ama1*-D2 (circles), *ama1*-D3 (squares) and *cpmp* (diamonds). Different haplotypes of each marker are represented by the various colours of symbols. A solid line connecting haplotypes represents a persisting haplotype. Dotted lines connect haplotypes that were intermittently undetected, e.g. persisting haplotypes under the detection threshold or reinfection of the same haplotype. Vertical dotted lines represent sampling dates. The colour of these lines indicate whether the sample was *P. falciparum* positive (red) or negative (gray) by qPCR. Red triangle point down represent treatment. Y-axis, within-host haplotype frequency.

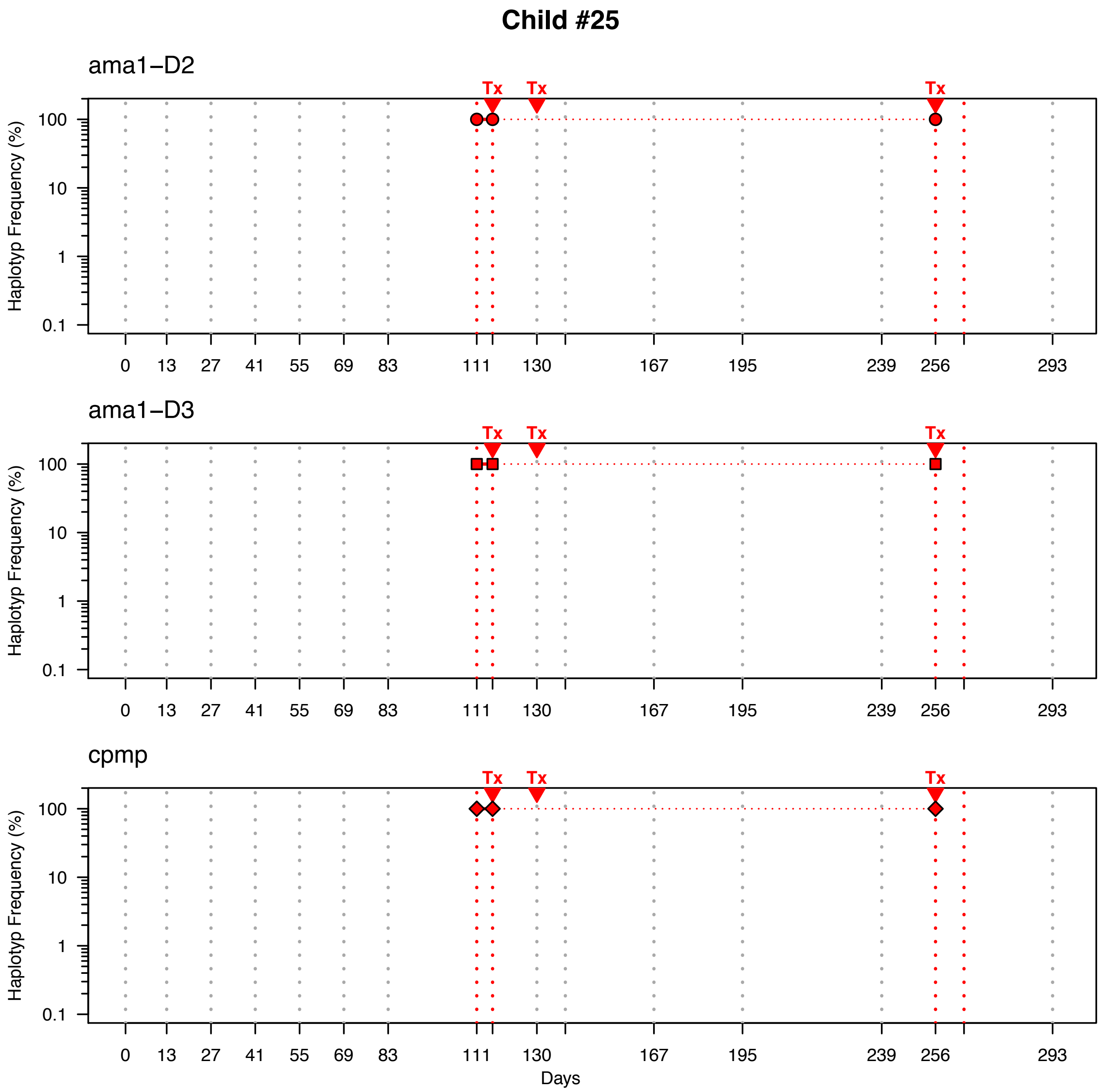

Supplementary Figure S31: Dynamics of within-host haplotype frequencies tracked by Amp-Seq markers in longitudinal samples from 33 children. For each child 3 markers were genotyped, which are distinguished by different shapes: *ama1*-D2 (circles), *ama1*-D3 (squares) and *cpmp* (diamonds). Different haplotypes of each marker are represented by the various colours of symbols. A solid line connecting haplotypes represents a persisting haplotype. Dotted lines connect haplotypes that were intermittently undetected, e.g. persisting haplotypes under the detection threshold or reinfection of the same haplotype. Vertical dotted lines represent sampling dates. The colour of these lines indicate whether the sample was *P. falciparum* positive (red) or negative (gray) by qPCR. Red triangle point down represent treatment. Y-axis, within-host haplotype frequency.

Child #26

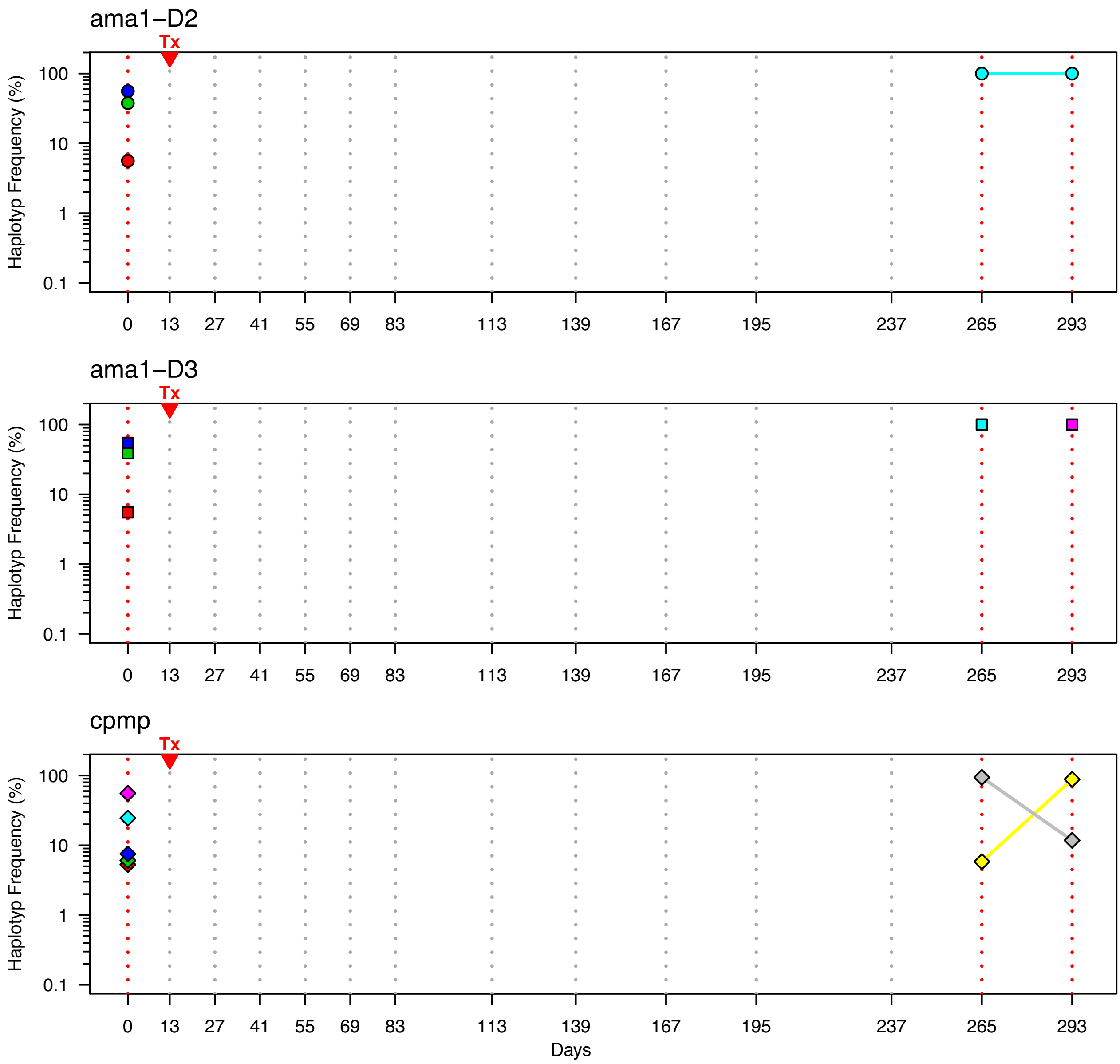

Supplementary Figure S32: Dynamics of within-host haplotype frequencies tracked by Amp-Seq markers in longitudinal samples from 33 children. For each child 3 markers were genotyped, which are distinguished by different shapes: *ama1*-D2 (circles), *ama1*-D3 (squares) and *cpmp* (diamonds). Different haplotypes of each marker are represented by the various colours of symbols. A solid line connecting haplotypes represents a persisting haplotype. Dotted lines connect haplotypes that were intermittently undetected, e.g. persisting haplotypes under the detection threshold or reinfection of the same haplotype. Vertical dotted lines represent sampling dates. The colour of these lines indicate whether the sample was *P. falciparum* positive (red) or negative (gray) by qPCR. Red triangle point down represent treatment. Y-axis, within-host haplotype frequency.

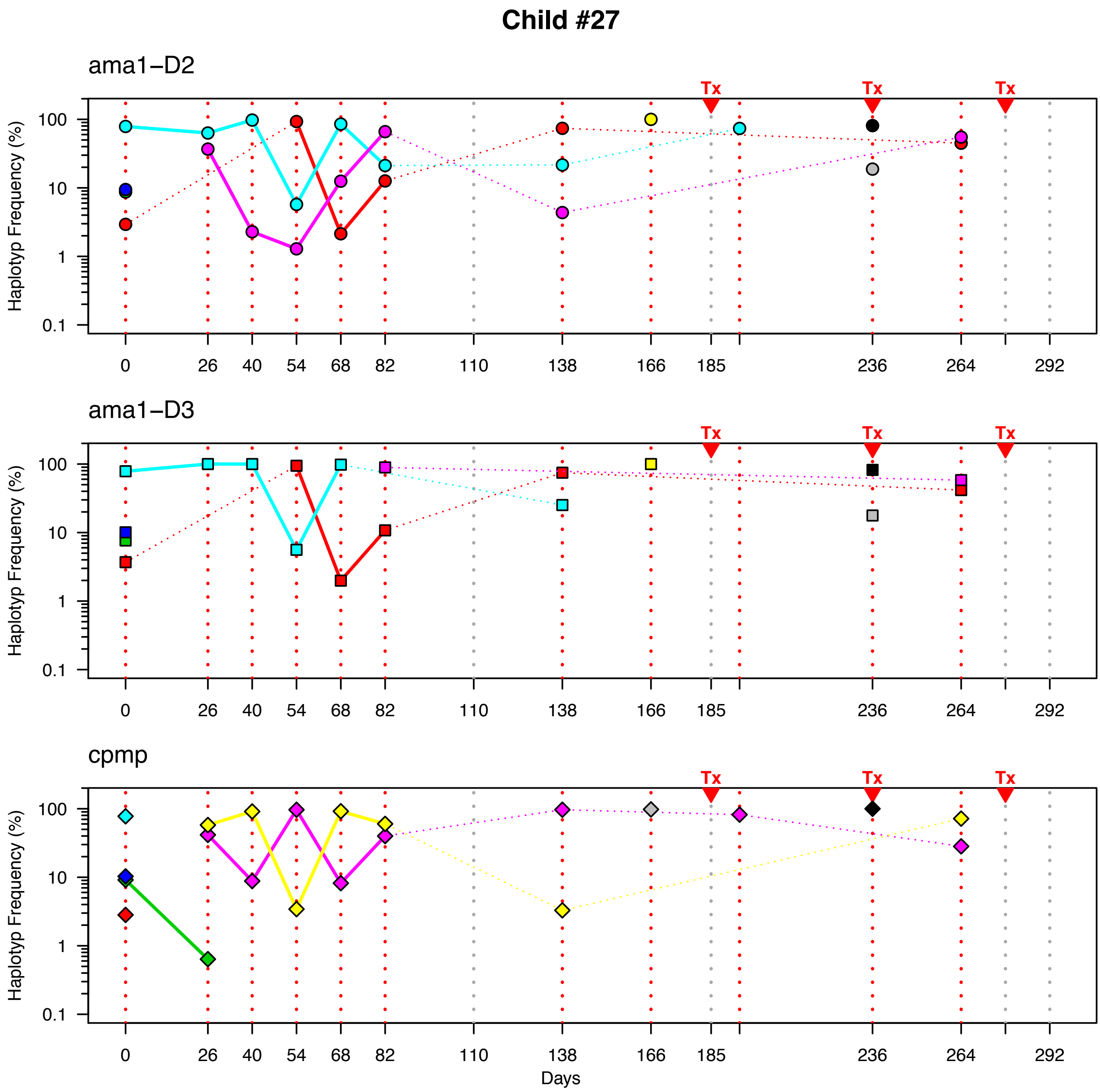

Supplementary Figure S33: Dynamics of within-host haplotype frequencies tracked by Amp-Seq markers in longitudinal samples from 33 children. For each child 3 markers were genotyped, which are distinguished by different shapes: *ama1*-D2 (circles), *ama1*-D3 (squares) and *cpmp* (diamonds). Different haplotypes of each marker are represented by the various colours of symbols. A solid line connecting haplotypes represents a persisting haplotype. Dotted lines connect haplotypes that were intermittently undetected, e.g. persisting haplotypes under the detection threshold or reinfection of the same haplotype. Vertical dotted lines represent sampling dates. The colour of these lines indicate whether the sample was *P. falciparum* positive (red) or negative (gray) by qPCR. Red triangle point down represent treatment. Y-axis, within-host haplotype frequency.

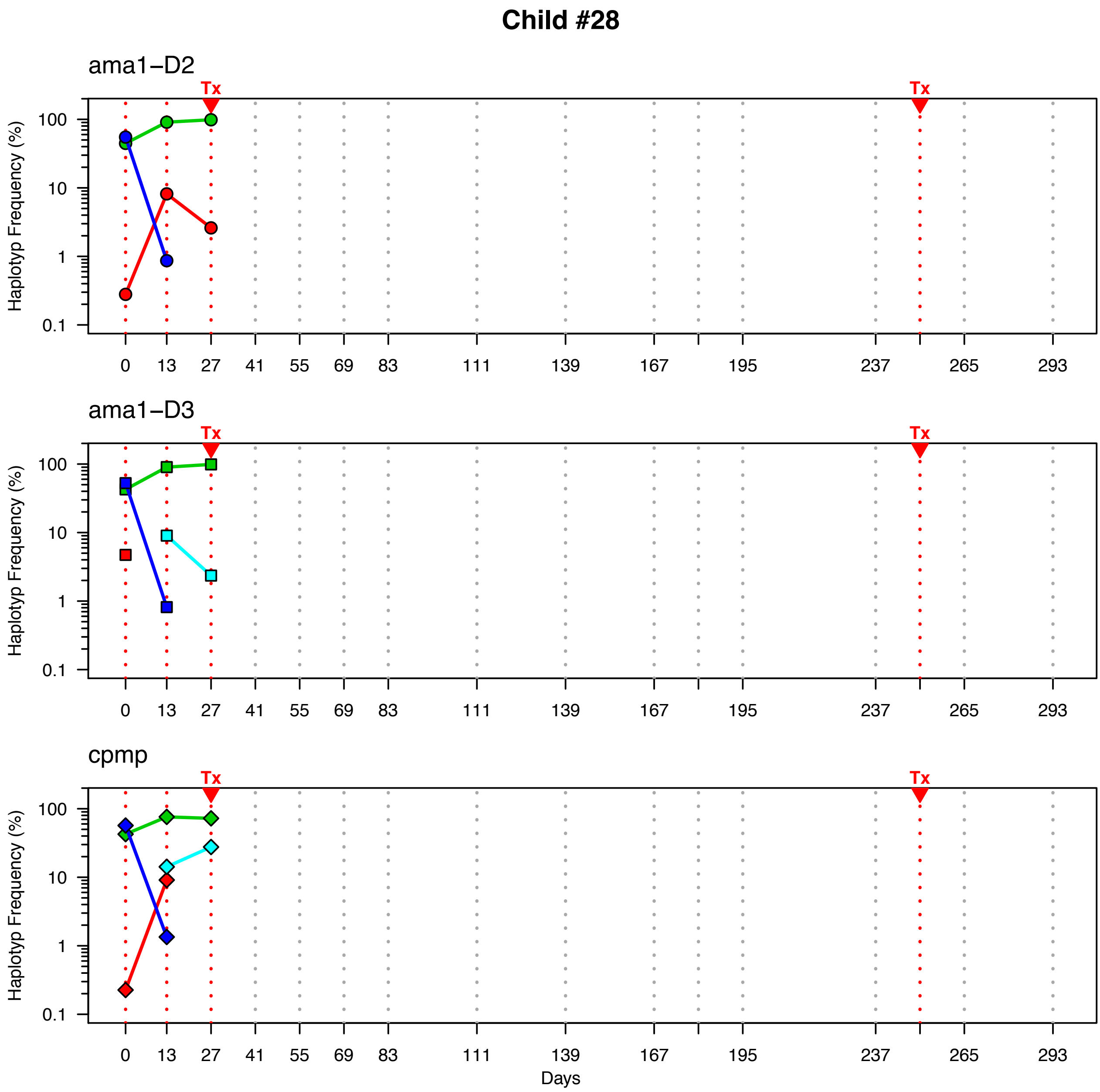

Supplementary Figure S34: Dynamics of within-host haplotype frequencies tracked by Amp-Seq markers in longitudinal samples from 33 children. For each child 3 markers were genotyped, which are distinguished by different shapes: *ama1*-D2 (circles), *ama1*-D3 (squares) and *cpmp* (diamonds). Different haplotypes of each marker are represented by the various colours of symbols. A solid line connecting haplotypes represents a persisting haplotype. Dotted lines connect haplotypes that were intermittently undetected, e.g. persisting haplotypes under the detection threshold or reinfection of the same haplotype. Vertical dotted lines represent sampling dates. The colour of these lines indicate whether the sample was *P. falciparum* positive (red) or negative (gray) by qPCR. Red triangle point down represent treatment. Y-axis, within-host haplotype frequency.

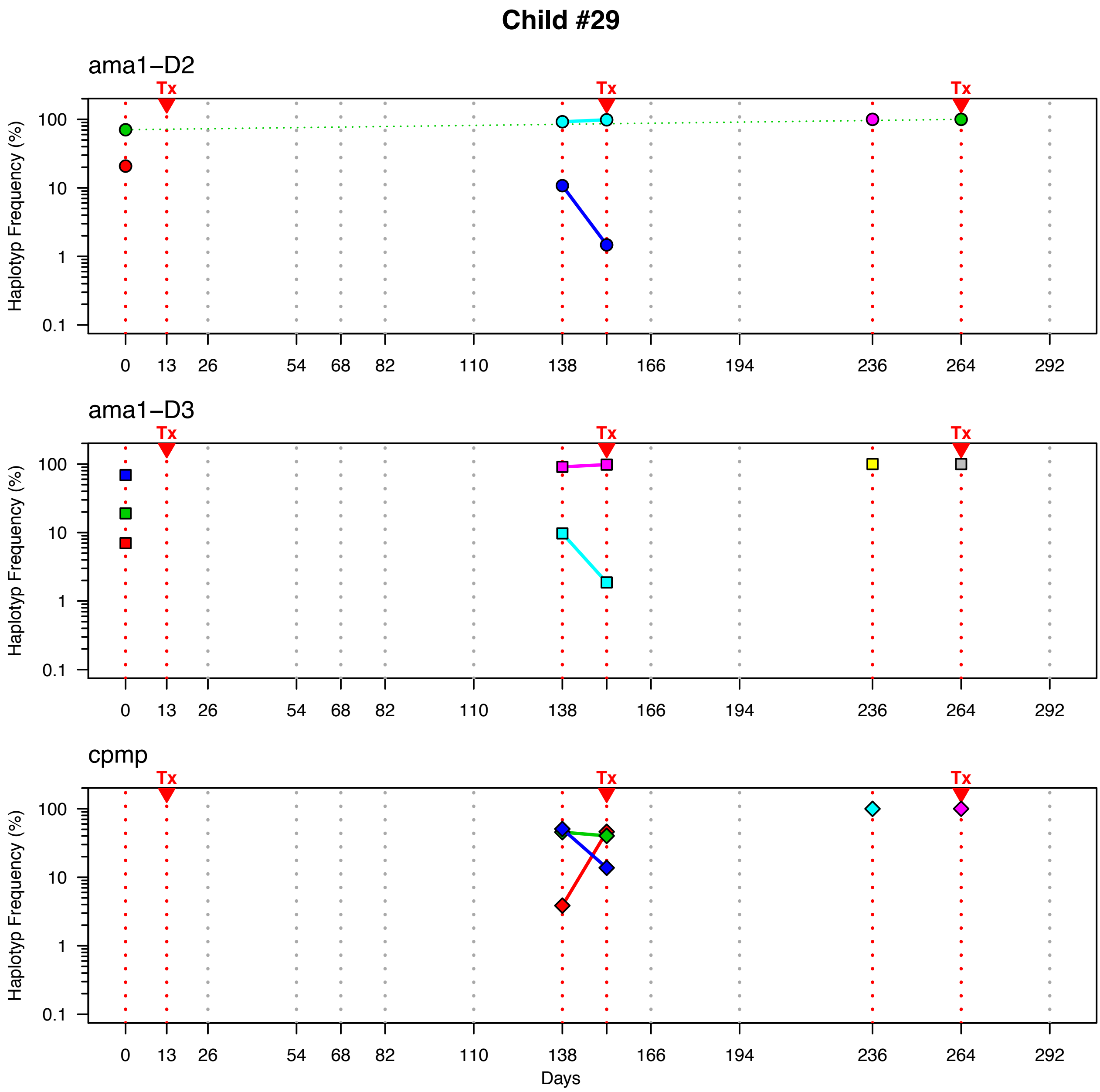

Supplementary Figure S35: Dynamics of within-host haplotype frequencies tracked by Amp-Seq markers in longitudinal samples from 33 children. For each child 3 markers were genotyped, which are distinguished by different shapes: *ama1*-D2 (circles), *ama1*-D3 (squares) and *cpmp* (diamonds). Different haplotypes of each marker are represented by the various colours of symbols. A solid line connecting haplotypes represents a persisting haplotype. Dotted lines connect haplotypes that were intermittently undetected, e.g. persisting haplotypes under the detection threshold or reinfection of the same haplotype. Vertical dotted lines represent sampling dates. The colour of these lines indicate whether the sample was *P. falciparum* positive (red) or negative (gray) by qPCR. Red triangle point down represent treatment. Y-axis, within-host haplotype frequency.

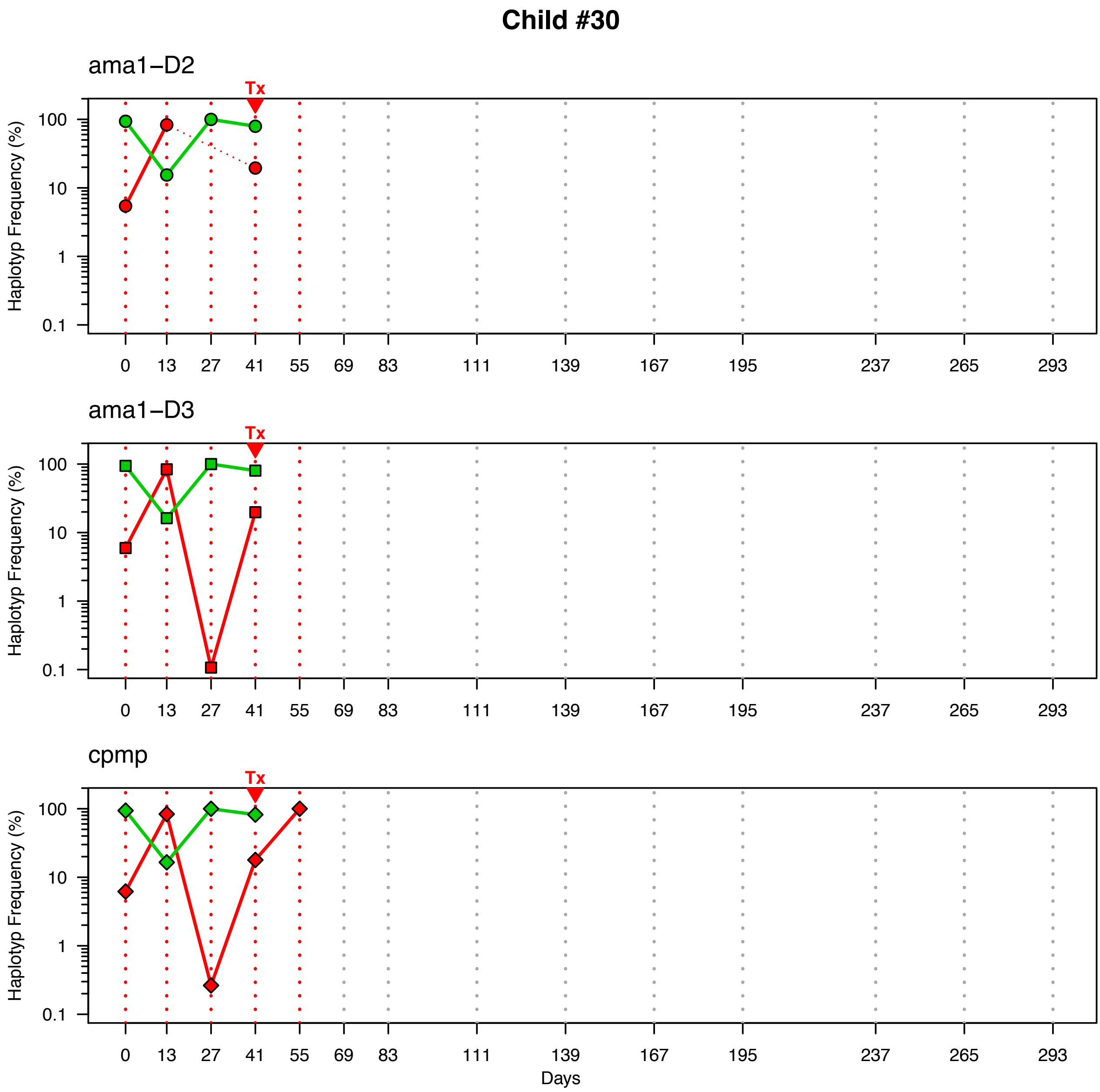

Supplementary Figure S36: Dynamics of within-host haplotype frequencies tracked by Amp-Seq markers in longitudinal samples from 33 children. For each child 3 markers were genotyped, which are distinguished by different shapes: *ama1*-D2 (circles), *ama1*-D3 (squares) and *cpmp* (diamonds). Different haplotypes of each marker are represented by the various colours of symbols. A solid line connecting haplotypes represents a persisting haplotype. Dotted lines connect haplotypes that were intermittently undetected, e.g. persisting haplotypes under the detection threshold or reinfection of the same haplotype. Vertical dotted lines represent sampling dates. The colour of these lines indicate whether the sample was *P. falciparum* positive (red) or negative (gray) by qPCR. Red triangle point down represent treatment. Y-axis, within-host haplotype frequency.

Child #31

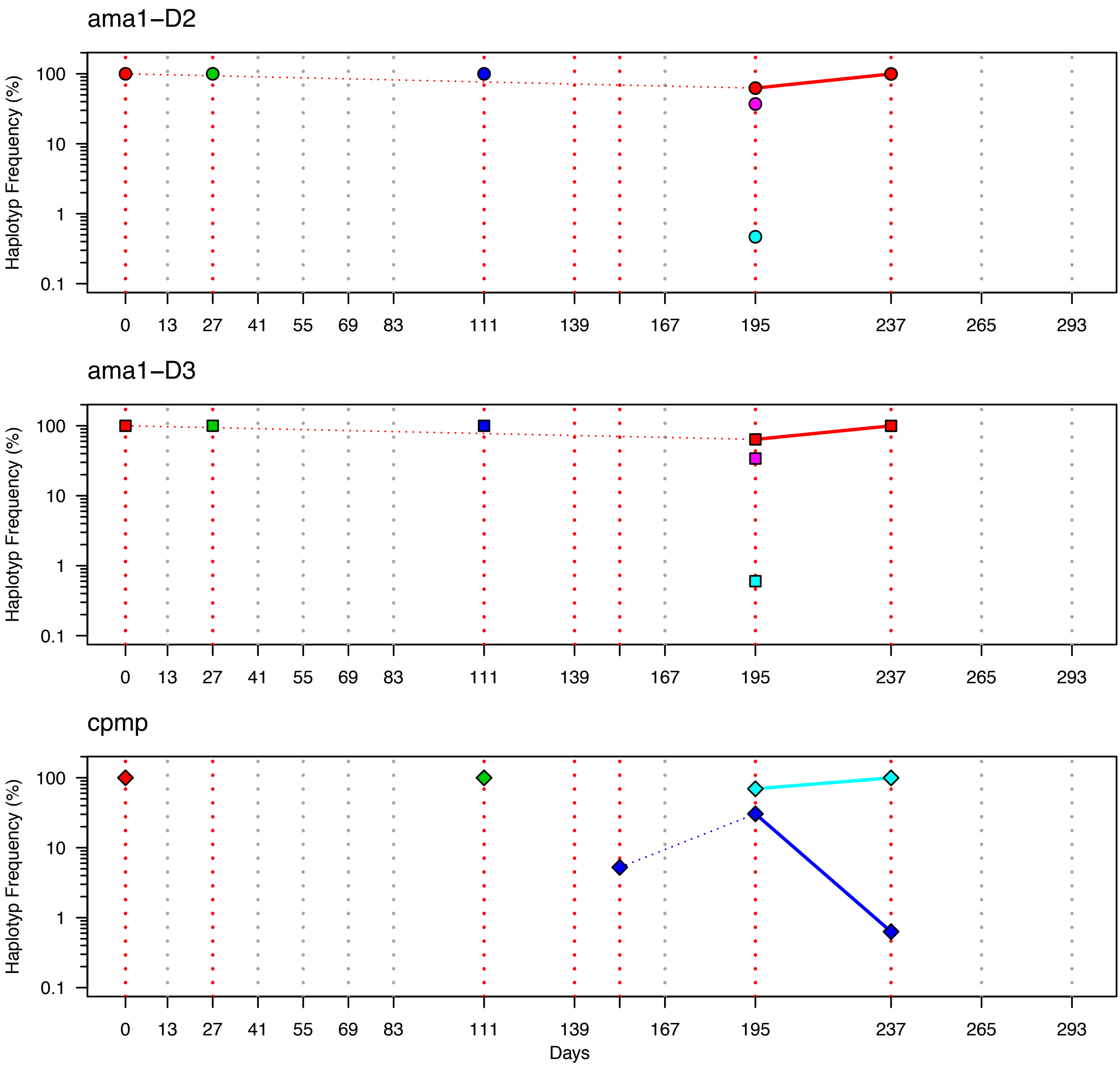

Supplementary Figure S37: Dynamics of within-host haplotype frequencies tracked by Amp-Seq markers in longitudinal samples from 33 children. For each child 3 markers were genotyped, which are distinguished by different shapes: *ama1*-D2 (circles), *ama1*-D3 (squares) and *cpmp* (diamonds). Different haplotypes of each marker are represented by the various colours of symbols. A solid line connecting haplotypes represents a persisting haplotype. Dotted lines connect haplotypes that were intermittently undetected, e.g. persisting haplotypes under the detection threshold or reinfection of the same haplotype. Vertical dotted lines represent sampling dates. The colour of these lines indicate whether the sample was *P. falciparum* positive (red) or negative (gray) by qPCR. Red triangle point down represent treatment. Y-axis, within-host haplotype frequency.

Child #32

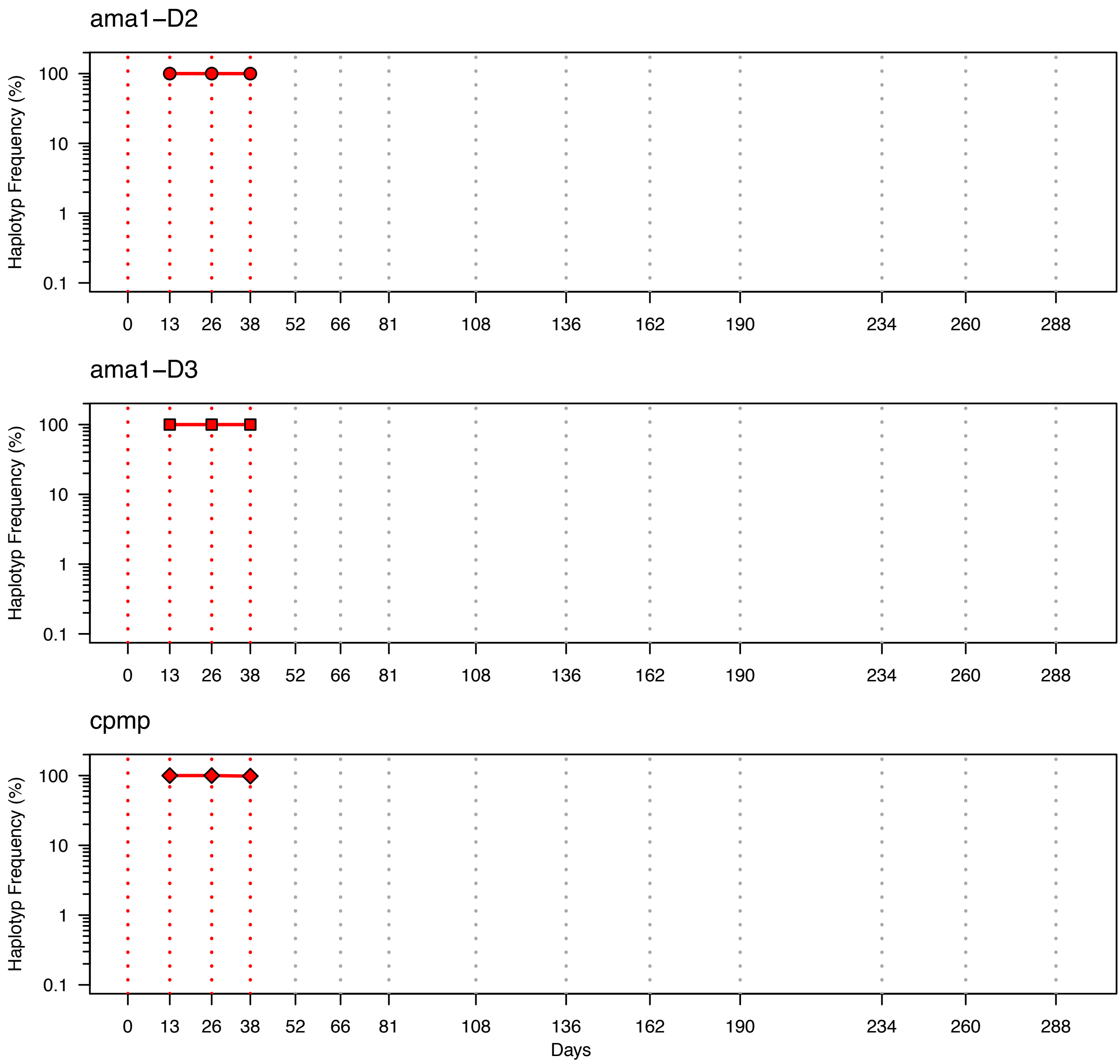

Supplementary Figure S38: Dynamics of within-host haplotype frequencies tracked by Amp-Seq markers in longitudinal samples from 33 children. For each child 3 markers were genotyped, which are distinguished by different shapes: *ama1*-D2 (circles), *ama1*-D3 (squares) and *cpmp* (diamonds). Different haplotypes of each marker are represented by the various colours of symbols. A solid line connecting haplotypes represents a persisting haplotype. Dotted lines connect haplotypes that were intermittently undetected, e.g. persisting haplotypes under the detection threshold or reinfection of the same haplotype. Vertical dotted lines represent sampling dates. The colour of these lines indicate whether the sample was *P. falciparum* positive (red) or negative (gray) by qPCR. Red triangle point down represent treatment. Y-axis, within-host haplotype frequency.

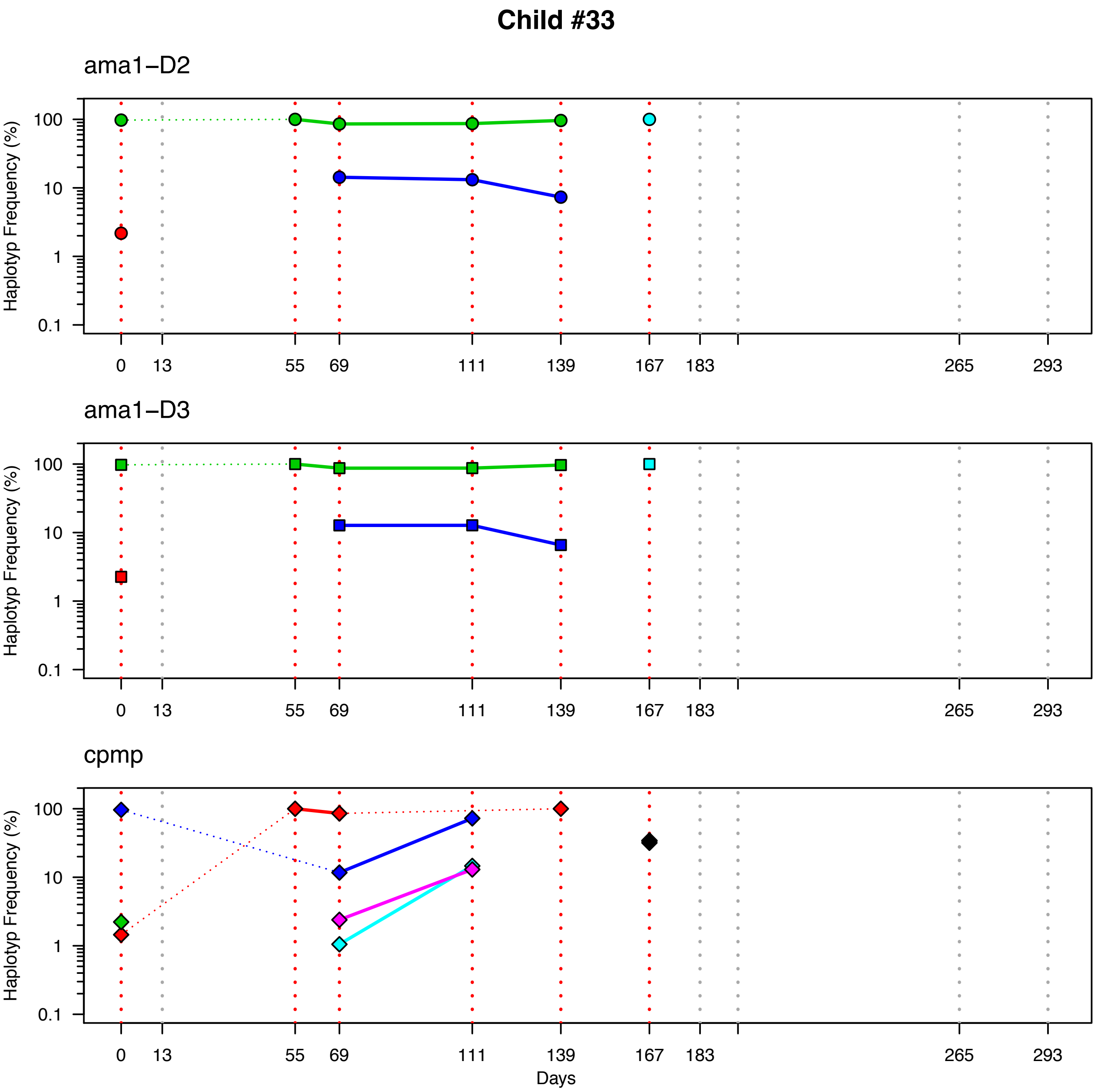

Supplementary Figure S39: Dynamics of within-host haplotype frequencies tracked by Amp-Seq markers in longitudinal samples from 33 children. For each child 3 markers were genotyped, which are distinguished by different shapes: *ama1*-D2 (circles), *ama1*-D3 (squares) and *cpmp* (diamonds). Different haplotypes of each marker are represented by the various colours of symbols. A solid line connecting haplotypes represents a persisting haplotype. Dotted lines connect haplotypes that were intermittently undetected, e.g. persisting haplotypes under the detection threshold or reinfection of the same haplotype. Vertical dotted lines represent sampling dates. The colour of these lines indicate whether the sample was *P. falciparum* positive (red) or negative (gray) by qPCR. Red triangle point down represent treatment. Y-axis, within-host haplotype frequency.

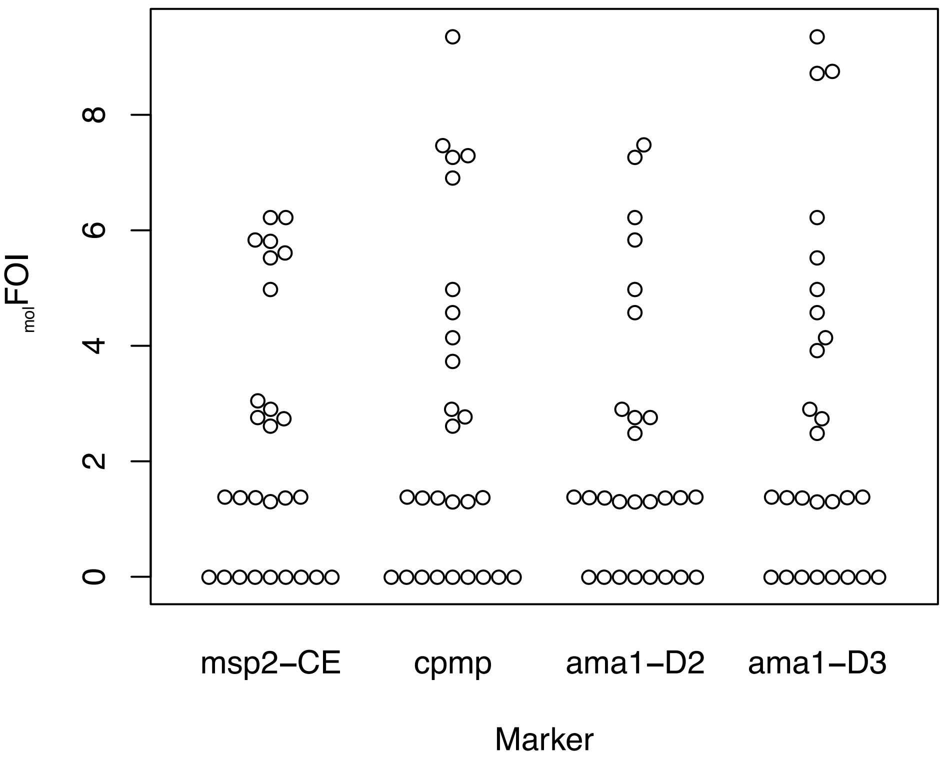

Supplementary Figure S40: Frequency distribution of molecular force of infection ($_{mol}$FOI) by marker. A total of 117 samples from 27 individuals (represented by circles) (on average 4.3 samples per individual [min: 2, max: 7]) were used to estimate molecular force of infection ($_{mol}$FOI, i.e. new clones per year-at-risk).

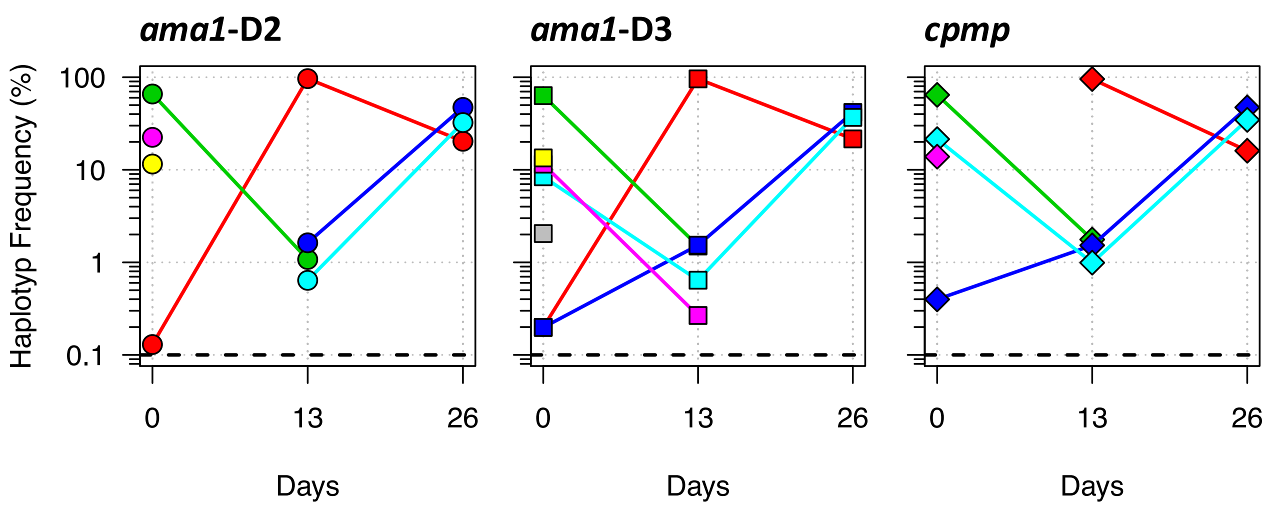

Multi-locus haplotype names	Day 0 ama1-D2 %	Day 0 ama1-D3 %	Day 0 cpmp %	Day 13 ama1-D2 %	Day 13 ama1-D3 %	Day 13 cpmp %	Day 32 ama1-D2 %	Day 32 ama1-D3 %	Day 32 cpmp %
Haplotype 1	65.9	63.3	64.4	1.1	1.5	1.8			
Haplotype 2	0.1	0.2		96.6	96	95.6	20.3	21.6	16.1
Haplotype 3		0.2	0.4	1.6	1.5	1.5	47.3	41.6	47.2
Haplotype 4		8.4	21.4	0.6	0.6	1	32.4	36.8	34.6
Haplotype 5	22.4								
Haplotype 6	11.5								
Haplotype 7		11.5			0.3				
Haplotype 8		13.4							
Haplotype 9		2.0							
Haplotype 10			13.9						

Supplementary Figure S41: Within-host haplotype frequency of Amp-Seq markers in longitudinal samples from 1 child representing an unresolvable multi-locus haplotype. Inserted table lists within-host haplotype frequencies for all markers with a possible solution of partly established multi-locus haplotypes for the major haplotypes. Multi-locus haplotypes 1-3 match well in frequencies of individual haplotypes at day 0, 13 and 32. In contrast, multi-locus haplotype 4 does not match in frequencies of individual haplotypes at day 0. This could be explained by a complex shared haplotype situation with one or several clones detected only at day 0 and 13, e.g. haplotypes 5-10. Solid lines represent persisting haplotypes.

Supplemental Tables

Supplementary Table S1: PCR Primer sequence for Amp-Seq and *msp2*-CE genotyping and sequence library preparation.

**Primer for primary PCR**	
cpmp_prim_F	CGATACAGGACATATAGA
cpmp_prim_R	TTCAATAACATTTACTAGG
Pfama1_F5	TGCGTATTATTATTGAGC
Pfama1_R613	GTGTTGTATGTGATGCTC
**Primer for nested PCR**	
ama1_D2_F_Linker	GTGACCTATGAACTCAGGAGTC**GGTCCTAGATATTGTAATAAAG**
ama1_D2_R_Linker	CTGAGACTTGCACATCGCAGC**CATGTTGGTTTGACATTAAA**
ama1_D3_F_Linker	GTGACCTATGAACTCAGGAGTC**TACTACTGCTTTGTCCCATC**
ama1_D3_R_Linker	CTGAGACTTGCACATCGCAGC**TCAGGATCTAACATTTCATC**
cpmp_F_Linker	GTGACCTATGAACTCAGGAGTC**CATAAGTCATTAAAATTTATGGAT**
cpmp_R_Linker	CTGAGACTTGCACATCGCAGC**CGTTACTATCAAGATCGTTAATATC**
**Primer for msp2 CE genotyping**	
msp2_S2_fw	GAAGGTAATTAAAACATTGTC
msp2_S3_rev	GAGGGATGTTGCTGCTCCACAG
msp2_S1-fw	GCTTATAATATGAGTATAAGGAGAA
msp2_FC27-rev	GCATTGCCAGAACTTGAA
msp2_3D7-rev	CTGAAGAGGTACTGGTAGA
**Primer for sequence library PCR (XXXXXX=barcode)**	
Forward	AATGATACGGCGACCACCGAGATCTACACTCTTTCCCTACACGACGC TCTTCCGATCTXXXXXXXXGTGACCTATGAACTCAGGAGTC
Reverse	CAAGCAGAAGACGGCATACGAGATCGGTCTCGGCATTCCTGCTGAAC CGCTCTTCCGATCTXXXXXXXXCTGAGACTTGCACATCGCAGC

**Forward barcode**		**Reverse barcode**	
Fwd_1	TAGATCGC	Rev_1	TAAGGCGA
Fwd_2	CTCTCTAT	Rev_2	CGTACTAG
Fwd_3	TATCCTCT	Rev_3	AGGCAGAA
Fwd_4	AGAGTAGA	Rev_4	TCCTGAGC
Fwd_5	GTAAGGAG	Rev_5	GGACTCCT
Fwd_6	ACTGCATA	Rev_6	TAGGCATG
Fwd_7	AAGGAGTA	Rev_7	CTCTCTAC
Fwd_8	CTAAGCCT	Rev_8	CAGAGAGG
Fwd_13	TGGTGGTA	Rev_9	GCTACGCT
Fwd_14	TTCACGCA	Rev_10	CGAGGCTG
Fwd_15	AGCACCTC	Rev_11	AAGAGGCA
Fwd_16	CAAGGAGC	Rev_12	GTAGAGGA
Fwd_17	ATTGGCTC	Rev_13	ATGCCTAA
Fwd_18	CACCTTAC	Rev_14	ACGCTCGA
Fwd_19	CTAAGGTC	Rev_15	AGTCACTA
Fwd_20	GAACAGGC	Rev_16	ATCCTGTA
		Rev_17	CGCATACA
		Rev_18	CTGGCATA
		Rev_19	GATAGACA
		Rev_20	GCTAACGA
		Rev_21	GTGTTCTA
		Rev_22	TCCGTCTA
		Rev_23	CCTAATCC
		Rev_24	GACAGTGC

Supplementary Table S2: Location and size of the amplicons.

	cpmp	*ama1*-D2	*ama1*-D3
**From**	1895	775	1281
**To**	2324	1253	1796
**Size**	430	479	516

Supplementary Table S3: Summery of sequence coverage (total read numbers) by Amp-Seq marker.

	cpmp	*ama1*-D2	*ama1*-D3
**1st Qu.**	247	2292	2997
**Median**	794	3386	4716
**Mean**	1117	3682	5189
**3rd Qu.**	1632	5143	6906
**Max**	6376	11570	34240

Supplementary Table S4: Summary of multi-locus haplotype (MLH) inference based on longitudinal samples from 33 children.

**Status of MLH inference**	**Samples**	**Multi-locus haplotypes**	**Single-locus haplotypes**		
			cpmp	***ama1*-D2**	***ama1*-D3**
	**n**	**n**	**n**	**n**	**n**
**Full established MLH**	78 [1]	116 [1]	116	103	103
**Partly established MLH** [2]	49	64	135	130	126
**Unresolvable MLH** [3]	8	0	20	18	18
**Incomplete datasets** [4]	13	0	7	11	11
**Total**	140	180	258 [5]	244 [5]	240 [5]

n number of samples or haplotypes.

[1] 45 out of 78 samples with fully established multi-locus haplotypes were single clone infections.

[2] Samples were multi-locus haplotypes could be established for some but not for all clones of a sample.

[3] Samples were no multi-locus haplotype could be established.

[4] Samples with missing genotyping results for any of the markers.

[5] Total number of parasite clones detected in 140 samples was 277.

Supplementary Table S5: Overview of sample selection criteria applied for different types of analyses.

Analysis Type	Samples n	Children n	Selection Criteria
Baseline H_e and MOI	33	33	Baseline (or first bleed available) sample.
Multi-locus H_e	46	33	Samples with a resolvable multi-locus haplotype that were separated by a treatment plus ≥2 consecutive *P. falciparum* negative samples from the same child.
$_{mol}$FOI	117	27	Children with a complete set of replicates.
Sensitivity and false discovery rate	48	12	Children that did not received antimalarial treatment during the timespan analysed and harboured at least one haplotype that was detected at 3 consecutive bleeds.
Reproducibility	139	33	True-positive haplotypes.

Supplementary Table S6: Numbers of haplotypes missed by either of the molecular markers due to imperfect detection either at beginning of infection**, in any intermediate sample, or prior to haplotype clearance.** Haplotypes from 48 longitudinal samples from 12 children were classified into true-positive (TP) and false-negative haplotypes. Two types of false-negative haplotypes (missed clones) can be differentiated: (FN_i) False-negative haplotypes detected but below cut-off criteria and (FN_{ii}) false-negative haplotypes not detected but imputed.

Marker	Baseline sample n	Any intermediate sample n	Sample prior to clearance n
**TP haplotypes**			
msp2-CE	29	34	23
cpmp	39	45	31
ama1-D2	36	44	29
ama1-D3	36	43	29
**FN_i haplotypes**			
msp2-CE	2	6	2
cpmp	1	0	3
ama1-D2	0	1	2
ama1-D3	1	0	3
**FN_{ii} haplotypes**			
msp2-CE	n/a [1]	5	n/a [1]
cpmp	0	2	1
ama1-D2	1	0	0
ama1-D3	1	2	0

[1] FN_{ii} haplotypes cannot be imputed at the beginning of infection or prior to clearance for marker *msp2*-CE.

Supplementary Table S7: Reproducibility of haplotypes by classification in technical replicates.

	msp2-CE			*cpmp*			*ama1*-D2			*ama1*-D3		
	n_1	n_2	q	n_1	n_2	q	n_1	n_2	q	n_1	n_2	q
FN	7	0	-	1	0	-	1	0	-	0	0	-
FP	133	0	-	39	0	-	24	0	-	27	0	-
TP	38	158	0.89	38	233	0.92	37	229	0.93	28	230	0.94
Total	178	158	0.64	78	233	0.86	62	229	0.88	55	230	0.89

n_1 number of clones detected only with one of the replicates.
n_2 number of clones detected with both replicates.
q detectability as descripted in Bretscher et al. 2010.

Supplementary Table S8: Reproducibility of true-positive haplotypes in technical replicates. Reproducibility only decreased when clone densities fell below 1000 copies 18S rRNA gene per μl whole blood and/or within-host frequency below 1% (Supplementary Figure S5).

	cpmp			*ama1*-D2			*ama1*-D3		
	n_1	n_2	q	n_1	n_2	q	n_1	n_2	q
**Haplotype density (copies/μl)**									
>1000	12	143	0.960	10	138	0.965	4	140	0.986
100-1000	6	52	0.945	10	50	0.909	6	50	0.943
10-100	12	19	0.760	11	22	0.800	11	21	0.792
≤10	5	11	0.815	5	10	0.800	6	11	0.786
**Within-host haplotype frequency (%)**									
>10	24	169	0.934	25	162	0.928	16	168	0.955
1-10	5	55	0.957	4	49	0.961	5	48	0.950
≤1	9	9	0.667	8	18	0.818	7	14	0.800
**Lower sequence coverage of replicates**									
>1000	1	68	0.993	10	215	0.977	10	214	0.977
100-1000	9	144	0.970	2	9	0.900	3	5	0.769
<100	28	21	0.600	25	5	0.286	15	11	0.595

n_1 number of clones detected only with one of the replicates.
n_2 number of clones detected with both replicates.
q detectability as descripted in Bretscher et al. 2010.

Supplemental Text

Example of multi-locus haplotype inference

Below an example of *P. falciparum* infection dynamics is shown for one child in great detail to illustrate our strategy for inferring a multi-locus haplotype that combines SNP data from three molecular markers *ama1*-D2, *ama1*-D3, and *cpmp.* Within-host haplotype frequency data of the example is shown in Supplementary Table S8 and corresponding graphic illustration in Supplementary Figure S42.

Supplementary Table S8: Within-host haplotype frequencies (WHHF) in percent of individual Amp-Seq markers observed in longitudinal samples from one child. Haplotypes of individual markers (termed alleles) are sorted by WHHF of day 0. **Haplotypes 1-4** represent multi-loci haplotypes composed of one allele of each of the 3 markers.

Multi-locus haplotype names	Day 0			Day 13			Day 32		
	***ama1*-D2** %	***ama1*-D3** %	***cpmp*** %	***ama1*-D2** %	***ama1*-D3** %	***cpmp*** %	***ama1*-D2** %	***ama1*-D3** %	***cpmp*** %
**Haplotype 1**	48.5	44.0	48.0	94.4	92.6	94.3	0.13	0.06	
**Haplotype 2**	40.2	41.5	41.4	2.61	3.29	2.62	6.77	7.42	7.10
**Haplotype 3**	11.3	11.6	10.6	0.53	0.73	0.56	93.1	92.5	92.9
**Haplotype 4**	-	-	-	2.71	2.83	2.49	0.12	0.07	0.04

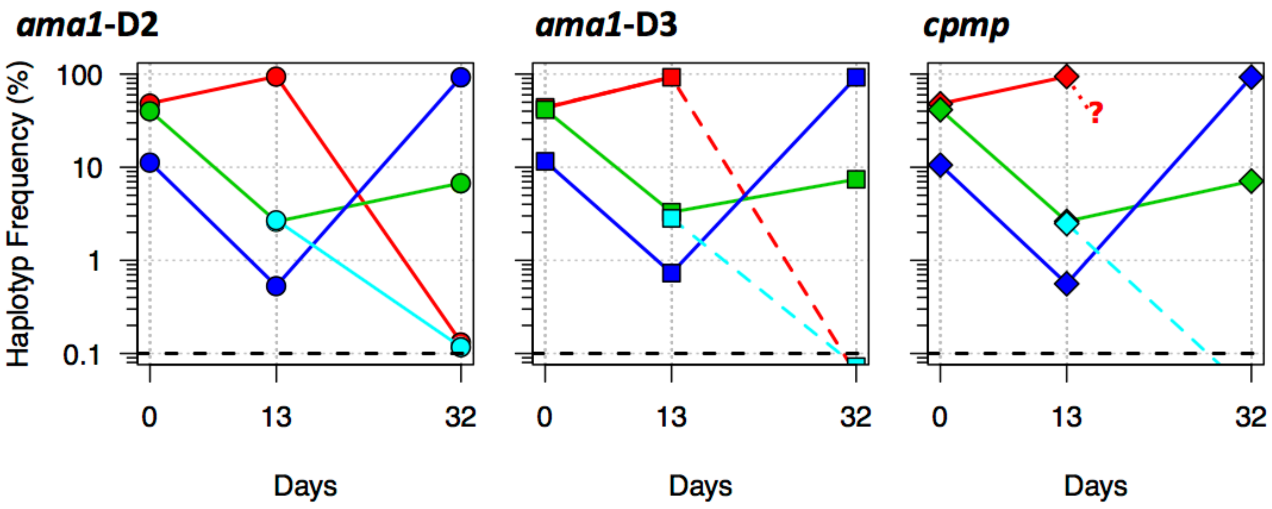

Supplementary Figure S42: Within-host haplotype frequencies of Amp-Seq markers in longitudinal samples from one child. Multi-locus haplotypes have the same colour-code in figures representing the 3 molecular markers. Solid lines represent persisting haplotypes above cut-off criteria (true-positive haplotypes). Dashed line represents persisting haplotypes falling below cut-off criteria (false-negative haplotypes detected below cut-off criteria). Dotted line and question mark indicate a false-negative haplotype that was not detected but could be imputed based on the established multi-locus haplotypes from the preceding sample. Black dashed line represents cut-off criteria of the Amp-Seq genotyping method.

The inference of multi-marker haplotypes started with identification of alleles that belong to the dominant parasite clone. A dominant Haplotype was defined by a within-host haplotype frequencies (WHHF) >54%.

Inference of multi-marker Haplotypes at Day 0

At Day 0 of this example, 2 different alleles per marker occurred at similar WHHF (listed by marker in Supplemental Table S8. At Day 0 no dominant Haplotype was evident, therefore any increase or decrease of in WHHF of these alleles at Day13 was interrogated: one allele of each of the 3 markers showed an increase of approx. +46%, while the remaining 3 alleles of similar frequency revealed a decrease by approx. -38%. Based on these recoded frequency changes we combined those alleles from each marker, which all increased by approx. +46%, into multi-locus **Haplotype 1** (Supplementary Figure S9, Day 0 in red).

Alleles that constituted **Haplotype 1** were not considered in next steps of inference. Additional multi-locus haplotypes of Day 0 were inferred by combining the alleles of similar frequency which showed a decrease in WHHF for all 3 markers of approx. -38%, thus defining multi-locus **Haplotype 2** (Supplementary Figure S9, Day 0 in green). For the next steps of inference, all alleles associated with multi-locus **Haplotypes** 1 and **2** were no more considered. The remaining alleles constituted multi-locus **Haplotype 3** with ~11% WHHF for all markers (Supplementary Figure S9, Day 0 in blue).

Multi-marker Haplotypes at Day 13

The dominant alleles in all 3 markers of the Day 13 sample were consistent with multi-locus **Haplotype 1** characterized by ~93% WHHF for all 3 markers (Supplementary Figure S9, Day 13 in red). Again this multi-locus haplotype was no more considered in the next steps of Day 13 haplotype inference. Next two multi-locus haplotypes with similar WHHF were observed. In agreement with allele combinations found at Day 0, multi-locus **Haplotype 2** was identified by an increase of these alleles at Day 32 of approx. +4% (Supplementary Figure S9, Day 13 in green). After excluding alleles constituent multi-locus **Haplotypes 1** and **2** an additional new multi-locus **Haplotype 4** with similar WHHF as **Haplotype 2** was found (Supplementary Figure S42 Day 13 in light blue). The remaining alleles, all with frequencies below 1%, corresponded to multi-locus **Haplotype 3** (Supplementary Figure S9, Day 13 in blue).

Multi-marker Haplotypes at Day 32

The dominant clone in the Day 32 sample corresponds to multi-locus **Haplotype 3**, characterized in this sample by a steep increase of, ~93% WHHF for all markers (Supplementary Figure S9, Day 32 in blue). Alleles of this dominant clones are no more considered in the next step of inference. The dominant clone in this step corresponds to **Haplotype 2** with ~7% WHHF for all markers (Supplementary Figure S9, Day 32 in green). In the next step all alleles of **Haplotypes 3** and **2** were no more considered. But no further multi-locus haplotypes could be established, as WHHF of the remaining alleles were below the 0.1% WHHF cut-off criteria for some of the markers. However, as the inferred multi-locus haplotypes of Day 0, 13 and 32 match for all samples and marker ama1-D2 showed a WHHF above the cut-off criteria, the multi-locus Haplotypes **1** and **4** could be imputed (Supplementary Figure S9, Day 32 in light blue and red).
